# Effects of *N*-Substituents on the Functional Activities of Naltrindole Derivatives for the δ Opioid Receptor: Synthesis and Evaluation of Sulfonamide Derivatives

**DOI:** 10.3390/molecules25173792

**Published:** 2020-08-20

**Authors:** Chiharu Iwamatsu, Daichi Hayakawa, Tomomi Kono, Ayaka Honjo, Saki Ishizaki, Shigeto Hirayama, Hiroaki Gouda, Hideaki Fujii

**Affiliations:** 1 Laboratory of Medicinal Chemistry, School of Pharmacy, Kitasato University, 5-9-1, Shirokane, Minato-ku, Tokyo 108-8641, Japan; ml18101@st.kitasato-u.ac.jp (C.I.); r.tymo-hone@i.softbank.jp (T.K.); ml17117@st.kitasato-u.ac.jp (A.H.); pl17007@st.kitasato-u.ac.jp (S.I.); hirayamas@pharm.kitasato-u.ac.jp (S.H.); 2 Division of Biophysical Chemistry, Department of Pharmaceutical Sciences, School of Pharmacy, Showa University, 1-5-8 Hatanodai, Shinagawa-ku, Tokyo 142-8555, Japan; d-hayakawa@pharm.showa-u.ac.jp (D.H.); godah@pharm.showa-u.ac.jp (H.G.); 3 Medicinal Research Laboratories, School of Pharmacy, Kitasato University, 5-9-1, Shirokane, Minato-ku, Tokyo 108-8641, Japan

**Keywords:** δ opioid receptor, NTI derivative, sulfonamide, inverse agonist, neutral antagonist, agonist

## Abstract

We have recently reported that *N*-alkyl and *N*-acyl naltrindole (NTI) derivatives showed activities for the δ opioid receptor (DOR) ranging widely from full inverse agonists to full agonists. We newly designed sulfonamide-type NTI derivatives in order to investigate the effects of the *N*-substituent on the functional activities because the side chain and S=O part in the sulfonamide moiety located in spatially different positions compared with those in the alkylamine and amide moieties. Among the tested compounds, cyclopropylsulfonamide **9f** (SYK-839) was the most potent full inverse agonist for the DOR, whereas phenethylsulfonamide **9e** (SYK-901) showed full DOR agonist activity with moderate potency. These NTI derivatives are expected to be useful compounds for investigation of the molecular mechanism inducing these functional activities.

## 1. Introduction

The ligands interacting with receptors have long been classified into agonists and antagonists. However, it is now widely accepted that there are agonists, neutral antagonists, and inverse agonists. Agonists interact with receptors in the active state to induce physiological activities, whereas inverse agonists, which bind to receptors in the inactive state, can suppress the constitutive activities of receptors. The constitutive activity is characterized as activities induced by receptors in the absence of agonists [1,2,3]. Neutral antagonists themselves do not exhibit any activities and can block the functions of both agonists and inverse agonists. Recently, the relationship between some disease states and the constitutive activity of receptors, and developments of inverse agonists have been reported [4,5,6,7,8,9,10,11,12,13,14,15,16,17,18,19,20]. In the opioid field, since Costa and Herz firstly reported that peptidic ICI-174,864 showed δ opioid receptor (DOR) inverse agonist activity [21], several peptidic and non-peptidic DOR inverse agonists have been developed [4]. Possible pharmacological effects resulting from DOR inverse agonism include anorexia [22,23], short-term memory improvement [24], and antitussive effects [25]. We also recently reported several DOR inverse agonists with the skeleton of naltrindole (NTI) [24,25,26], which was developed as a selective DOR antagonist by Portoghese et al. [27,28].

**Figure 1:**
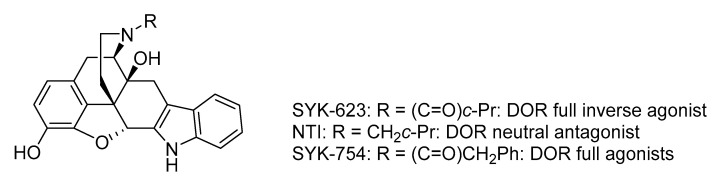
Structures of SYK-623 (DOR full inverse agonist), NTI (DOR neutral antagonist), and SYK-882 (DOR full agonist).

Interestingly, a series of compounds we reported induced activities from full inverse agonists to full agonists depending on their substituents on the 17-nitrogen atom (Figure 1) [24,25,26,29]. Amide-type compound SKY-623 bearing 17-cyclopropanecarbonyl group was a DOR full inverse agonist with high potency [25]. Well-known NTI with the 17-cyclopropylmethyl substituent was a neutral antagonist [27,28]. In contrast, SYK-754 possessing the phenylacetyl group as a 17-substituent was a DOR full agonist with moderate potency [29]. The 17-substituent of the morphinan skeleton, which is a representatively fundamental core of opioid ligands and is shared among NTI and many clinically used opioids like morphine, oxymorphone, and naloxone, is a determinant of the activities of ligands, either agonist or antagonist [30]. Especially, this tendency is observed in the ligands showing selectivity for the μ opioid receptor (MOR), which is one of the known opioid receptor types, encompassing MOR, DOR, and κ opioid receptor (KOR). For example, naltrexone and naloxone with a bulky 17-substituent like cyclopropylmethyl and allyl, respectively, are antagonists, while morphine and oxymorphone, having a small methyl group at the 17-nitrogen, are agonists. However, to the best of our knowledge, this is the first example of opioid ligands with common structures, but possessing different 17-substituents, whose activities range from full inverse agonists to full agonists. It is also worth noting that amide-type compounds SYK-623 and SYK-754 showed opioid functionality even though they lack a basic nitrogen. The basic nitrogen in opioid ligands, which is protonated under the physiological environment, is well-known to be an essential pharmacophore. Indeed, co-crystals of opioid receptors with specific antagonists or agonists have been resolved to reveal the interaction between the protonated nitrogen atom in the ligands and the aspartic acid residue in the receptor [31,32,33,34,35]. Moreover, such interaction between a ligand and the receptor has also been recently elucidated by cryo-electron microscopic analysis of the ligand-receptor complex [36,37]. The neoclerodane diterpene salvinolin A is known to be a non-nitrogenous KOR agonist [38]. However, salvinolin A has been reported to bind to the KOR in a different fashion from that of morphinan derivatives [39]. Taken together, several NTI derivatives that we have reported (Figure 1) are expected to be useful compounds to elucidate the binding modes of these structures and to investigate the effects of the *N*-substituents on the conformational changes of the DOR that induce functional activities from inverse agonists to agonists. We were therefore interested in sulfonamide-type NTI derivatives, because not only the orientation of the sidechain, but also the chemical property of the nitrogen atom in the sulfonamide moiety differs from those of tertiary alkyl or amide-type compounds. Herein, we will report the synthesis of sulfonamide-type NTI derivatives and the evaluation of their opioid activities. We also compared activities among the NTI derivatives having corresponding tertiary alkyl amine, amide, and sulfonamide moieties.

## 2. Results and Discussion

### 2.1. The Most Stable Conformations of the Corresponding Alkyl-, Amide-, and Sulfonamide-Type Compounds and the Comparison of the Basicities of Their Nitrogen Atoms

**Figure 2:**
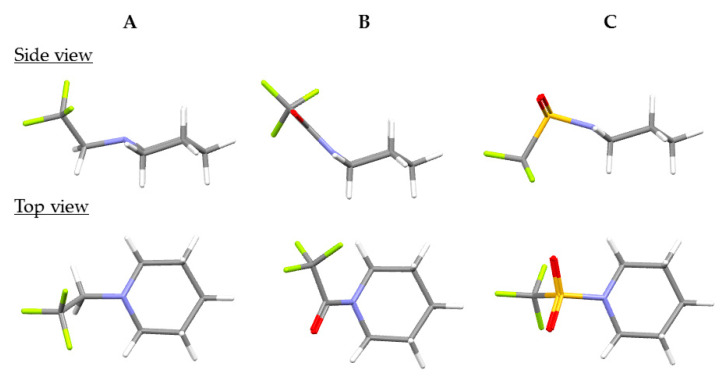
The most stable conformations of 1-(2,2,2-trifluoroethyl)piperidine (**A**), 1-(trifluoroacetyl)piperidine (**B**), and 1-(trifluoromethylsulfonyl)piperidine (**C**). The conformers were obtained by the ωB97XD/6-31G(d,p) level calculation.

In order to clarify the structural differences among alkylamines, amides, and sulfonamides, we calculated the most stable conformations of 1-(2,2,2-trifluoroethyl)piperidine (**A**), 1-(trifluoroacetyl)piperidine (**B**), and 1-(trifluoromethylsulfonyl)piperidine (**C**) as model compounds at the ωB97XD/6-31G(d,p) level (Figure 2). The orientations of trifluoromethyl parts in the three model compounds differed from each other. The C=O or S=O moieties in piperidines **B** or **C**, which were possible functionalities to interact with the receptor, were also oriented toward distinct directions. The active conformers, which interact with the target proteins, do not necessarily correspond to the most stable conformations. However, it is obvious that the relative relationship among the spatial locations of the piperidine ring, side chain, and the C=O or S=O moieties varied among the three piperidines. As the nitrogens of piperidines **A** and **B** are sp^3^ and sp^2^ hybridized, respectively, the structures of these nitrogens were trigonal pyramid and the trigonal plannar, respectively. Meanwhile, the nitrogen of piperidine **C** was observed to be slightly pyramidalized. According to the calculations by Advanced Chemistry Development (ACD/Labs) Software V11.02, the nitrogen of piperidine **A** is basic, whereas piperidines **B** and **C** have no basic nitrogens: the predicted pKa values were 6.79 ± 0.10 for **A**, −1 ± 0.20 for **B**, and −7.81 ± 0.20 for **C**. The amide-type NTI derivative SYK-623 (Figure 1) was a DOR full inverse agonist, even though it lacked a basic nitrogen, a known important pharmacophore. This observation evoked the idea that the C=O moiety might compensate for the interaction between the protonated nitrogen and the receptor. It is interesting to consider whether the S=O moieties in sulfonamide-type NTI derivatives, which were oriented differently from the C=O part in amide-type NTI derivatives, can function as a surrogate for the amide carbonyl moiety.

### 2.2. Chemical Synthesis

**Scheme 1:**
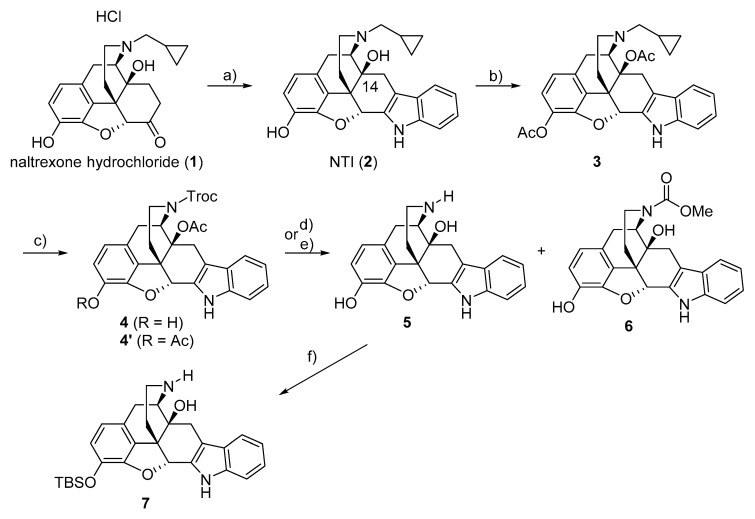
Synthesis of the key intermediate **7**. Reagents and conditions: (**a**) PhNHNH_2_·HCl, MeSO_3_H, EtOH, reflux, 99%; (**b**) Ac_2_O, 85 °C, 95%; (**c**) Troc-Cl, K_2_CO_3_, 1,1,2,2-tetrachloroethane, reflux, 92%; (**d**) 4 M NaOH aq, MeOH, reflux, **5**: 60% from **3**, **6**: 21% from **3**; (**e**) 4 M NaOH aq, THF, reflux, **5**: 72% from **3**, **6**: trace from **3**; (**f**) TBSCl, imidazole, DMF, rt, 70%.

Compound **7** [24,25], which is the key intermediate for the synthesis of the target sulfonamide-type NTI derivatives, was synthesized from naltrexone hydrochloride (**1**) (Scheme 1). Fischer indolization of **1** with phenylhydrazine hydrochloride gave NTI (**2**) [27,28] in 99% yield, which was treated with acetic anhydride to afford diacetate **3** in 95% yield. The reaction of **3** with Troc-Cl provided a mixture of compounds **4** and **4′**. Although the dealkylation with chloroformates is the usual method [40,41,42], the application of the methodology to 14-hydroxymorphinan derivatives requires the protection of the 14-hydroxy group because the cleavage reaction of the D-ring (piperidine ring) reportedly proceeded when applying the methodology to 14-hydroxy free morphinan derivatives [43]. Although the treatment with zinc and acetic acid is the usual deprotection procedure for the *N*-Troc group [44], the application of the deprotection conditions to the 14-acetoxy morphinan derivatives like **4** and **4′** sometimes facilitated the migration of the acetyl group from the 14-*O*-position to the 17-nitrogen. Therefore, we attempted to hydrolyze the 14-acetate under basic conditions using methanol as a solvent to afford compounds **5** and **6** in 60% and 21% yields, respectively. As compound **5** was unexpectedly obtained, we explored the optimal conditions of hydrolysis (see the Supporting Information for details). The treatment of a mixture of **4** and **4′** with 4 M NaOH aqueous solution using THF as a solvent selectively furnished compound **5** and methyl carbamate **6** was almost not detected. Finally, the key intermediate **7** was prepared by introduction of TBS group at the phenolic hydroxy group of **5**. Although we previously reported the synthesis of **7** from **1** [24], the synthetic method shown in Scheme 1 was four steps shorter and provided the key intermediate **7** in higher total yield than the previous method (see the Supporting Information for details). Portoghese et al. and Rice et al. independently reported the synthesis of the **5** hydrochloride from noroxymorphone in one step (see the Supporting Information for details) [45,46]. However, noroxymorphone is difficult to obtain and very expensive compared with naltrexone hydrochloride (**1**). 

**Scheme 2:**
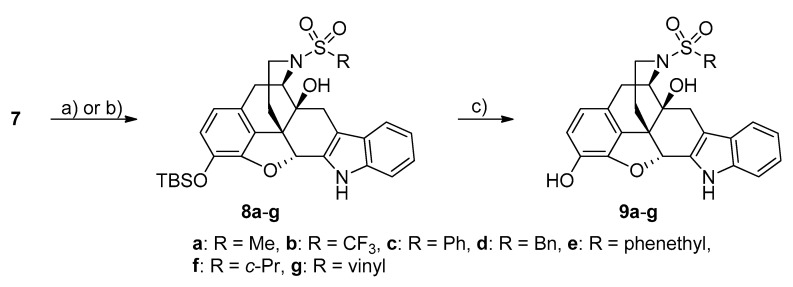
Synthesis of sulfonamide-type NTI derivatives **9**. Reagents and conditions: (**a**) RSO_2_Cl, Et_3_N, CH_2_Cl_2_, 0 °C, 46–98%; (**b**) ClCH_2_CH_2_SO_2_Cl, 0.5 M NaOH aq, CH_2_Cl_2_, 0 °C, 56% (**8g**); (**c**) 1.0 M TBAF, THF, rt, 71–96%.

Designed sulfonamide-type NTI derivatives **9** were synthesized from the key intermediate **7** (Scheme 2). Sulfonylation of **7** and the followed deprotection of TBS group gave compounds **9a**–**g**. For the synthesis of vinyl sulfonamide derivatives **8g**, 2-chloroethylsulfonyl chloride was used in the presence of NaOH instead of triethylamine. The change from triethylamine to more basic NaOH effectively expedited E2 elimination to provide the vinylsulfonyl moiety.

### 2.3. Binding Affinity and Functional Activity

The binding affinities of the synthesized compounds **9** for the opiod receptors were evaluated by competitive binding assays according to the previously reported methods [47] (Table 1). Although the binding affinity of compound **9d** for the DOR was low, the other tested compounds **9** bound to the DOR with strong to moderate affinities. Among them, compound **9f** with a cyclopropylsulfonyl group was the strongest binder to the DOR with a K_i_ value of 7.44 nM. However, its affinity for the DOR was over 10-fold lower than that of the mother compound NTI. Compounds **9c** and **9f**, which had relatively high affinities for the DOR, showed binding affinities for the KOR, but their affinities for the KOR were lower than those for the DOR. All the tested compounds exhibited almost no affinities for the MOR. The DOR selectivities displayed by the tested compounds tended to also be observed in the series of alkyl- and amide-type NTI derivatives.

As all the tested compounds **9** showed DOR selectivities, we evaluated the functional activities of **9** for the DOR by [^35^S]GTPγS binding assays according to the previously reported methods [47] (Table 1). Compounds **9a**, **9b**, **9f**, and **9g** showed inverse agonist activities. Among them, compounds **9f** and **9g** showing high efficacies were almost full inverse agonists. The activity of **9f** was comparable to that to SYK-623 (EC_50_ = 0.969 nM, E_max_ = −91.2%) [25]. Interestingly, both compounds possessing the cyclopropyl moiety together with either a C=O or a S=O functionality were inverse agonists, while the NTI-bearing cyclopropylmethyl group, with neither the C=O nor S=O moiety, was a neutral antagonist. Benzylsulfonamide **9d** and phenethylsulfonamide **9e** were DOR agonists. Morphine derivatives with large and small *N*-substituents are known to show antagonist and agonist activities, respectively, while the *N*-phenethyl derivative bearing a larger *N*-substituent was an agonist [30]. Although it is not fully clear why such outcomes were observed, the observations for compounds **9d** and **9e** may result from a similar cause. As no [^35^S]GTPγS binding was measured for compound **9c**, **9c** was a neutral DOR antagonist.

We summarized the binding affinities and functional acitvities of the corresponding alkyl-, amide-, and sulfonamide-type NTI derivatives for the DOR in Table 2. Full agonists, partial agonists, neutral antagonists, partial inverse agonists, and full inverse agonists were categorized based on the following criteria: E_max_ ≥ 80% for full agonists, 80% > E_max_ ≥ 10% for partial agonists, 10% > E_max_ > −10% for neutral antagonists, −10% ≥ E_max_ > −80% for partial inverse agonists, and −80% ≥ E_max_ for full inverse agonists. In the case that the substituent R was methyl, trifluoromethyl, phenyl, or cyclopropyl group, NTI derivatives were neutral antagonsts or inverse agonists. However, no clear trend dependent on the moiety X was observed. On the other hand, the derivatives bearing benzyl or phenethyl substituent as the R group exhibited agonist activities regardless of the moiety X. Although all the derivatives with the phenyl, benzyl, or phenethyl substituent as the R group possessed the phenyl ring in their *N*-substituents, regardless of the moiety X, the derivatives with benzyl-X- or phenethyl-X- group showed positive intrinsic activities whereas *N*-(phenyl-X)- derivatives displayed no positive intrinsic activities. It is not clear why such results were obtained, but the length between the phenyl ring and morphinan core structure may play a key role. When the substituent R is a vinyl group, the X moiety seemed to control the functional activities: an alkyl-type derivative (X = CH_2_) was an agonist while amide-type (X = CO) or sulfonamide-type (X = SO_2_) derivatives were inverse agonists. The outcomes shown in Table 2 critically suggest that the N-substituent (R-X- group) would affect the functional activity. This observation is consistent with the idea that binding of these different structures would selectively alter the conformation of the DOR to induce an agonistic, inverse agonistic activity, or no activity at all. However, it is not yet known which functional group or which interaction would control the conformational changes of the receptor based on the classical structure-activity relationship study carried out in this research. Further investigations using computational simulation, X-ray crystallographic or cryo-electron microscopic studies of the complex of these ligands with the DOR are expected to reveal key insights.

## 3. Materials and Methods

### 3.1. General Information

All reagents and solvents were obtained from commercial suppliers and were used without further purification. Melting points were determined on a Yanako MP-500P melting point apparatus and were uncorrected. IR spectra were recorded on a JASCO FT/IR-460Plus. NMR spectra were recorded on an Agilent Technologies VXR-400NMR for ^1^H- and ^13^C-NMR. Chemical shifts were reported as δ values (ppm) referenced to tetramethylsilane, acetone-*d*_6_, methanol-*d*_4_, or dimethyl sulfoxide-*d*_6_. MS were obtained on a JMS-AX505HA, JMS-700 MStation, or JMS-100LP instrument by applying an electrospray ionization (ESI) method. Elemental analyses were determined with a Yanako MT-5 and JM10 for carbon, hydrogen, and nitrogen. The progress of the reaction was determined on Merck Silica Gel Art. 5715 (TLC) visualized by exposure to UV light or with sodium phosphomolybdate solution (sodium phosphomolybdate (9.68 g) in distillated water (400 mL), sulfuric acid (20 mL), and 85% phosphoric acid (6 mL)). Column chromatographies were carried out using Fuji Silysia CHROMATOREX^®^ PSQ 60B (60 μm) or Fuji Silysia CHROMATOREX^®^ NH-DM2035 (60 μm). The reactions were performed under an argon atmosphere unless otherwise noted. 

### 3.2. Procedures for the Synthesis All the New Compounds and Their Spectroscopic Data

#### 3.2.1. 17-Cyclopropyl-6,7-didehydro-4,5α-epoxyindolo[2′,3′:6,7]morphinan-3,14β-diyl Diacetate (**3**)

A solution of NTI (220 mg, 0.53 mmol) in acetic anhydride (5 mL) was stirred at 83 °C for 2 h. After cooling to room temperature, the reaction mixture was poured into saturated sodium bicarbonate aqueous solution and extracted with chloroform. The combined organic layers were washed with brine and dried over anhydrous sodium sulfate. After removing the solvent *in vacuo*, the residue was purified by silica gel column chromatography to give the title compound **3** (252 mg, 95%) as a yellow amorphous material; IR (film) cm^−1^: 3375, 1730, 1450, 1367, 1255, 1231, 1190, 1155, 1046, 1016, 744. ^1^H NMR (400 MHz, CDCl_3_): δ 0.05–0.17 (m, 2H), 0.46–0.57 (m, 2H), 0.77–0.88 (m, 1H), 1.78 (dd, *J* = 2.0, 12.2 Hz, 1H), 1.96 (s, 3H), 2.24 (s, 3 H), 2.25–2.32 (m, 1H), 2.28 (dd, *J* = 6.9, 12.5 Hz, 1H), 2.42 (dd, *J* = 6.1, 12.5 Hz, 1H), 2.53 (ddd, *J* = 5.3, 12.3, 12.3 Hz, 1H), 2.55 (dd, *J* = 1.3, 16.9 Hz, 1H), 2.72-2.80 (m, 2H), 3.18 (d, *J* = 18.8 Hz, 1H), 3.82 (d, *J* = 16.9 Hz, 1H), 4.70 (d, *J* = 6.2 Hz, 1H), 5.69 (s, 1H), 6.64 (d, *J* = 8.2 Hz, 1H), 6.76 (d, *J* = 8.2 Hz, 1H), 7.03−7.08 (m, 1H), 7.15–7.21 (m, 1H), 7.34 (br d, *J* = 8.2 Hz, 1H), 7.42 (br d, *J* = 7.8 Hz, 1H), 8.09 (s, 1H). ^13^C NMR (100 MHz, CDCl_3_): δ 3.7, 3.8, 9.5, 20.5, 22.3, 24.0, 24.5, 30.9, 43.7, 48.2, 55.7, 59.5, 83.8, 85.7, 110.9, 111.3, 118.7, 119.1, 119.4, 122.4, 122.9, 126.6, 128.6, 130.8, 132.0, 132.8, 137.0, 146.9, 168.5, 170.6. HR-MS (ESI): Calcd for C_30_H_31_N_2_O_5_ [M + H]^+^: 499.2233. Found: 499.2246.

#### 3.2.2. 6,7-Didehydro-4,5α-epoxyindolo[2′,3′:6,7]morphinan-3,14β-diol (nor-NTI) (**5**)

To a solution of compound **3** (1.6 g, 3.22 mmol) in 1,1,2,2-tetrachloroethane (10 mL) were added potassium carbonate (2.2 g, 16.1 mmol) and 2,2,2-trichloroethyl chloroformate (2.22 mL, 16.1 mmol), and the mixture was refluxed for 20 h with stirring. After cooling to room temperature, the reaction mixture was poured into saturated sodium bicarbonate aqueous solution and extracted with chloroform. The combined organic layers were washed with brine and dried over anhydrous sodium sulfate. After removing the solvent in vacuo, the obtained crude product was used in the next reaction without any purification.

To a solution of the crude product in THF (15 mL) was added 4 M sodium hydroxide aqueous solution and the mixture was refluxed for 24 h with stirring. After cooling to room temperature, the reaction mixture was poured into saturated sodium bicarbonate aqueous solution and adjusted to pH 7–8 by addition of 2 M hydrochloric acid, then extracted with a mixed solvent (chloroform:ethanol = 3:1). The combined organic layers were washed with brine and dried over anhydrous sodium sulfate. After removing the solvent in vacuo, the residue was purified by silica gel column chromatography to give the title compound **5** (834 mg, 72% from **3**) as a yellow amorphous material; IR (film) cm^−1^: 3292, 1617, 1501, 1457, 1325, 1240, 1164, 1072, 868. ^1^H NMR (400 MHz, methanol-*d*_4_): δ 1.90 (dd, *J* = 3.8, 13.2 Hz, 1H), 2.65 (dd, *J* = 1.2, 16.0 Hz, 1H), 2.66 (ddd, *J* = 5.2, 13.4, 13.4 Hz, 1H), 2.92 (d, *J* = 16.0 Hz, 1H), 2.99 (ddd, *J* = 4.1, 13.2, 13.2 Hz, 1H), 3.12−3.18 (m, 1H), 3.21 (d, *J* = 19.2 Hz, 1H), 3.46 (dd, *J* = 6.8, 19.2 Hz, 1H), 3.80 (d, *J* = 6.8 Hz, 1H), 5.68 (s, 1H), 6.639 (d, *J* = 8.2 Hz, 1H), 6.648 (d, *J* = 8.2 Hz, 1H), 6.97 (ddd, *J* = 0.9, 7.1, 8.0 Hz, 1H), 7.11 (ddd, *J* = 1.1, 7.1, 8.2 Hz, 1H), 7.33 (d, *J* = 8.2 Hz, 1H), 7.38 (d, *J* = 8.0 Hz, 1H), 7.89 (s, 1H), three protons (OH × 2 and NH) were not observed. ^13^C NMR (100 MHz, DMSO-*d*_6_): δ 47.1, 54.3, 56.1, 67.0, 71.7, 72.1, 79.2, 84.3, 109.6, 111.4, 114.6, 118.3, 118.5, 121.8, 125.0, 126.3, 129.4, 130.2, 136.8, 141.9, 143.9, 170.6. HR-MS (ESI): Calcd for C_22_H_21_N_2_O_3_ [M + H]^+^: 361.1552. Found: 361.1563.

#### 3.2.3. Methyl 6,7-Didehydro-4,5α-epoxy-3,14β-dihydorxyindolo[2′,3′:6,7]morphinan-17-ylcarboxylate (**6**)

The hydrolysis of the crude compound, which was obtained by the reaction of compound **3** with 2,2,2-trichloroethyl chloroformate, was carried out using methanol instead of THF as a solvent, provided compound **5** (60%) and the title compound **6** (21%) as off-white amorphous material; IR (film) cm^−1^: 3400, 1673, 1454, 1325, 909, 735. ^1^H NMR (400 MHz, methanol-*d*_4_): δ 1.58-1.68 (m, 1H), 2.39–2.50 (m, 1H), 2.60 (d, *J* = 15.7 Hz, 0.5H), 2.62 (d, *J* = 15.7 Hz, 0.5H), 2.82-2.99 (m, 3H), 3.32 (d, *J* = 18.2 Hz, 0.5H), 3.33 (d, *J* = 18.2 Hz, 0.5H), 3.71 (s, 1.5H), 3.73 (s, 1.5H), 4.00 (dd, *J* = 5.0, 13.7 Hz, 0.5H), 4.06 (dd, *J* = 5.0, 13.7 Hz, 0.5H), 4.59 (d, *J* = 6.5 Hz, 0.5H), 4.65 (d, *J* = 6.4 Hz, 0.5H), 5.59 (s, 1H), 6.54 (d, *J* = 8.1 Hz, 1H), 6.59 (d, *J* = 8.1 Hz, 1H), 6.94 (ddd, *J* = 0.9, 7.1, 7.9 Hz, 1H), 7.07 (ddd, *J* = 1.0, 7.1, 8.1 Hz, 1H), 7.30 (d, *J* = 8.1 Hz, 1H), 7.37 (d, *J* = 7.9 Hz, 1H), 7.87 (s, 1H), two protons (OH × 2) were not observed. ^13^C NMR (100 MHz, methanol-*d*_4_): δ 30.2, 30.4, 33.6, 38.2, 38.5, 48.8, 53.3, 58.0, 58.3, 74.4, 79.5, 85.99, 86.03, 110.8, 110.9, 112.2, 118.7, 119.4, 119.5, 119.8, 120.1, 123.3, 125.3, 128.1, 130.6, 130.7, 131.6, 138.8, 141.3, 144.9, 158.6. HR-MS (ESI): Calcd for C_24_H_22_N_2_NaO_5_ [M + Na]^+^: 441.1426. Found: 441.1412.

#### 3.2.4. General Synthesis of Sulfonamides **8**

To a solution of compound **7** in dichloromethane were added sulfonyl chloride (RSO_2_Cl) or trifluoromethylsulfonyl anhydride (2–4 eq) and triethylamine (2–4 eq), and the mixture was stirred at 0 °C. The progress of the reaction was monitored by TLC analysis. After completion of the reaction, the reaction mixture was poured into saturated sodium bicarbonate aqueous solution and extracted with chloroform. The combined organic layers were washed with brine and dried over anhydrous sodium sulfate. After removing the solvent *in vacuo*, the residue was purified by silica gel column chromatography.

##### 3-((tert-Butyldimethylsilyl)oxy)-6,7-didehydro-4,5α-epoxy-17-(methylsulfonyl)indolo[2′,3′:6,7]morphinan-14β-ol (**8a**)

Yield: 78%, white oil, IR (neat) cm^-1^: 3583, 2927, 1497, 1443, 1323, 1274, 1147, 1044, 955, 906, 842. ^1^H NMR (400 MHz, CDCl_3_): δ 0.02 (s, 3H), 0.04 (s, 3H), 0.89 (s, 9H), 1.82 (dd, *J* = 2.2, 12.8 Hz, 1H), 2.50 (ddd, *J* = 5.4, 12.8, 12.8 Hz, 1H), 2.69 (d, *J* = 15.9 Hz, 1H), 2.91 (d, *J* = 15.9 Hz, 1H), 3.03 (s, 3H), 3.15 (ddd, *J* = 3.5, 13.3, 13.3 Hz, 1H), 3.17 (d, *J* = 18.8 Hz, 1H), 3.39 (dd, *J* = 6.6, 18.8 Hz, 1H), 3.74 (dd, *J* = 5.3, 13.7 Hz, 1H), 4.41 (d, *J* = 6.6 Hz, 1H), 5.58 (s, 1H), 6.56 (d, *J* = 8.2 Hz, 1H), 6.61 (d, *J* = 8.2 Hz, 1H), 7.07 (ddd, *J* = 0.9, 7.1, 7.9 Hz, 1H), 7.19 (ddd, *J* = 1.1, 7.1, 8.1 Hz, 1H), 7.34 (br d, *J* = 8.2 Hz, 1H), 7.41 (br d, *J* = 8.0 Hz, 1H), 8.11 (s, 1H), a proton (OH) was not observed. ^13^C NMR (100 MHz, CDCl_3_): δ −4.8, −4.7, 18.2, 25.5, 29.7, 29.8, 33.9 , 38.9, 45.9, 47.9, 58.4, 73.3, 83.7, 109.7, 111.3, 118.8, 119.3, 119.8, 122.6, 123.4, 125.1, 126.5, 128.8, 129.3, 137.1, 138.9, 146.6. HR-MS (ESI): Calcd for C_29_H_36_N_2_NaO_5_SSi [M + Na]^+^: 575.2012. Found: 575.2029.

##### 3-((tert-Butyldimethylsilyl)oxy)-6,7-didehydro-4,5α-epoxy-17-(1,1,1-trifluoromethylsulfonyl)indolo[2′,3′:6,7]morphinan-14β-ol (**8b**)

Yield: 74%, white amorphous material, IR (film) cm^−1^: 3417, 2930, 2857, 1605, 1498, 1382, 1195, 1115, 1044, 842. ^1^H NMR (400 MHz, CDCl_3_): δ 0.02 (s, 3H), 0.04 (s,3H), 0.88 (s, 9H), 1.75 (br d, *J* = 12.8 Hz, 1H), 2.53–2.81 (m, 3 H), 3.09 (d, *J* = 18.9 Hz, 1H), 3.24 (ddd, *J* = 2.9, 13.5, 13.5 Hz, 1H), 3.40 (dd, *J* = 6.6, 18.9 Hz, 1H), 3.86 (dd, *J* = 4.6, 13.9 Hz, 1H), 4.34 (d, *J* = 6.6 Hz, 1H), 5.57 (s, 1H), 6.57 (d, *J* = 8.2 Hz, 1H), 6.63 (d, *J* = 8.2 Hz, 1H), 7.05–7.10 (m, 1H), 7.20 (ddd, *J* = 1.1, 7.1, 8.2 Hz, 1H), 7.33 (dd, *J* = 0.7, 8.2 Hz, 1H), 7.37 (br d, *J* = 7.8 Hz, 1H), 8.15 (s, 1H), a proton (OH) was not observed. ^13^C NMR (100 MHz, CDCl_3_): δ −4.8, −4.7, 18.2, 25.5, 29.68, 29.71, 29.9, 40.5, 47.5, 60.0, 72.4, 83.6, 109.7, 111.3, 118.8, 119.2, 119.8, 122.8, 123.3, 124.0, 126.4, 128.6, 129.3, 137.1, 139.2, 146.6, the carbon binding to fluorines was not determined. HR-MS (ESI): Calcd for C_29_H_34_F_3_N_2_O_5_SSi [M + H]^+^: 607.1910. Found: 607.1898.

##### 3-((tert-Butyldimethylsilyl)oxy)-6,7-didehydro-4,5α-epoxy-17-(phenylsulfonyl)indolo[2′,3′:6,7]morphinan-14β-ol (**8c**)

Yield: 90%, white oil, IR (neat) cm^−1^: 3399, 3059, 3023, 2928, 2857, 1714, 1631, 1604, 1496, 1455. ^1^H NMR (400 MHz, CDCl_3_): δ −0.02 (s, 3H), 0.01 (s, 3H), 0.87 (s, 9H), 1.82 (dd, *J* = 1.0, 13.0 Hz, 1H), 2.45 (ddd, *J* = 5.4, 12.8, 12.8 Hz, 1H), 2.606 (d, *J* = 15.8 Hz, 1H), 2.609 (d, *J* = 18.8 Hz, 1H), 2.82 (s, 1H), 2.922 (d, *J* = 15.8 Hz, 1H), 2.924 (ddd, *J* = 3.6, 12.6, 12.6 Hz, 1H), 3.10 (dd, *J* = 6.6, 18.8 Hz, 1H), 3.80 (dd, *J* = 5.2, 12.7 Hz, 1H), 4.42 (d, *J* = 6.6 Hz, 1H), 5.58 (s, 1H), 6.36 (d, *J* = 8.2 Hz, 1H), 6.52 (d, *J* = 8.2 Hz, 1H), 7.04 (ddd, *J* = 0.9, 7.0, 7.9 Hz, 1H), 7.16 (ddd, *J* = 1.1, 7.0, 8.2 Hz, 1H), 7.30 (br d, *J* = 8.2 Hz, 1H), 7.39(br d, *J* = 7.8 Hz, 1H), 7.53–7.60 (m, 2H), 7.61–7.66 (m, 1H), 7.87–7.92 (m, 2H), 8.06 (s, 1H). ^13^C NMR (100 MHz, CDCl_3_): δ −4.8, −4.7, 18.2, 25.5, 29.1, 30.1, 30.5, 38.7, 47.7, 58.7, 72.8, 84.1, 110.7, 111.1, 118.9, 119.5, 122.4, 123.1, 124.7, 126.5, 127.2, 128.4, 129.4, 129.8, 132.9, 137.1, 138.9, 139.8, 146.5, two signals in the aromatic region would be overlapped. HR-MS (ESI): Calcd for C_34_H_39_N_2_O_5_SSi [M + H]^+^: 615.2349. Found: 615.2359.

##### 17-(Benzylsulfonyl)-3-((tert-butyldimethylsilyl)oxy)-6,7-didehydro-4,5α-epoxyindolo[2′,3′:6,7]morphinan-14β-ol (**8d**)

Yield: 98%, white amorphous material, IR (film) cm^−1^: 3397, 3061, 2953, 2928, 2856, 1497, 1324, 1274, 1043, 852. ^1^H NMR (400 MHz, CDCl_3_): δ 0.00 (s, 3H), 0.02 (s, 3H), 0.88 (s, 9H), 1.68 (dd, *J* = 2.0, 12.9 Hz, 1H), 2.33 (ddd, *J* = 5.4, 12.9, 12.9 Hz, 1H), 2.61 (d, *J* = 15.6 Hz, 1H), 2.84 (d, *J* = 15.6 Hz, 1H), 2.94 (d, *J* = 19.0 Hz, 1H), 3.02 (ddd, *J* = 3.5, 13.2, 13.2 Hz, 1H), 3.24 (dd, *J* = 6.6, 19.0 Hz, 1H), 3.38 (dd, *J* = 5.3, 13.6 Hz, 1H), 4.23 (d, *J* = 6.6 Hz, 1H), 4.32 (d, *J* = 14.0 Hz, 1H), 4.42 (d, *J* = 14.0 Hz, 1H), 5.54 (s, 1H), 6.49 (d, *J* = 8.2 Hz, 1H), 6.57 (d, *J* = 8.2 Hz, 1H), 7.04 (ddd, *J* = 0.9, 7.1, 7.9 Hz, 1H), 7.17 (ddd, *J* = 1.0, 7.1, 8.2 Hz, 1H), 7.32 (d, *J* = 8.2 Hz, 1H), 7.38 (d, *J* = 7.9 Hz, 1H), 7.37–7.50 (m, 5H), 8.07 (s,1H), a proton (OH) was not observed. ^13^C NMR (100 MHz, CDCl_3_): δ −4.8, −4.7, 18.2, 25.5, 29.5, 29.6, 33.2, 39.5, 47.7, 58.7, 58.9, 73.0, 83.9, 110.4, 111.2, 118.8, 119.0, 119.6, 122.4, 123.1, 125.1, 126.6, 128.6, 128.8, 128.9, 129.4, 129.7, 130.6, 137.1, 138.8, 146.4. HR-MS (ESI): Calcd for C_35_H_41_N_2_O_5_SSi [M + H]^+^: 629.2505. Found: 629.2492.

##### 3-((tert-Butyldimethylsilyl)oxy)-6,7-didehydro-4,5α-epoxy-17-((2-phenyethyl)sulfonyl)indolo[2′,3′:6,7]morphinan-14β-ol (**8e**)

Yield: 46%, pale yellow oil, IR (neat) cm^−1^: 2928, 1497, 1443, 1275, 1147, 1044, 907, 853, 741, 423. ^1^H NMR (400 MHz, CDCl_3_): δ 0.01 (s, 3H), 0.04 (s, 3H), 0.88 (s, 9H), 1.78 (br d, *J* = 12.7 Hz, 1H), 2.47 (ddd, *J* = 5.3, 12.7, 12.7 Hz, 1 H), 2.68 (d, *J* = 15.8 Hz, 1H), 2.87 (d, *J* = 15.8 Hz, 1H), 3.11-3.23 (m , 4H), 3.32-3.43 (m, 3 H), 3.75 (dd, *J* = 5.1, 13.6 Hz, 1H), 4.33 (d, *J* = 6.4 Hz, 1H), 5.55 (s, 1H), 6.54 (d, *J* = 8.2 Hz, 1H), 6.60 (d, *J* = 8.2 Hz, 1H), 7.04–7.09 (m, 1H), 7.16-7.35 (m, 7H), 7.40 (br d, *J* = 7.9 Hz, 1H), 8.13 (br s, 1H), a proton (OH) was not observed. ^13^C NMR (100 MHz, CDCl_3_): δ −4.8, −4.7, 18.2, 25.5, 28.4, 29.7, 29.8, 33.6, 38.9, 47.9, 54.4, 58.4, 73.1, 83.9, 110.1, 111.2, 118.8, 119.1, 119.7, 122.5, 123.2, 125.1, 126.6, 126.8, 128.4, 128.7, 128.8, 129.6, 137.1, 138.2, 138.8, 146.5. HR-MS (ESI): Calcd for C_36_H_42_N_2_NaO_5_SSi [M + Na]^+^: 665.2481. Found: 665.2468.

##### 3-((tert-Butyldimethylsilyl)oxy)-17-(cyclopropylsulfonyl)-6,7-didehydro-4,5α-epoxyindolo[2′,3′:6,7]morphinan-14β-ol (**8f**)

Yield: 98%, white amorphous material, IR (film) cm^−1^: 3397, 2928, 1604, 1497, 1444, 1325, 1274, 1147, 1046, 937, 906, 852. ^1^H NMR (400 MHz, CDCl_3_): δ 0.01 (s, 3H), 0.04 (s, 3H), 0.89 (s, 9H), 1.00–1.10 (m, 2H), 1.20–1.28 (m, 2H), 1.83 (br d, *J* = 11.2 Hz, 1H), 2.44–2.55 (m, 2H), 2.67 (d, *J* = 15.8 Hz, 1H), 2.83 (s, 1H), 2.93 (d, *J* = 15.8 Hz, 1H), 3.13 (ddd, *J* = 3.4, 12.9, 12.9 Hz, 1H), 3.32 (d, *J* = 18.7 Hz, 1H), 3.37 (dd, *J* = 6.2, 18.7 Hz, 1H), 3.73 (dd, *J* = 5.0, 13.0 Hz, 1H), 4.35 (d, *J* = 6.2 Hz, 1H), 5.60 (s, 1H), 6.55 (d, *J* = 8.2 Hz, 1H), 6.60 (d, *J* = 8.2 Hz, 1H), 7.05 (br dd, *J* = 7.5, 7.5 Hz, 1H), 7.18 (br dd, *J* = 7.6, 7.6 Hz, 1H), 7.32 (d, *J* = 8.2 Hz, 1H), 7.40 (d, *J* = 7.9 Hz, 1H), 8.09 (s, 1H). ^13^C NMR (100 MHz, CDCl_3_): δ −4.8, −4.7, 5.3, 5.7, 18.2, 25.5, 29.2, 29.7, 30.0, 32.4, 38.9, 47.7, 58.8, 72.8, 84.1, 110.5, 111.2, 118.8, 118.9, 119.5, 122.4, 123.0, 124.9, 126.5, 128.6, 129.9, 137.1, 138.9, 146.5. HR-MS (ESI): Calcd for C_31_H_39_N_2_O_5_SSi [M + H]^+^: 579.2349. Found: 579.2367.

#### 3.2.5. 3-((tert-Butyldimethylsilyl)oxy)-6,7-didehydro-4,5α-epoxy-17-(ethenylsulfonyl)indolo[2′,3′:6,7]morphinan-14β-ol (**8g**)

To a solution of compound **7** (29.9 mg, 0.063 mmol) in dichloromethane (3 mL) were added 2-chloroethanesulfonyl chloride (22.0 μL, 0.20 mmol) and 0.5 M sodium hydroxide aqueous solution (15 μL), and the mixture was stirred at 0 °C for 20 h. The reaction mixture was poured into saturated sodium bicarbonate aqueous solution and extracted with chloroform. The combined organic layers were washed with brine and dried over anhydrous sodium sulfate. After removing the solvent *in vacuo*, the residue was purified by silica gel column chromatography to give the title compound **8f** (19.8 mg, 56%) as a white amorphous material; IR (film) cm^−1^: 3397, 2928, 2857, 1497, 1444, 1325, 1148, 1043, 852, 744. ^1^H NMR (400 MHz, CDCl_3_): δ 0.01 (s, 3H), 0.04 (s, 3H), 0.88 (s, 9H), 1.80 (dd, *J* = 2.1, 13.0 Hz, 1H), 2.46 (ddd, *J* = 5.4, 12.8, 12.8 Hz, 1H), 2.67 (dd, *J* = 0.9, 15.9 Hz, 1H), 2.91 (d, *J* = 15.9 Hz, 1H), 3.06 (ddd, *J* = 3.5, 13.1, 13.1 Hz, 1H), 3.17 (d, *J* = 18.8 Hz, 1H), 3.34 (dd, *J* = 6.5, 18.8 Hz, 1H), 3.63 (dd, *J* = 5.3, 13.3 Hz, 1H), 4.37 (d, *J* = 6.5 Hz, 1H), 5.58 (s, 1H), 5.99 (d, *J* = 10.0 Hz, 1H), 6.31 (d, *J* = 16.6 Hz, 1H), 6.54 (d, *J* = 8.2 Hz, 1H), 6.60 (d, *J* = 8.2 Hz, 1H), 6.65 (dd, *J* = 10.0, 16.6 Hz, 1H), 7.06 (ddd, *J* = 0.8, 7.0, 7.9 Hz, 1H), 7.18 (ddd, *J* = 1.1, 7.0, 8.2 Hz, 1H), 7.33 (br d, *J* = 8.2 Hz, 1H), 7.40 (br d, *J* = 7.8 Hz, 1H), 8.10 (s, 1H), a proton (OH) was not observed. ^13^C NMR (100 MHz, CDCl_3_): δ −4.8, −4.7, 18.2, 25.5, 29.4, 29.6, 32.6, 38.3, 47.8, 58.4, 72.9, 84.0, 110.4, 111.2, 118.9, 119.1, 119.6, 122.5, 123.2, 124.9, 126.6, 127.1, 128.6, 129.7, 135.7, 137.1, 138.9, 146.5. HR-MS (ESI): Calcd for C_30_H_37_N_2_O_5_SSi [M + H]^+^: 565.2192. Found: 565.2195.

#### 3.2.6. General Synthesis of Test Compounds **9**

To a solution of compound **8** in THF was added 1.0 M solution of tetrabutylammonium fluoride in THF (excess), and the mixture was stirred at room temperature. The progress of the reaction was monitored by TLC analysis. After completion of the reaction, the reaction mixture was poured into saturated sodium bicarbonate aqueous solution and extracted with a mixed solvent (chloroform: ethanol = 3:1). The combined organic layers were washed with brine and dried over anhydrous sodium sulfate. After removing the solvent *in vacuo*, the residue was purified by silica gel column chromatography.

##### 6,7-Didehydro-4,5α-epoxy-17-(methylsulfonyl)indolo[2′,3′:6,7]morphinan-3,14β-diol (**9a**)

Yield: 87%, white amorphous material, IR (film) cm^−1^: 3855, 3823, 3364, 1691, 1314, 1149, 899, 803, 752, 467. ^1^H NMR (400 MHz, CDCl_3_): δ 1.80 (dd, *J* = 2.2, 12.9 Hz, 1H), 2.49 (ddd, *J* = 5.4, 12.8, 12.8 Hz, 1H), 2.61 (dd, *J* = 0.9, 15.9 Hz, 1H), 2.67 (s, 1H), 2.91 (d, *J* = 15.9 Hz, 1H), 3.22 (s, 3H), 3.14 (ddd, *J* = 3.5, 13.3, 13.3 Hz, 1H), 3.16 (d, *J* = 18.7 Hz, 1H), 3.36 (dd, *J* = 6.5, 18.7 Hz, 1H), 3.74 (dd, *J* = 5.3, 13.6 Hz, 1H), 4.40 (d, *J* = 6.5 Hz, 1H), 5.02 (br s, 1H), 5.61 (s, 1H ), 6.58 (d, *J* = 8.2 Hz, 1H), 6.65 (d, *J* = 8.2 Hz, 1H), 7.07 (ddd, *J* = 0.9, 7.0, 7.9 Hz, 1H), 7.18 (ddd, *J* = 1.1, 7.0, 8.2 Hz, 1H), 7.28 (d, *J* = 8.2 Hz, 1H), 7.40 (d, *J* = 7.9 Hz, 1H), 8.29 (s, 1H). ^13^C NMR (100 MHz, CDCl_3_): δ 29.5, 30.9, 32.9, 38.6, 40.0, 48.0, 58.4, 73.2, 85.0, 110.5, 111.4, 117.8, 118.9, 119.6, 119.8, 123.4, 124.1, 126.5, 128.4, 129.2, 137.1, 139.3, 143.0. HR-MS (ESI): Calcd for C_23_H_23_N_2_O_5_S [M + H]^+^: 439.1328. Found: 439.1336. Anal. Calcd for C_23_H_22_N_2_O_5_S·1.1EtOH: C, 61.87; H, 5.89; N, 5.73. Found C, 61.47; H, 5.53; N, 5.36.

##### 6,7-Didehydro-4,5α-epoxy-17-(1,1,1-trifluoromethylsulfonyl)indolo[2′,3′:6,7]morphinan-3,14β-diol (**9b**)

Yield: 74%, white amorphous material, IR (film) cm^−1^: 3410, 2924, 1707, 1638, 1618, 1508, 1456, 1380, 1195, 1113. ^1^H NMR (400 MHz, CDCl_3_): δ 1.77 (br d, *J* = 12.6 Hz, 1H), 2.63 (ddd, *J* = 5.3, 12.8, 12.8 Hz, 1H), 2.65 (d, *J* = 15.9 Hz, 1H), 2.82 (d, *J* = 15.9 Hz, 1H), 3.16 (d, *J* = 18.9 Hz, 1H), 3.25 (ddd, *J* = 2.8, 13.3, 13.3 Hz, 1H), 3.44 (dd, *J* = 6.7, 18.9 Hz, 1H), 3.88 (dd, *J* = 4.9, 13.8 Hz, 1H), 4.38 (d, *J* = 6.7 Hz, 1H), 4.92 (br s, 1H), 5.65 (s, 1H), 6.61 (d, *J* = 8.2 Hz, 1H), 6.68 (d, *J* = 8.2 Hz, 1H), 7.08 (dd, *J* = 7.4, 7.4 Hz, 1H), 7.13-7.28 (m, 1H), 7.32 (br d, *J* = 8.2 Hz, 1H), 7.37 (br d, *J* = 7.9 Hz, 1H), 8.25 (s, 1H), a proton (OH) was not observed. ^13^C NMR (100 MHz, CDCl_3_): δ 29.7, 37.7, 40.5, 47.6, 59.9, 72.6, 85.0, 110.0, 111.5, 118.1, 118.9, 119.8, 119.9, 121.5, 123.5, 126.3, 128.1, 128.8, 137.1, 139.4, 142.9, a carbon in an aliphatic region was not observed, and the carbon binding to fluoride was not determined. HR-MS (ESI): Calcd for C_23_H_20_F_3_N_2_O_5_S [M + H]^+^: 493.1045. Found: 493.1058. Anal. Calcd for C_23_H_19_F_3_N_2_O_5_S·MeOH: C, 54.85; H, 4.60; N, 5.33. Found C, 54.90; H, 4.73; N, 5.12.

##### 6,7-Didehydro-4,5α-epoxy-17-(phenylsulfonyl)indolo[2′,3′:6,7]morphinan-3,14β-diol (**9c**)

Yield: 84%, white amorphous material, IR (film) cm^−1^: 3395, 2961, 2874, 1719, 1638, 1457, 1323, 1273, 1159, 730. ^1^H NMR (400 MHz, aceton-*d*_6_): δ 1.69-1.76 (m, 1H), 2.59 (ddd, *J* = 5.4, 12.7, 12.7 Hz, 1H), 2.62 (dd, *J* = 1.0, 15.9 Hz, 1H), 2.67 (d, *J* = 18.6 Hz, 1H), 2.92 (ddd, *J* = 3.5, 12.8, 12.8 Hz, 1H), 2.94 (d, *J* = 15.9 Hz, 1H), 3.21 (dd, *J* = 6.4, 18.6 Hz, 1H), 3.73 (dd, *J* = 5.2, 12.8 Hz, 1H), 4.14 (s, 1H), 4.47 (d, *J* = 6.4 Hz, 1H), 5.59 (s, 1H), 6.39 (d, *J* = 8.1 Hz, 1H), 6.56 (d, *J* = 8.1 Hz, 1H), 6.96 (ddd, *J* = 0.9, 7.1, 8.0 Hz, 1H), 7.10 (ddd, *J* = 1.1, 7.1, 8.1 Hz, 1H), 7.35–7.39 (m, 2H), 7.59–7.67 (m, 3H), 7.84 (br s, 1H), 7.93-7.99 (m, 2H), 10.2 (br s, 1H). ^13^C NMR (100 MHz, DMSO-*d*_6_): δ 28.8, 29.0, 31.3, 38.5, 47.0, 58.2, 71.8, 71.9, 83.8, 109.5, 111.4, 117.3, 118.38, 118.44, 121.9, 123.1, 126.4, 126.9, 129.3, 129.5, 130.0, 132.6, 136.8, 140.3, 140.5, 143.2, two signals in the aromatic region would be overlapped. HR-MS (ESI): Calcd for C_28_H_25_N_2_O_5_S [M + H]+: 501.1484. Found: 501.1474. Anal. Calcd for C_28_H_24_N_2_O_5_S·H_2_O: C, 64.85; H, 5.05; N, 5.40. Found C, 64.92; H, 4.83; N, 5.31.

##### 17-(Benzylsulfonyl)-6,7-didehydro-4,5α-epoxyindolo[2′,3′:6,7]morphinan-3,14β-diol (**9d**)

Yield: 84%, white amorphous material, IR (film) cm^−1^: 3405, 1701, 1638, 1499, 1457, 1321, 1115, 1040, 862, 742, 700. ^1^H NMR (400 MHz, acetone-*d*_6_): δ 1.62 (dd, *J* = 2.3, 12.7 Hz, 1H), 2.58 (ddd, *J* = 5.5, 12.8, 12.8 Hz, 1H), 2.67 (dd, *J* = 0.9, 15.9 Hz, 1H), 2.95 (d, *J* = 15.9 Hz, 1H), 2.88-3.08 (m, 1H), 3.01 (d, *J* = 18.7 Hz, 1H), 3.38 (dd, *J* = 6.2, 18.7 Hz, 1H), 3.56 (dd, *J* = 5.3, 13.7 Hz, 1H), 4.28 (d, *J* = 6.2 Hz, 1H), 4.39 (s, 1H), 4.47 (d, *J* = 13.7 Hz, 1H), 4.56 (d, *J* = 13.7 Hz, 1H), 5.60 (s, 1H), 6.52 (d, *J* = 8.0 Hz, 1H), 6.62 (d, *J* = 8.0 Hz, 1H), 6.85–7.02 (m, 1H), 7.12 (ddd, *J* = 1.0, 7.0, 8.2 Hz, 1H), 7.24–7.42 (m, 5H), 7.53–7.59 (m, 2H), 7.87 (br s, 1H), 10.23 (s, 1H). ^13^C NMR (100 MHz, CDCl_3_): δ 29.4, 29.6, 32.9, 39.4, 47.8, 58.6, 59.0, 73.2, 85.0, 110.5, 111.5, 117.8 118.8, 119.5, 119.6, 123.1, 124.1, 126.4, 128.3, 128.9, 129.16, 129.21, 130.6, 137.1, 139.2, 142.8, 152.8. HR-MS (ESI): Calcd for C_29_H_27_O_5_S [M + H]^+^: 515.1641. Found: 515.1632. Anal. Calcd for C_29_H_26_N_2_O_5_S·2H_2_O·EtOH: C, 62.40; H, 6.08; N, 6.69. Found C, 62.12; H, 5.75; N, 4.43.

##### 6,7-Didehydro-4,5α-epoxy-17-((2-phenylethyl)sulfonyl)indolo[2′,3′:6,7]morphinan-3,14β-diol (**9e**)

Yield: 79%, white amorphous material, IR (film) cm^−1^: 3407, 2923, 1504, 1455, 1319, 1146, 1041, 903, 736. ^1^H NMR (400 MHz, CDCl_3_): δ 1.78 (br d, *J* = 12.0 Hz, 1H), 2.47 (ddd, *J* = 5.2, 12.7, 12.7 Hz, 1H), 2.63 (d, *J* = 15.7 Hz, 1H), 2.64 (s, 1H), 2.87 (d, *J* = 15.7 Hz, 1H), 3.09–3.24 (m, 4H), 3.30–3.45 (m, 3H), 3. 75 (dd, *J* = 4.9, 13.8 Hz, 1H), 4.34 (d, *J* = 6.4 Hz, 1H), 5.14 (br s, 1H), 5.59 (s, 1H), 6.57 (d, *J* = 8.2 Hz, 1H), 6.64 (d, *J* = 8.2 Hz, 1H), 7.06 (dd, *J* = 7.4, 7.4 Hz, 1 H), 7.17 (dd, *J* = 7.5, 7.5 Hz, 1H), 7.19–7.33 (m, 6H), 7.38 (d, *J* = 7.8 Hz, 1H), 8.31 (s, 1H) 1H). ^13^C NMR (100 MHz, CDCl_3_): δ 20.1, 29.6, 29.9, 33.4, 38.9, 48.1, 54.5, 58.4, 73.2, 84.9, 110.4, 111.5, 117.8, 118.9, 119.6, 119.8, 123.4, 124.2, 126.5, 126.9, 128.42, 128.45, 128.9, 129.2, 137.2, 138.2, 139.3, 145.0. HR-MS (ESI): Calcd for C_30_H_28_N_2_NaO_5_S [M + Na]^+^: 551.1617. Found: 551.1623. Anal. Calcd for C_30_H_28_N_2_O_5_S·3.5H_2_O·0.5CHCl_3_: C, 56.24; H, 5.49; N, 4.30. Found C, 56.36; H, 5.71; N, 3.99.

##### 17-(Cyclopropylsulfonyl)-6,7-didehydro-4,5α-epoxyindolo[2′,3′:6,7]morphinan-3,14β-diol (**9f**)

Yield: 96%, white amorphous material, IR (film) cm^−1^: 3400, 2922, 1719, 1638, 1508, 1457, 1322, 1145, 1044, 899, 726, 499. ^1^H N MR (400 MHz, aceton-*d*_6_): δ 0.95–1.14 (m, 4H), 1.73 (dd, *J* = 2.0, 12.7 Hz, 1H), 2.659 (ddd, *J* = 5.4, 12.7, 12.7 Hz, 1H), 2.665 (d, *J* = 15.6 Hz, 1H), 2.74–2.83 (m, 1H), 2.94 (d, *J* = 15.6 Hz, 1H), 3.11 (ddd, *J* = 3.5, 13.1, 13.1 Hz, 1H), 3.25 (d, *J* = 18.7 Hz, 1H), 3.43 (dd, *J* = 6.7, 18.7 Hz, 1H), 3.70 (dd, *J* = 5.4, 13.3 Hz, 1H), 4.23 (s, 1H), 4.35 (d, *J* = 6.7 Hz, 1H), 5.61 (s, 1H), 6.57 (d, *J* = 8.1 Hz, 1H), 6.63 (d, *J* = 8.1 Hz, 1H), 6.97 (ddd, *J* = 0.9, 7.1, 7.9 Hz, 1H), 7.11 (ddd, *J* = 1.0, 7.1, 8.2 Hz, 1H), 7.381 (br d, *J* = 8.2 Hz, 1H), 7.388 (br d, *J* = 8.3 Hz, 1H), 10.2 (s, 1H), a proton (OH) was not observed. ^13^C NMR (100 MHz, CDCl_3_): δ 5.3, 5.7, 29.2, 29.8, 30.0, 32.3, 38.9, 47.8, 58.8, 73.1, 85.2, 110.7, 111.4, 117.8, 118.9, 119.5, 119.6, 123.2, 123.9, 126.4, 128.2, 129.5, 137.1, 139.3, 142.9. HR-MS (ESI): Calcd for C_25_H_24_N_2_NaO_5_S [M + Na]^+^: 487.1304. Found: 487.1283. Anal. Calcd for C_25_H_24_N_2_O_5_S·1.5H_2_O·0.2CHCl_3_: C, 58.72; H, 5.32; N, 5.43. Found C, 58.68; H, 5.16; N, 5.08.

##### 6,7-Didehydro-4,5α-epoxy-17-(ethenylsulfonyl)indolo[2′,3′:6,7]morphinan-3,14β-diol (**9g**)

Yield: 71%, white amorphous material, IR (film) cm^−1^: 3400, 1455, 1322, 1146, 903, 738. ^1^H NMR (400 MHz, CDCl_3_): δ 1.79 (dd, *J* = 2.1, 12.8 Hz, 1H), 2.46 (ddd, *J* = 5.3, 12.8, 12.8 Hz, 1H), 2.63 (d, *J* = 15.8 Hz, 1H), 2.78 (s, 1H), 2.90 (d, *J* = 15.8 Hz, 1H), 3.05 (ddd, *J* = 3.5, 13.1, 13.1 Hz, 1H), 3.14 (d, *J* = 18.7 Hz, 1H), 3.32 (dd, *J* = 6.5, 18.7 Hz, 1H), 3.63 (dd, *J* = 5.3, 13.3 Hz, 1H), 4.37 (d, *J* = 6.5 Hz, 1H), 5.12 (br s, 1H), 5.61 (s, 1H), 5.99 (d, *J* = 9.8 Hz, 1H), 6.32 (d, *J* = 16.4 Hz, 1H), 6.65 (d, *J* = 8.2 Hz, 1H), 6.639 (d, *J* = 8.2 Hz, 1H), 6.641 (dd, *J* = 9.8, 16.4 Hz, 1H), 7.06 (dd, *J* = 7.4, 7.4 Hz, 1H), 7.17 (dd, *J* = 7.5, 7.5 Hz, 1H), 7.25–7.29 (m, 1H), 7.39 (d, *J* = 7.9 Hz, 1H), 8.30 (s, 1H). ^13^C NMR (100 MHz, CDCl_3_): δ 29.4, 29.6, 32.5, 38.4, 47.9, 58.4, 73.1, 85.1, 110.7, 111.4, 117.8, 118.9, 119.5, 119.8, 123.4, 124.0, 126.5, 127.2, 128.3, 129.3, 135.6, 137.1, 139.3, 142.9. HR-MS (ESI): Calcd for C_24_H_22_N_2_NaO_5_S [M + Na]_+_: 473.1147. Found: 473.1138. Anal. Calcd for C_24_H_22_N_2_O_5_S·EtOH·0.3CHCl_3_: C, 59.33; H, 5.36; N, 5.26. Found C, 59.17; H, 5.22; N, 5.15.

### 3.3. Calculation of the Most Stable Conformations of Alkyl-, Amide-, and Sulfonamide-Type Piperidines

The initial geometries of the molecular models for density functional theory (DFT) calculations were prepared using Schrödinger suites (Schrödinger, LLC) [48,49,50,51,52,53,54]. Structural optimizations were performed for the prepared molecular models with ωB97XD/6-31G(d,p) level of theory, respectively. Their vibration frequencies were calculated at the same level to confirm their stationary structure. All of the DFT calculations were performed using the Gaussian09 program [55].

### 3.4. Bioassays

#### 3.4.1. Membrane Preparation

The human MOR, DOR, or KOR recombinant CHO cell pellets were resuspended in 50 mM Tris–HCl buffer containing 5 mM MgCl_2_, 1 mM ethylene glycol bis(2-aminoethyl ether)-*N*,*N*,*N*,*N*-tetraacetic acid (EGTA), pH 7.4. The cell suspensions were disrupted by use of a glassteflon homogeniser and centrifuged at 48,000× *g* for 15 min. The supernatant was discarded and the pellets were resuspended in buffer at a concentration of 5 mg protein/mL and stored at −80 °C until further use.

#### 3.4.2. Competitive Binding Assays

Human MOR, DOR, or KOR recombinant CHO cell membranes were incubated for 2 h at 25 °C in 0.25 mL of the buffer containing with various concentrations of the tested compound, 2 nM [^3^H] DAMGO, [^3^H] DPDPE, or [^3^H] U69,593 (PerkinElmer, Inc., MA, USA), respectively. The incubation was terminated by collecting membranes on Filtermat B filter (PerkinElmer, Inc.) using a FilterMate™ harvester (PerkinElmer, Inc.). The filters were then washed three times with 50 mM Tris–HCl buffer, pH 7.4. Then, MeltiLex scintillant (PerkinElmer, Inc.) was melted onto the dried filters. Radioactivity was determined by a MicroBeta scintillation counter (PerkinElmer, Inc.). Nonspecific binding was measured in the presence of 10 μM unlabeled DAMGO, DPDPE, or U-69,593 (PerkinElmer, Inc.). *K*_i_ and 95% CI values were calculated by Prism software (version 5.0).

#### 3.4.3. [^35^S]GTPγS Binding Assays

Human MOR, DOR, or KOR recombinant CHO cell membranes were incubated for 2 h at 25 °C in 0.25 mL of 50 mM Tris–HCl buffer containing 5 mM MgCl_2_, 1 mM EGTA, 100 mM NaCl, pH 7.4 with various concentrations of the tested compound, 30 μM guanosine 5-diphosphate (GDP) and 0.1 nM [^35^S]GTPγS (PerkinElmer, Inc.). The incubation was terminated by collecting membranes on Filtermat B filter (PerkinElmer, Inc.) using a FilterMate™ harvester (Perkin Elmer, Inc.). The filters were then washed three times with 50 mM Tris–HCl buffer, pH 7.4. Then, MeltiLex scintillant (PerkinElmer, Inc.) was melted onto the dried filters. Radioactivity was determined by a MicroBeta scintillation counter (PerkinElmer, Inc.). EC_50_, E_max_, and 95% CI values were calculated by Prism software (version 5.0). Nonspecific binding was measured in the presence of 10 μM unlabeled GTPγS (PerkinElmer, Inc.). DPDPE and ICI-174,864 (PerkinElmer, Inc.) were used as the standard DOR full agonist and full inverse agonist, respectively.

## 4. Conclusions

We synthesized sulfonamide-type NTI derivatives to compare their functional activities for the DOR with those of the corresponding alkyl- and amide-type NTI derivatives. Among them, cyclopropylsulfonamide **9f** (SYK-839) was the most potent full inverse agonist. Its potency and efficacy were comparable to those of SYK-623, a previously reported amide-type full inverse agonist. On the other hand, phenethylsulfonamide **9e** (SYK-901) showed full agonist activity with moderate potency. Not only the sulfonamide-type NTI derivatives but also the alkyl- and amide-type derivatives, which induced activities for the DOR from full inverse agonist to full agonist, are expected to be useful compounds for investigation of the relationship among the interactions of ligands with the DOR, conformational changes of the DOR, and the induced functional activities.

## Figures and Tables

**Table 1:**
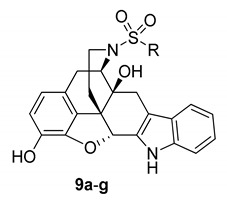
Binding affinities of compounds **9** for the opioid receptors ^a^ and functional activities of compounds **9** for the DOR ^b^.

Compd	R	Binding Affinity, nM (95% CI)			Functional Activity for DOR	
		*K*_i_ (DOR)	*K*_i_ (MOR)	*K*_i_ (KOR)	EC_50_, nM (95% CI)	E_max_, % (95% CI)
DPDPE ^c^	―	NT ^d^	NT ^d^	NT ^d^	4.66 (2.08–10.4)	100 ^e^
ICI-174,864 ^c^	―	422 (215–829)	NT ^d^	NT ^d^	114 (67.9–192)	−100 ^f^
NTI ^c^	―	0.457 (0.192–1.09)	30.7 (12.5–75.4)	14.7 (3.16–68.5)	ND ^g^	7.50 ^h^
**9a**	Me	284 (151–535)	10,100 (1920–16,600)	ND ^g^	0.468 (0.0816–2.68)	−48.8 (−58.2–−39.5)
**9b**	CF_3_	365 (193–692)	11,800 (1740–80,300)	675 (210–2170)	ND ^g^	−36.1 ^h^
**9c**	Ph	49.6 (26.2–93.9)	20,500 (1560–269,000)	95.6 (41.4–220)	ND ^g^	ND ^g^
**9d**	Bn	734 (326–1660)	5180 (1930–13,900)	18,800 (6010–58,800)	310 (82.1–177)	47.1 (36.9–57.3)
**9e**	Phenethyl	132 (57.0–304)	1400 (261–7440)	17,800 (6840–46,300)	75.8 (39.5–146)	88.1 (79.2–97.0)
**9f**	c-Pr	7.44 (3.53–15.7)	3900 (1450–10,500)	13.6 (5.45–33.7)	1.59 (0.825–3.05)	−80.5 (−87.7–−73.4)
**9g**	vinyl	196 (87.5–440)	3570 (1420–8970)	59,100 (2190–160,000)	7.80 (1.58–38.4)	−80.2 (−97.8–−62.6)

^a^ Evaluated by the ability of each compound to displace [^3^H]DAMGO (MOR), [^3^H]DPDPE (DOR), or [^3^H]U-69,593 (KOR) binding to the CHO cells expressing human MOR, DOR, or KOR. The data represent means of three samples. ^b^ Membranes were incubated with [^35^S]GTPγS and GDP in the presence of the test compound. The human DOR recombinant cell membrane (CHO) was used in this assay. The data represent means of three samples. ^c^ From ref. [24]. ^d^ NT: Not tested. ^e^ DPDPE (1 μM) was used as the standard DOR agonist. ^f^ ICI-174,864 (10 μM) was used as the standard DOR inverse agonist. ^g^ ND: Not determined. ^h^ Percent stimulation at 10 μM of the tested compound. 95% CI: 95% confidence interval.

**Table 2:**
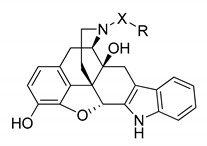
Comparison of binding affinity and functional activity for the DOR among the corresponding alkyl-, amide-, and sulfonamide-type NTI derivatives.

R	Alkyl-Type, X = CH_2_ (95% CI)	Amide-Type, X = CO (95% CI)	Sulfonamide-Type, X = SO_2_ (95% CI)
Me	SYK-323 ^a^ *K*_i_ = 2.71 nM (1.93–3.82) EC_50_: ND ^c^ E_max_: ND ^c^	SYK-747 ^b^ *K*_i_ = 977 nM (332–2,880) EC_50_: ND ^c^ E_max_ = −4.86% ^d^	**9a** (SYK-884) *K*_i_ = 284 nM (151–535) EC_50_ = 0.468 nM (0.0816–2.68) E_max_ = −48.8% (−58.2–−39.5)
CF_3_	SYK-165 ^a^ *K*_i_ = 134 nM (75.4–239) EC_50_ = 45.5 nM (17.5–118) E_max_ = −44.8% (−51.9–−37.7)	SYK-752 ^b^ *K*_i_ = 7.84 nM (4.30–14.3) EC_50_: ND ^c^ E_max_ = −1.10% ^d^	**9b** (SYK-837) *K*_i_ = 365 nM (193–692) EC_50_: ND ^c^ E_max_ = −36.1% ^d^
Ph	SYK-619 ^e^ *K*_i_ = 15.8 nM (7.23–34.7) EC_50_ = 16.1 nM (7.31–35.4) E_max_ = −56.5% (−63.3–−49.8)	SYK-736 ^b^ *K*_i_ = 290 nM (177–476) EC_50_ = 322 nM (51.5–2,010) E_max_ = −92.7% (−122–−62.6)	**9c** (SYK-838) *K*_i_ = 49.6 nM (26.2–93.9) EC_50_: ND ^c^ E_max_: ND ^c^
Bn	SYK-707 ^e^ *K*_i_ = 1.94 nM (1.50–2.50) EC_50_ = 15.1 nM (2.67–85.0) E_max_ = 40.3% (30.4–50.2)	SYK-754 ^f^ *K*_i_ = 89.0 nM (66.6–119) EC_50_ = 127 nM (80.1–201) E_max_ = 88.5% (81.6–95.3)	**9d** (SYK-887) *K*_i_ = 734 nM (326–1,660) EC_50_ = 310 nM (82.1–117) E_max_ = 47.1% (36.9–57.3)
Phenethyl	SYK-903 ^g^ *K*_i_ = 166 nM (65.5–419) EC_50_ = 7.37 nM (3.80–14.3) E_max_ = 105% (96.2–114)	SYK-753 ^f^ *K*_i_ = 41.5 nM (28.5–60.6) EC_50_ = 132 nM (51.5–336) E_max_ = 97.5% (82.6–112)	**9e** (SYK-901) *K*_i_ = 132 nM (57.0–304) EC_50_ = 75.8 nM (39.5–146) E_max_ = 88.1% (79.2–97.0)
*c*-Pr	NTI ^e^ *K*_i_ = 0.46 nM (0.192–1.09) EC_50_: ND ^c^ E_max_ = 7.50% ^d^	SYK-623 ^b^ *K*_i_ = 17.3 nM (10.3–28.9) EC_50_ = 0.969 nM (0.406–2.93) E_max_ = −91.2% (−99.8–−82.7)	**9f** (SYK-839) *K*_i_ = 7.44 nM (3.53–15.7) EC_50_ = 1.59 nM (0.825–3.05) E_max_ = −80.5% (−87.7–−73.4)
vinyl	SYK-706 ^e^ *K*_i_ = 0.609 nM (0.407–0.913) EC_50_ = 101 nM (4.46–2,280) E_max_ = 16.2% (8.14–24.3)	SYK-836 ^b^ *K*_i_ = 1.16 nM (0.508–2.63) EC_50_ = 1.43 nM (0.819–2.59) E_max_ = −86.2% (−92.2–−80.2)	**9g** (SYK-886) *K*_i_ = 196 nM (87.5–440) EC_50_ = 7.80 nM (1.58–38.4) E_max_ = −80.2% (−97.8–−62.6)

: Full agonist (E_max_ ≥ 80%),   : partial agonist (80% > E_max_ ≥ 10%),   : neutral antagonist (10% > E_max_ > −10%),   : partial inverse agonist (−10% ≥ E_max_ > -80%),   : full inverse agonist (−80% ≥ E_max_). ^a^ From ref. [26]. ^b^ From ref. [25]. ^c^ ND: Not determined. ^d^ Percent stimulation at 10 μM of the tested compound. ^e^ From ref. [24]. ^f^ From ref. [29]. ^g^ SYK-903 was newly synthesized and evaluated in comparison with SYK-836 and SYK-886. The synthetic method and its spectral data are described in the Supporting Information. The data represent means of three or four samples. 95% CI: 95% confidence interval.

## Supplementary Materials

The following are available online. Synthesis of **5** hydrochloride, previously reported synthetic method of **7**, optimization of the reaction conditions for synthesis of **5**, synthesis of SYK-903, and ^1^H and ^13^C NMR spectra are available online.
