# Supplementary material for: Effects of N-Substituents on the Functional Activities of Naltrindole Derivatives for the δ Opioid Receptor: Synthesis and Evaluation of Sulfonamide Derivatives
Source: Molecules. 2020 Aug 20;25(17):3792. doi: 10.3390/molecules25173792 (PMC7503831; doi:10.3390/molecules25173792)
Supplement: Supplementary file 1 [file molecules-25-03792-s001.pdf]

Supporting Information

Effects of *N*-Substituents on the Functional Activities of Naltrindole Derivatives for the δ Opioid Receptor: Synthesis and Evaluation of Sulfonamide Derivatives

Chiharu Iwamatsu[1], Daichi Hayakawa[2], Tomomi Kono[1], Ayaka Honjo[1], Saki Ishizaki[1], Shigeto Hirayama[1,3], Hiroaki Gouda[2] and Hideaki Fujii[1,2,*]

[1] Laboratory of Medicinal Chemistry, School of Pharmacy, Kitasato University, 5-9-1, Shirokane, Minato-ku, Tokyo 108-8641, Japan.

[2] School of Pharmacy, Showa University, 1-5-8 Hatanodai, Shinagawa-ku, Tokyo 142-8555, Japan.

[3] Medicinal Research Laboratories, School of Pharmacy, Kitasato University, 5-9-1, Shirokane, Minato-ku, Tokyo 108-8641, Japan.

* Correspondence: fujiih@pharm.kitasato-u.ac.jp

Table of Contents

1. Synthesis of nor-NTI (**5**) hydrochloride S2
2. Previously reported synthetic method of the key intermediate **7** S2
3. Optimization of the reaction conditions for synthesis of nor-NTI (**5**) S4
4. Synthesis of NTI derivative (SYK-903) with *N*-(3-phenylpropyl) group S5
5. ^{1}H and ^{13}C NMR spectra S8
6. References S28

1. Synthesis of nor-NTI (5) hydrochloride

Portoghese *et al.* and Rice *et al.* independently reported the synthesis of nor-NTI (**5**) from noroxymorphone by the Fischer indole synthesis (Scheme S1) [1, 2]. This method is efficient from the viewpoints of both the yield and the number of reaction steps. However, noroxymorphone is difficult to obtain and very expensive compared with naltrexone hydrochloride (**1**).

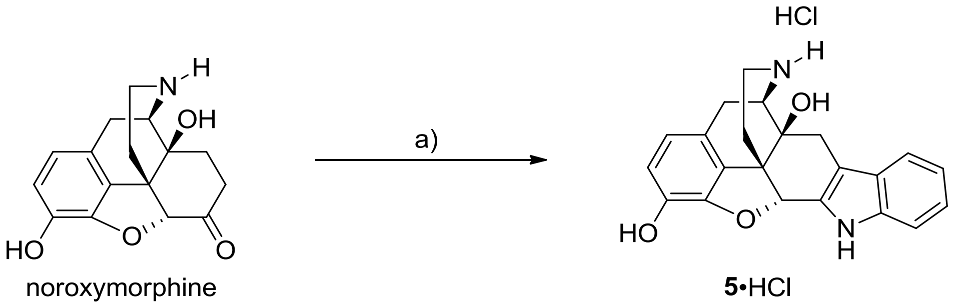

Scheme S1. Synthesis of nor-NTI (**5**) hydrochloride. Reagents and conditions: a) $PhNHNH_2$·HCl, HCl/MeOH, MeOH, reflux, 98-99%.

2. Previously reported synthetic method of the key intermediate 7

We previously reported the synthesis of the key intermediate **7** as shown in Scheme S2 [3]. The 3-*O*-methylation of naltrexone hydrochloride (**1**) and the subsequent acetalization provided compound **S2** [4-6]. After acetylation of the 14-hydroxy group in **S2**, the treatment of the obtained **S3** with Troc-Cl afforded compound **S4**. Both the carbamate and acetate were hydrolyzed under the basic conditions at the same time and the following deacetalyzation gave noroxycodone. The Fischer indolization of noroxycodone with phenylhydrazine hydrochloride yielded nor-NTI-3-*O*-methyl ether (**S6**). The exchange of the protective groups from methyl into TBS group provided the key intermediate **7** [3].

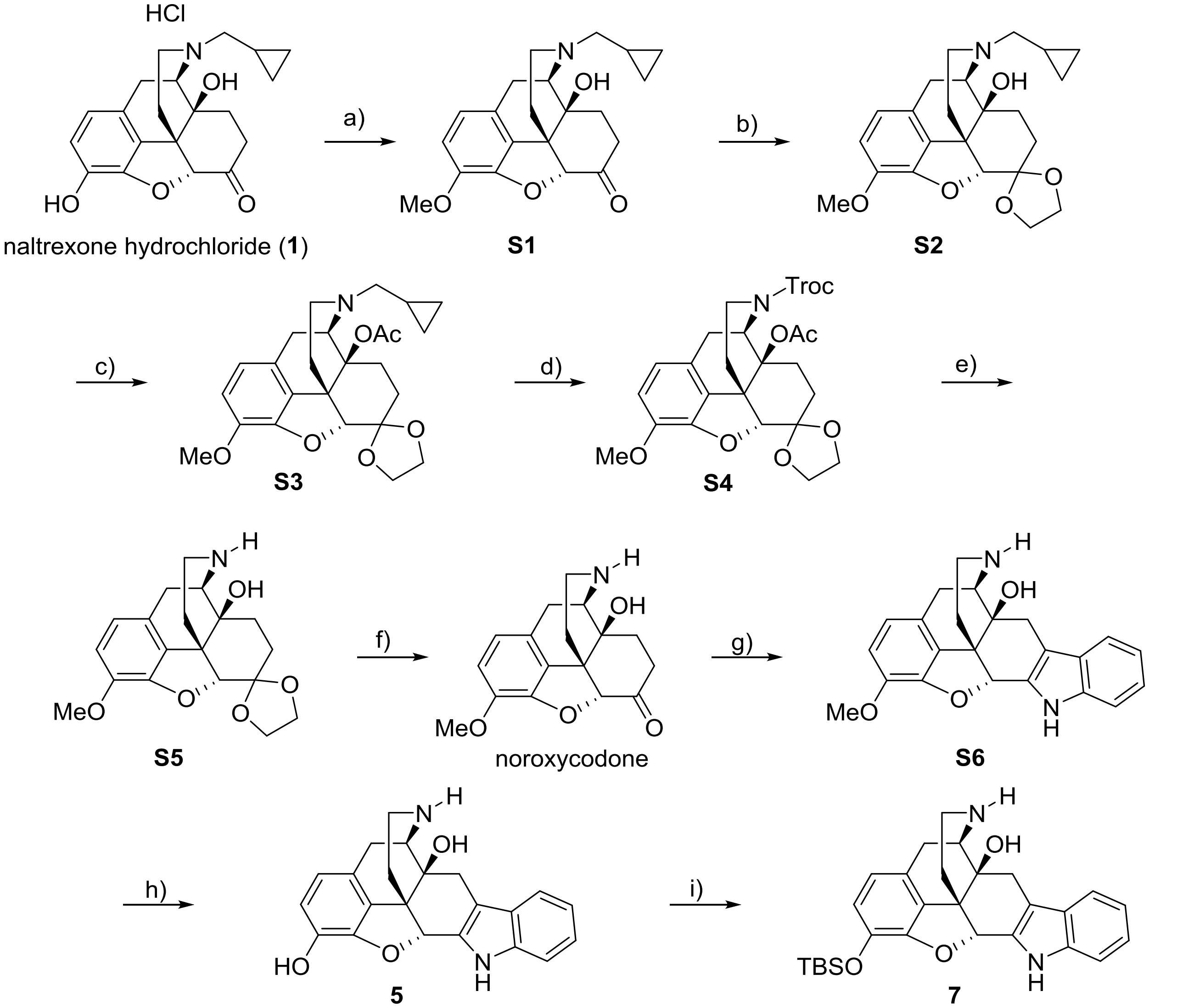

Scheme S2. Synthesis of the key intermediate **7**. Reagents and conditions: a) MeI, K_2CO_3, DMF, rt, 92%; b) ethylene glycol, *p*-TsOH·H_2O, toluene, reflux; c) Ac_2O, 85 °C, 92% (from **S1**); d) Troc-Cl, K_2CO_3, 1,1,2,2-tetrachloroethane, reflux, 92%; e) 12 M KOH aq, DMSO, 120 °C, 71%; f) 2 M HCl, MeOH, reflux; g) $PhNHNH_2$·HCl, $MeSO_3H$, EtOH, reflux, 84% (from **S5**); h) 1 M BBr_3, CH_2Cl_2, -10 °C, i) TBSCl, imidazole, DMF, rt, 87% (from **S6**).

3. Optimization of the reaction conditions for synthesis of nor-NTI (5)

The treatment of a mixture of **4** and **4′** with 4 M NaOH aqueous solution using methanol as a solvent gave compounds **5** and **6** in 60% and 21% yields, respectively (Scheme S3).

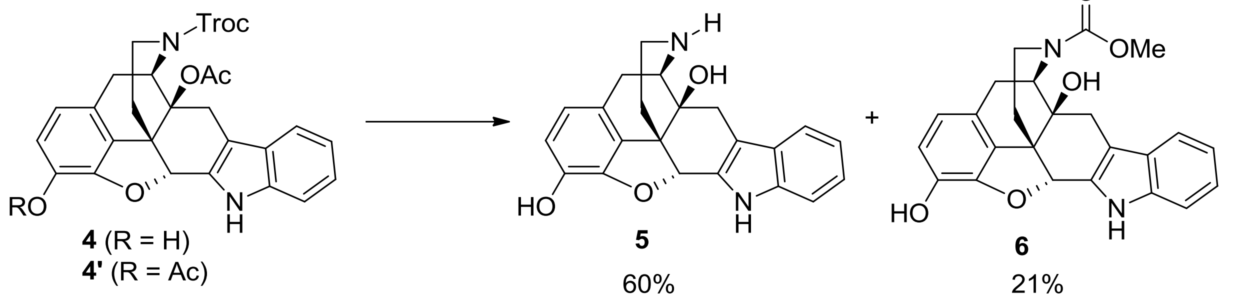

Scheme S3. Hydrolysis of the mixture of compounds **4** and **4′** in methanol. Reagents and conditions: a) 4 M NaOH aq, MeOH, reflux.

We speculated that nor-NTI (**5**) was obtained by the mechanism as follows: at first, the hydrolysis of the acetate moiety provides the alkoxide anion, which intramolecularly attacks the carbonyl carbon of the carbamate to afford oxazolidinone **A**. In general, the hydrolysis of carbamates requires harsh reaction conditions. However, the

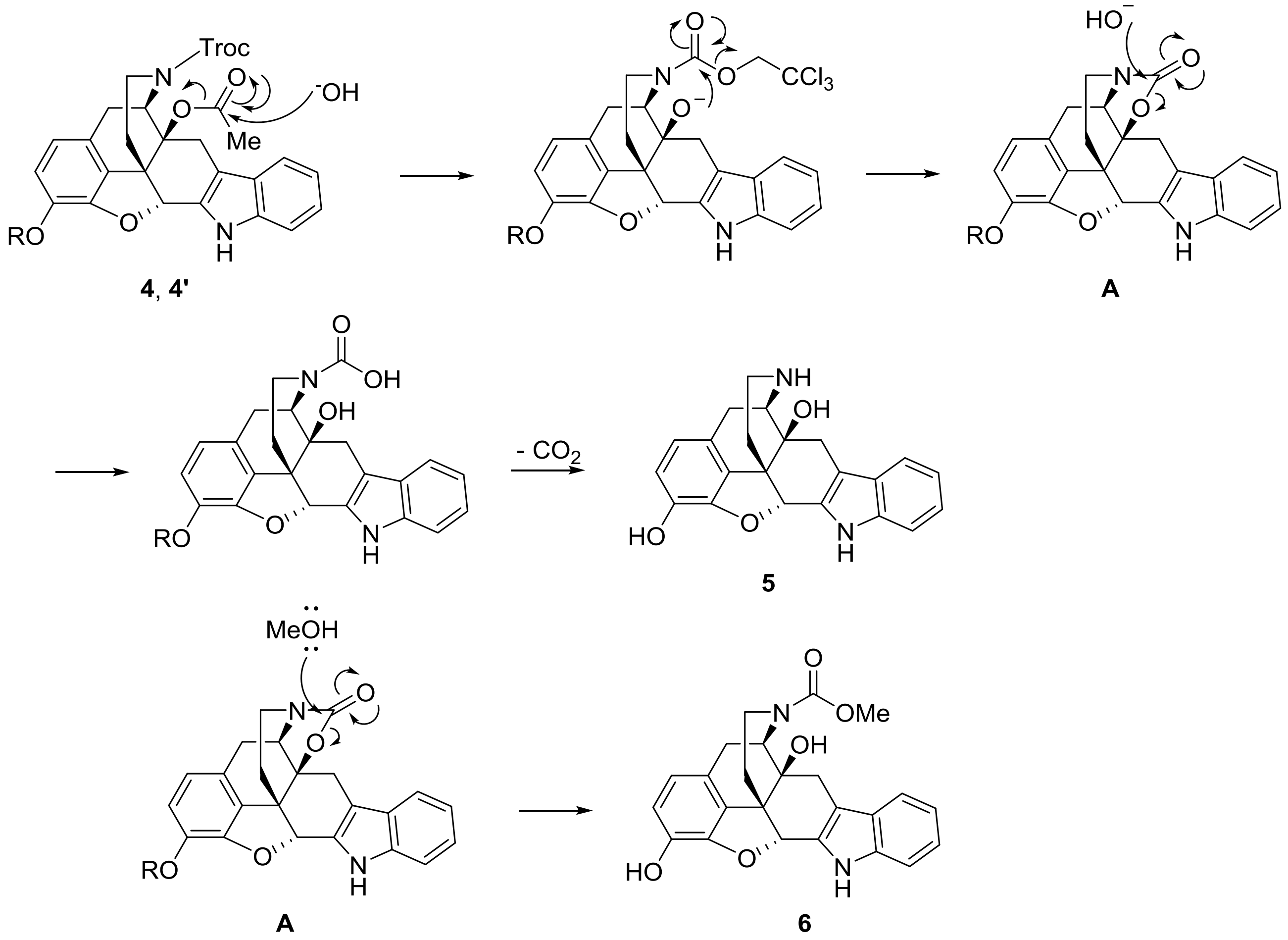

Scheme S4. Proposed reaction mechanism for providing compounds **5** and **6**.

oxazolidinone moiety in **A** would be prone to undergo hydrolysis due to its strained structure. As a result, the hydrolysis of oxazolidinone **A** proceeds under milder reaction conditions to provide nor-NTI (**5**). In the case that methanol was used as a solvent, the solvolysis of oxazolidinone **A** would concomitantly occur to give methyl carbamate **6** (Scheme S4). Based on the proposed reaction mechanism, the usage of a non-nucleophilic solvent would hamper the preparation of carbamates like **6**. Indeed, the usage of THF as a solvent instead of methanol successfully provided only the target compound **5**.

4. Synthesis of NTI derivative (SYK-903) with *N*-(3-phenylpropyl) group

SYK-903 was prepared from compound **7** (Scheme S5).

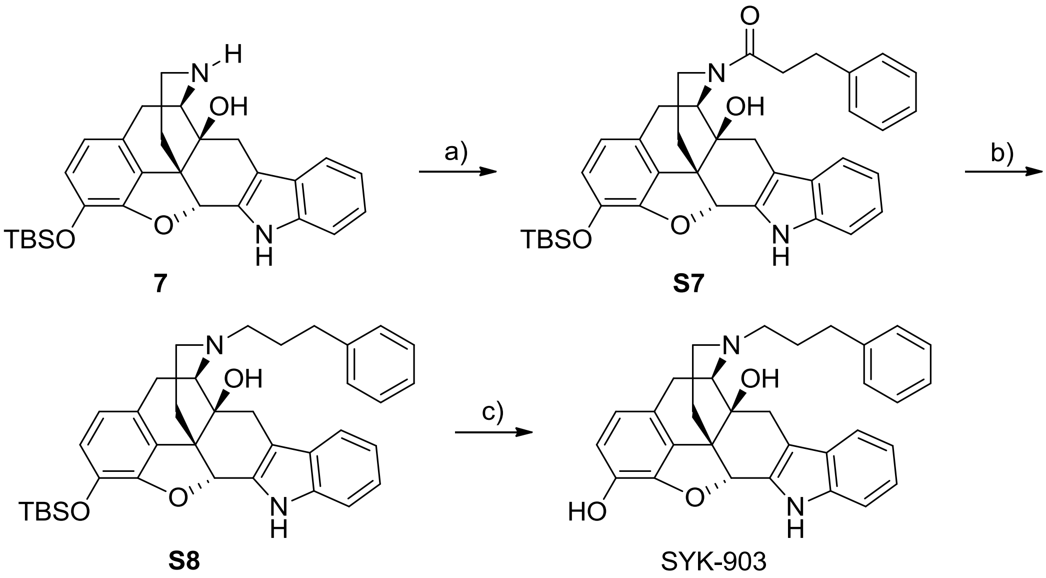

Scheme S5. Synthesis of SYK-903. Reagents and conditions: a) 3-phenylpropanoyl chloride, Et_3N, CH_2Cl_2, rt, 67%; b) BH_3·THF, THF, reflux, 90%; c) TBAF, THF, rt, 91%.

4.1. 1-(3-((*tert*-Butyldimethylsilyl)oxy)-6,7-didehydro-4,5α-epoxy-14β-hydroxyindolo[2′,3′:6,7]morphinan-17-yl)-3-phenylpropan-1-one (S7)

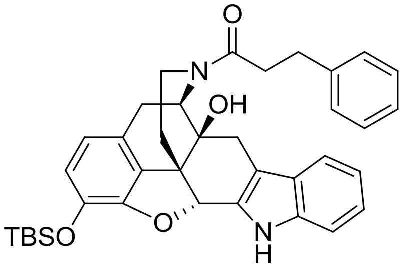

S7

Under an Ar atmosphere, to a solution of compound **7** (132 mg, 0.28 mmol) in dichloromethane (1.3 mL) were added triethylamine (0.12 mL, 0.84 mmol) and 3-phenylpropanoyl chloride (80 μL, 0.56 mmol), and the mixture was stirred at room temperature for 1 hour. The reaction mixture was poured into saturated sodium bicarbonate aqueous solution and extracted with chloroform. Combined organic layers were washed with brine and dried over anhydrous

sodium sulfate. After removing the solvent *in vacuo*, the residue was purified by silica gel column chromatography to give compound **S7** (113 mg, 67%) as a light tan amorphous material; IR (film) cm^{-1}: 3415, 3026, 2928, 2857, 1713, 1614, 1496, 1444, 1328, 1273, 1214, 1162, 1112. ^1H NMR (400 MHz, $CDCl_3$): δ 0.00 (s, 2.1H), 0.02 (s, 0.9H), 0.03 (s, 2.1H), 0.04 (s, 0.9H), 0.88 (s, 9H), 1.67 (dd, *J* = 2.4, 12.5 Hz, 0.7H), 1.79 (dd, *J* = 2.8, 12.9 Hz, 0.3H), 2.28-2.43 (m, 1H), 2.62-3.08 (m, 7.3H), 3.13 (ddd, *J* = 3.6, 13.4, 13.4 Hz, 0.7H), 3.25-3.38 (m, 1H), 3.67 (dd, *J* = 4.6, 13.8 Hz, 0.7H), 4.28 (d, *J* = 6.5 Hz, 0.3H), 4.63 (dd, *J* = 4.7, 13.7 Hz, 0.3H), 5.28 (d, *J* = 6.7 Hz, 0.7H), 5.55 (s, 0.3H), 5.57 (s, 0.7H), 6.52 (d, *J* = 8.0 Hz, 0.3H), 6.53 (d, *J* = 8.1 Hz, 0.7H), 6.59 (br d, *J* = 8.1 Hz, 1H), 7.05 (br t, *J* = 7.4 Hz, 1H), 7.15-7.36 (m, 7H), 7.38 (d, *J* = 8.0 Hz, 0.3H), 7.41 (d, *J* = 7.9 Hz, 0.7H), 8.09 (br s, 0.7H), 8.17 (br s, 0.3H), a proton (OH) was not observed. ^{13}C NMR (100MHz, $CDCl_3$): δ -4.8, -4.7, 18.2, 25.5, 29.4, 29.7, 29.8, 29.9, 30.7, 31.49, 31.53, 32.5, 32.9, 34.6, 35.4, 39.1, 48.0, 48.2, 53.6, 58.3, 73.4, 73.6, 83.8, 84.0, 110.0, 110.6, 111.1, 111.3, 118.7, 118.9, 119.0, 119.4, 119.5, 122.3, 122.4, 122.9, 123.1, 124.8, 125.4, 126.1, 126.18, 126.20, 126.7, 128.27, 128.37, 128.45, 128.50, 128.7, 130.16, 130.21, 137.06, 137.11, 138.7, 138.8, 141.0, 141.4, 146.4, 146.5, 172.1, 172.4. HR-MS (ESI): Calcd for $C_{37}H_{43}N_2O_4Si$ $[M+H]^+$: 607.2992. Found: 607.3019.

4.2. 3-((*tert*-Butyldimethylsilyl)oxy)-6,7-didehydro-4,5α-epoxy-17-(3-phenylpropyl)indolo[2′,3′:6,7]morphinan-14β-ol (S8)

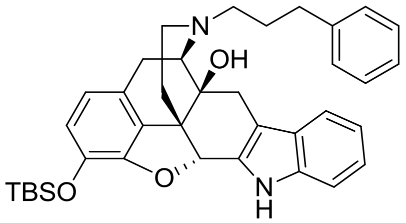

S8

Under an Ar atmosphere, to a solution of compound **S7** (113 mg, 0.19 mmol) in THF (6 mL) was added 1.0 M solution of borane-THF complex in THF (1.1 mL, 1.1 mmol), and the mixture was refluxed with stirring for 24 hours. After cooling to room temperature, to the reaction mixture was added saturated sodium bicarbonate aqueous solution and extracted with chloroform. The combined organic layers were washed with brine and dried over anhydrous sodium sulfate. After removing the solvent *in vacuo*, the residue was purified by silica gel column chromatography to give compound **S8** (84 mg, 90%) as a white amorphous material; IR (film) cm^{-1}: 2928, 1496, 1444, 1328, 1270, 1161, 960, 854, 740, 425, 408. ^1H NMR (400 MHz, $CDCl_3$): δ -0.01 (s, 3H), 0.03 (s, 3H), 0.88 (s, 9H), 1.73-1.94 (m, 3H), 2.26-2.40 (m, 2H), 2.48-2.64 (m, 3H), 2.62 (dd, *J* = 1.1, 15.7 Hz, 1H), 2.70 (d, *J* = 7.6 Hz, 2H), 2.81 (dd, *J* = 6.5, 18.8 Hz, 1H), 2.87 (d, *J* = 15.7 Hz, 1H), 3.12 (d, *J* = 6.5 Hz, 1H), 3.15 (d, *J* = 18.8 Hz, 1H), 4.84 (br s, 1H), 5.61 (s, 1H), 6.50 (d, *J* = 8.2 Hz, 1H), 6.54 (d, *J* = 8.2 Hz, 1H), 7.02 (ddd, *J* = 0.9, 7.1, 7.9 Hz, 1H), 7.14 (ddd, *J* = 1.1, 7.1, 8.1 Hz, 1H), 7.18-7.24 (m, 3H), 7.26-7.33 (m, 3H), 7.40 (d, *J* = 7.9 Hz, 1H), 8.01 (br s, 1H). ^{13}C NMR (100MHz, $CDCl_3$): δ -4.8, -4.7, 18.2, 23.6, 25.6, 28.8, 29.2, 31.4, 33.5, 43.5, 47.9, 53.9, 63.2, 72.6, 84.5, 111.0, 111.5, 118.5, 118.9, 119.2, 121.8, 122.7, 125.9, 126.3, 126.8, 128.36, 128.42, 129.0, 131.2, 137.1, 138.3, 141.8, 146.4. HR-MS (ESI): Calcd for $C_{37}H_{45}N_2O_3Si$ $[M+H]^+$: 593.3199. Found: 593.3855.

4.3. 6,7-Didehydro-4,5α-epoxy-17-(3-phenylpropyl)indolo[2′,3′:6,7]morphinan-3,14β-diol (SYK-903)

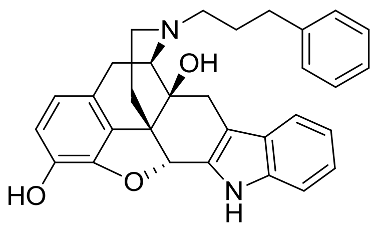

SYK-903

Under an Ar atmosphere, to a solution of compound **S8** (75 mg, 0.13 mmol) in THF (0.7 mL) was added 1.0 M solution of tetrabutylammonium fluoride in THF (0.15 mL, 0.15 mmol), and the mixture was stirred at room temperature for 1.5 hours. The reaction mixture was poured into saturated sodium bicarbonate aqueous solution and extracted with chloroform. The combined organic layers were washed with brine and dried over anhydrous sodium sulfate. After removing the solvent *in vacuo*, the residue was purified by silica gel column chromatography to give SYK-903 (55 mg, 91%) as a light tan oil; IR (neat) cm^{-1}: 3398, 2928, 1455, 1326, 1159, 1116, 960, 742, 699. ^{1}H NMR (400 MHz, $CDCl_3$): δ 1.76 (br d, *J* = 12.6 Hz, 1H), 1.82-1.94 (m, 2H), 2.23-2.41 (m, 2H), 2.50-2.61 (m, 3H), 2.62 (dd, *J* = 0.9, 15.8 Hz, 1H), 2.70 (t, *J* = 7.6 Hz, 2H), 2.79 (dd, *J* = 6.5, 18.6 Hz, 1H), 2.88 (d, *J* = 15.8 Hz, 1H), 3.12 (d, *J* = 6.5 Hz, 1H), 3.13 (d, *J* = 18.6 Hz, 1H), 5.71 (s, 1H), 6.47 (d, *J* = 8.1 Hz, 1H), 6.55 (d, *J* = 8.1 Hz, 1H), 7.01 (ddd, *J* = 0.9, 7.0, 7.9 Hz, 1H), 7.12 (ddd, *J* = 1.1, 7.9, 8.1 Hz, 1H), 7.18-7.24 (m, 2H), 7.24-7.28 (m, 2H), 7.28-7.34 (m, 2H), 7.41 (d, *J* = 7.9 Hz, 1H), 8.20 (br s, 1H), two protons (OH) were not observed. ^{13}C NMR (100MHz, $CDCl_3$): δ 18.3, 23.4, 29.2, 33.4, 43.4, 48.0, 53.8, 58.4, 63.2, 72.9, 85.5, 111.3, 111.4, 117.2, 118.9, 119.1, 119.2, 122.7, 125.1, 125.9, 126.5, 128.3, 128.4, 128.7, 130.5, 137.2, 138.9, 141.7, 142.6. HR-MS (ESI): Calcd for $C_{31}H_{31}N_2O_3$ $[M+H]^+$: 479.2341. Found: 479.2334. *Anal.* Calcd for $C_{31}H_{30}N_2O_3 \cdot 1.3H_2O \cdot 0.1CHCl_3$: C, 72.68; H, 6.41; N, 5.45. Found: C, 72.57; H, 6.16; N, 5.28.

5. ^{1}H and ^{13}C NMR spectra

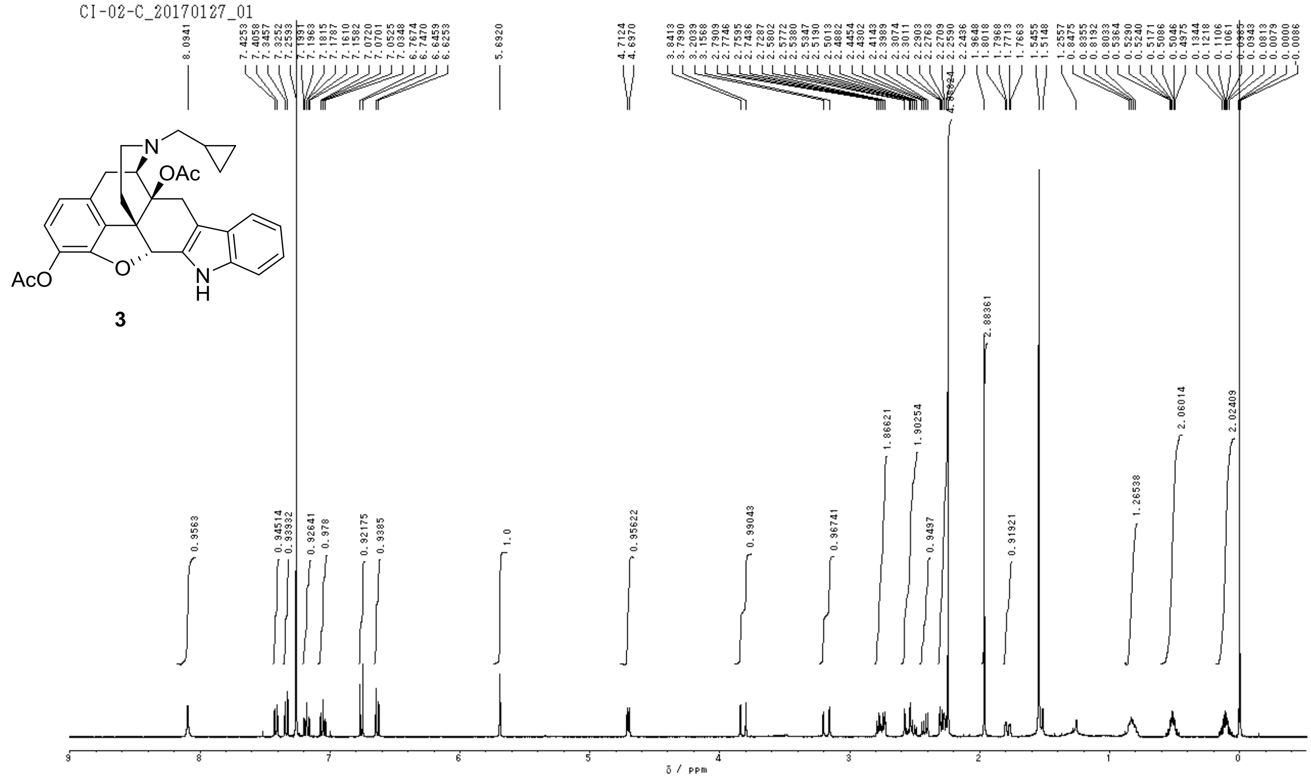

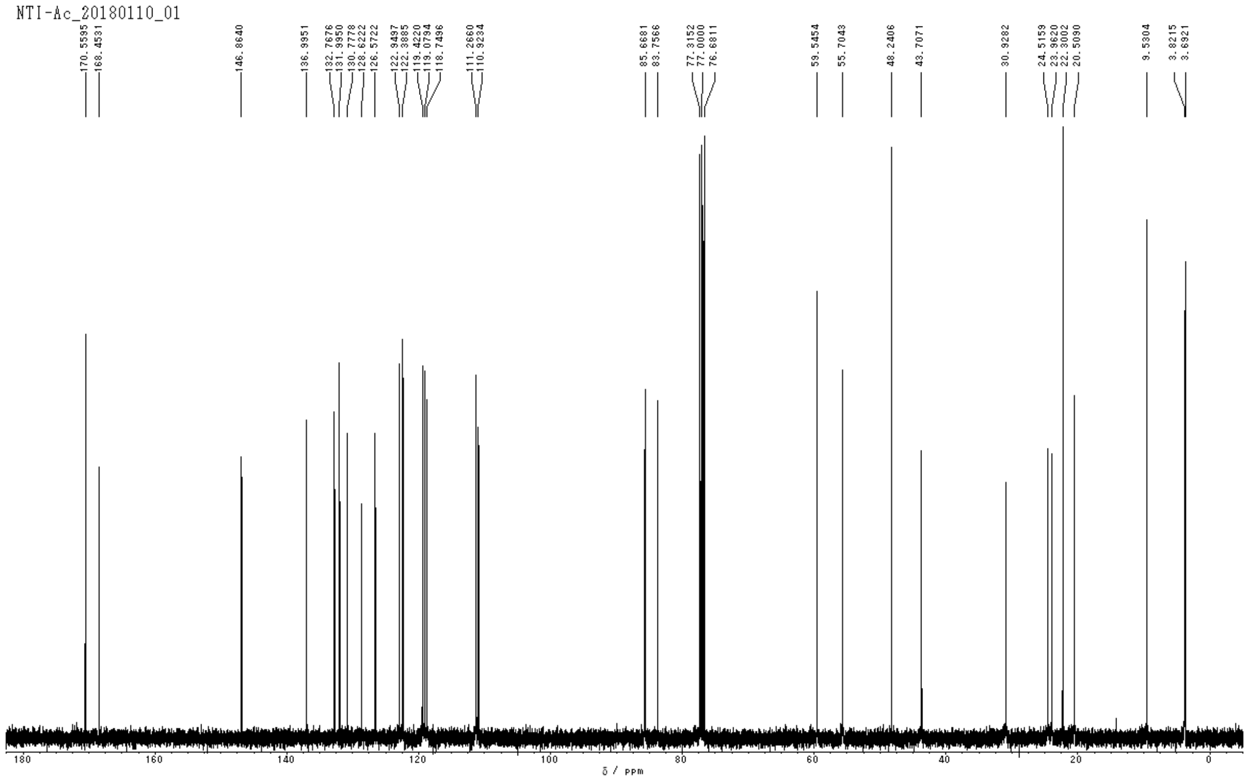

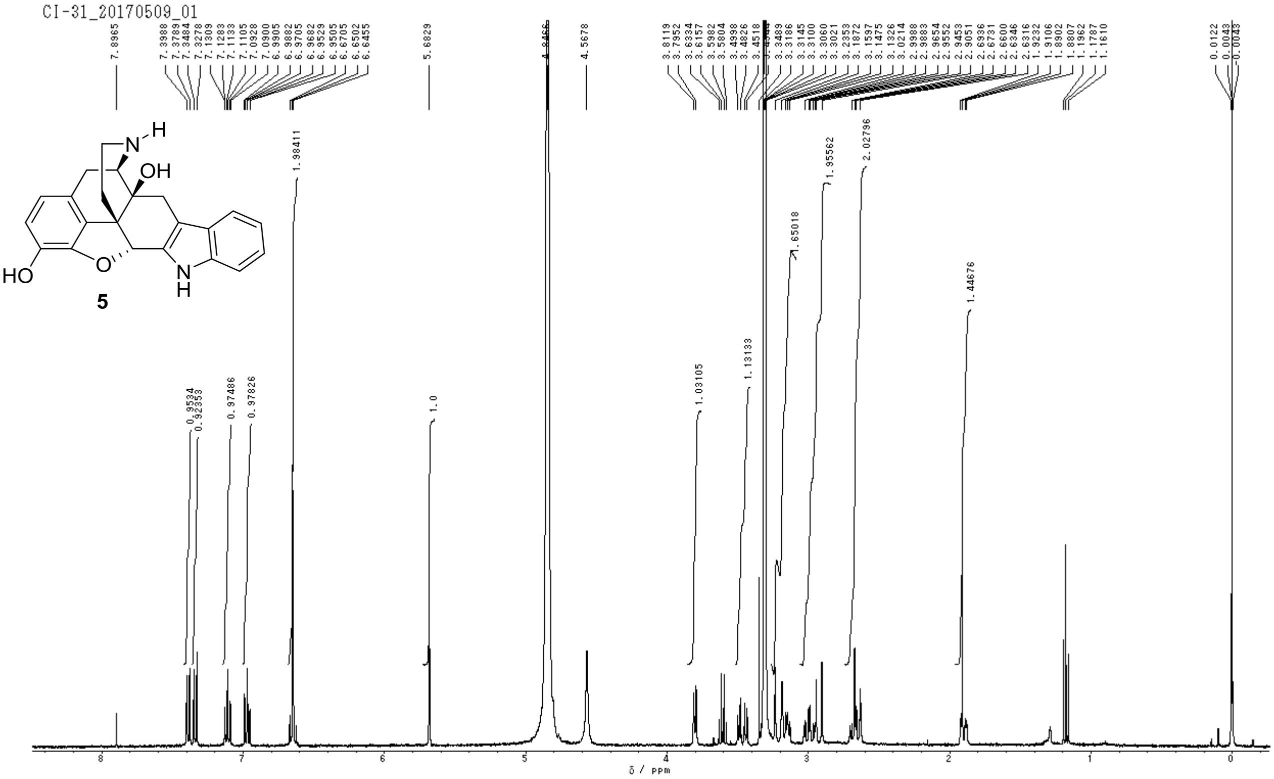

CI-31_20170509_01
N–H
OH
HO
O
NH
5
δ / ppm

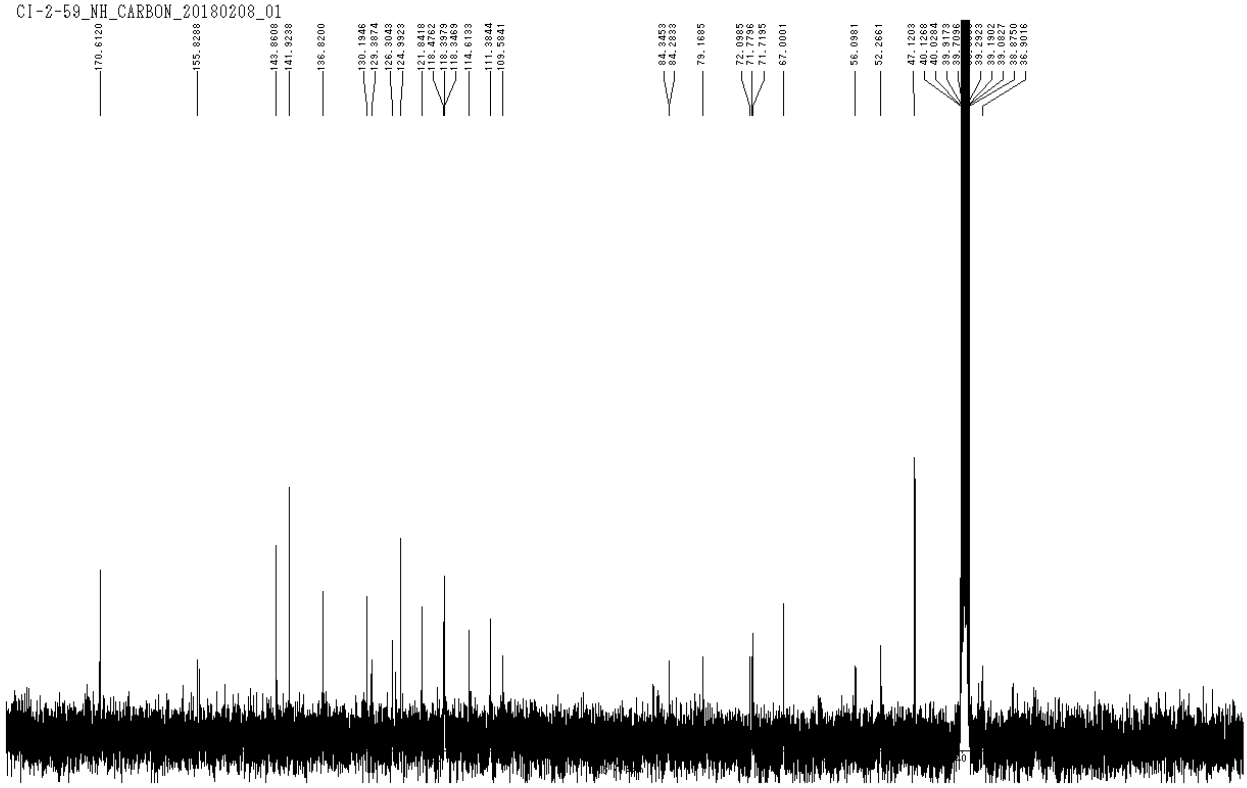

CI-2-59_NH_CARBON_20180208_01
170.6120
155.8288
143.8608
141.9238
136.8200
130.1946
129.3874
126.3043
124.9923
121.8418
118.4762
118.3979
118.3469
114.6133
111.3844
109.5841
84.3453
84.2833
79.1685
72.0985
71.7796
71.7195
67.0001
56.0981
52.2661
47.1203
40.1268
40.0284
39.9173
39.7036
39.2923
39.1902
39.0827
38.8750
36.9016

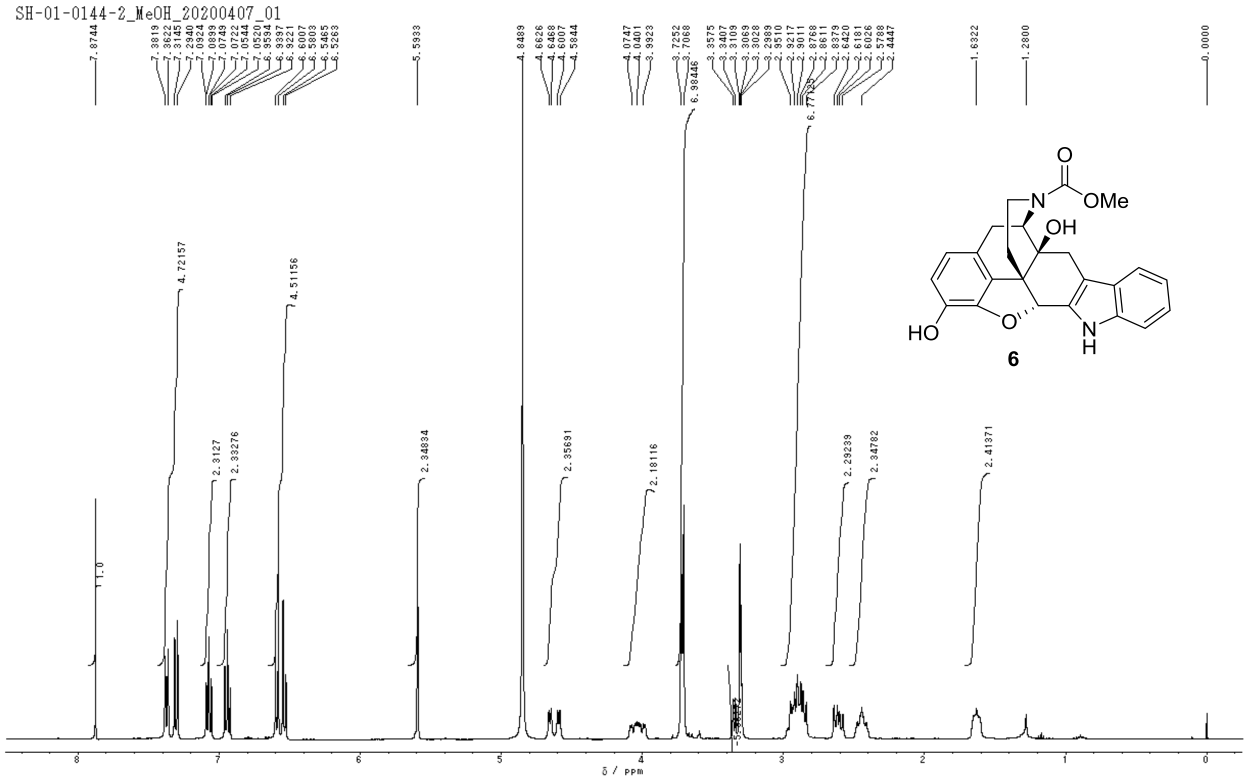
SH-01-0144-2_MeOH_20200407_01
7.8744
7.3819 7.3622 7.3145 7.2940 7.0924 7.0899 7.0749 7.0722 7.0544 7.0520 6.9394 6.9337 6.9221 6.6007 6.5803 6.5465 6.5283
5.5933
4.8489
4.6626 4.6468 4.6007 4.5844
4.0747 4.0401 3.9923
3.7252 3.7068
3.3575 3.3407 3.3109 3.3069 3.3028 3.2989 2.9510 2.9217 2.9011 2.8768 2.8611 2.8379 2.6420 2.6181 2.6026 2.5788 2.4447
1.6322
1.2800
0.0000
1.0
4.72157
2.3127
2.33276
4.51156
2.34834
2.35691
2.18116
6.98446
6.77135
2.29239
2.34782
2.41371
δ / ppm
O
N
OMe
OH
HO
O
N
H
6

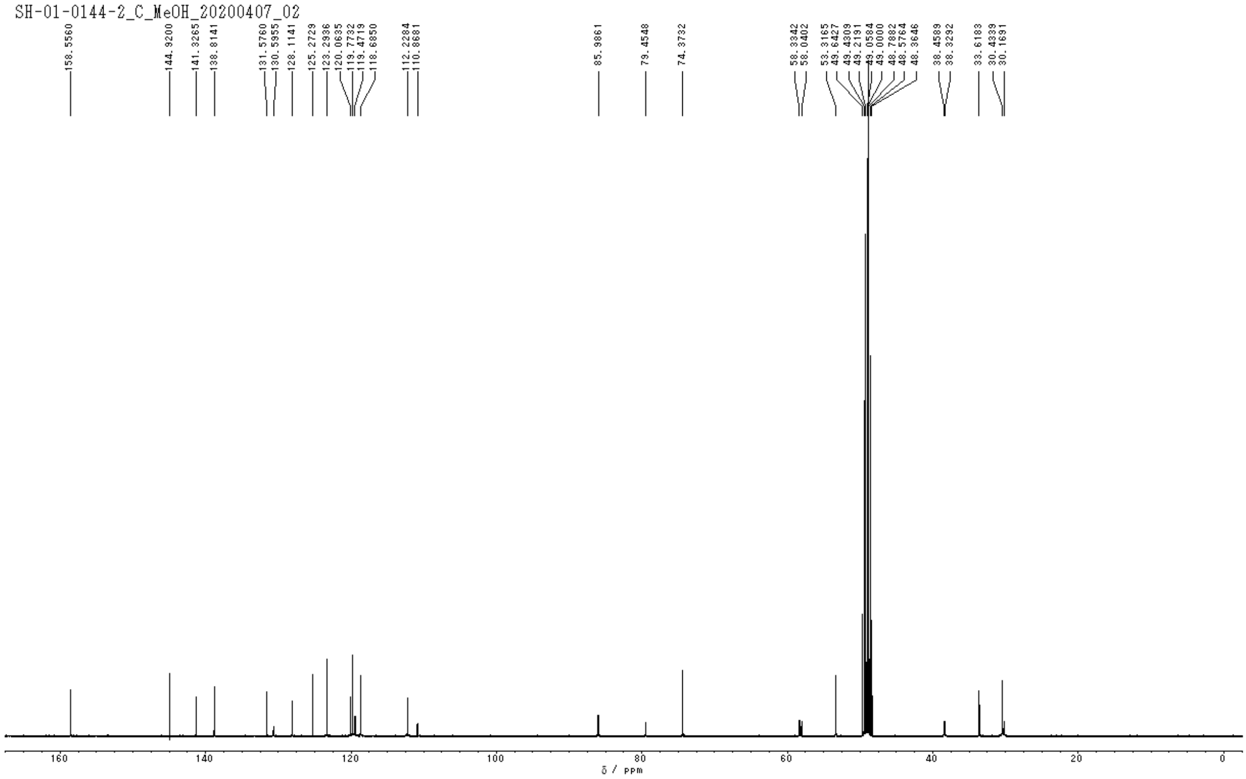
SH-01-0144-2_C_MeOH_20200407_02
158.5560
144.9200 141.3265 138.8141
131.5760 130.5955 128.1141 125.2729 123.2936 120.0635 119.7732 119.4719 118.6850
112.2284 110.8681
85.9861
79.4548
74.3732
58.3342 58.0402
53.3165 49.6427 49.4309 49.2191 49.0584 49.0000 48.7882 48.5764 48.3646
38.4589 38.3292
33.6183 30.4339 30.1691
δ / ppm

CI-2-62-proton_20171205_01

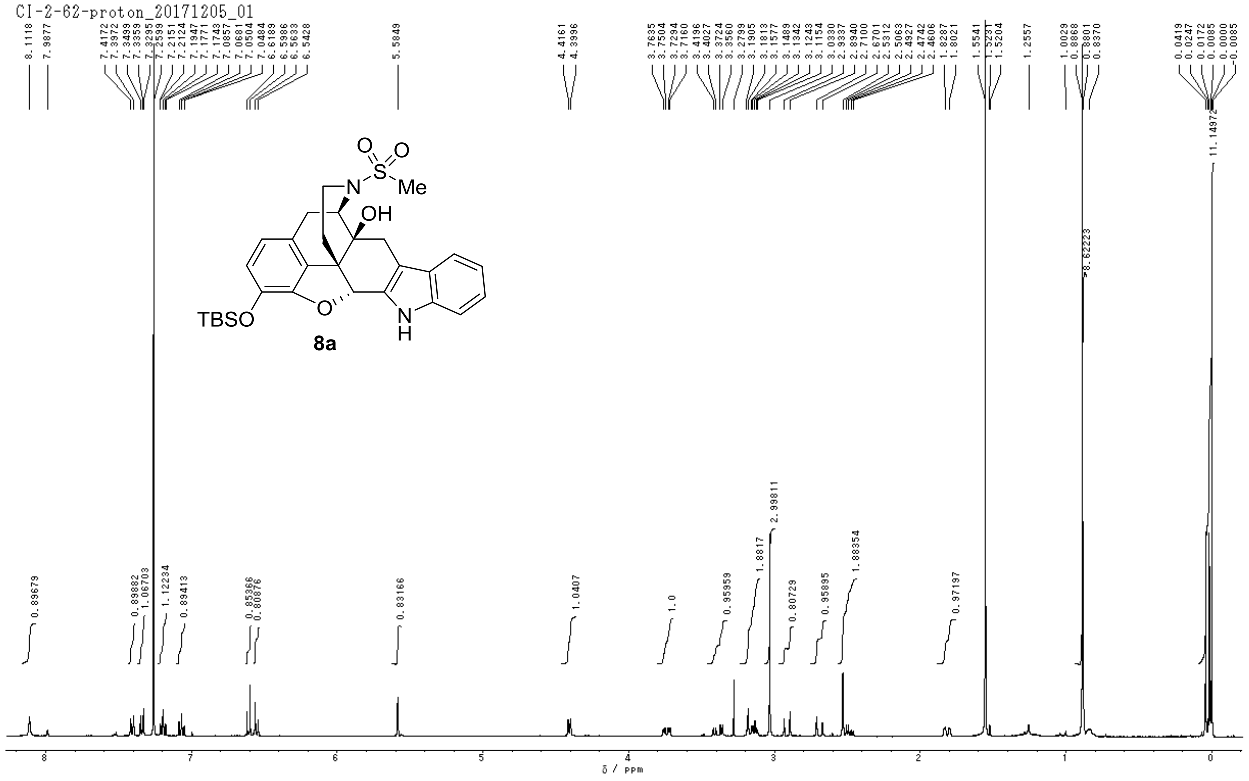

CI-2-63-2-CARBON_20171213_01

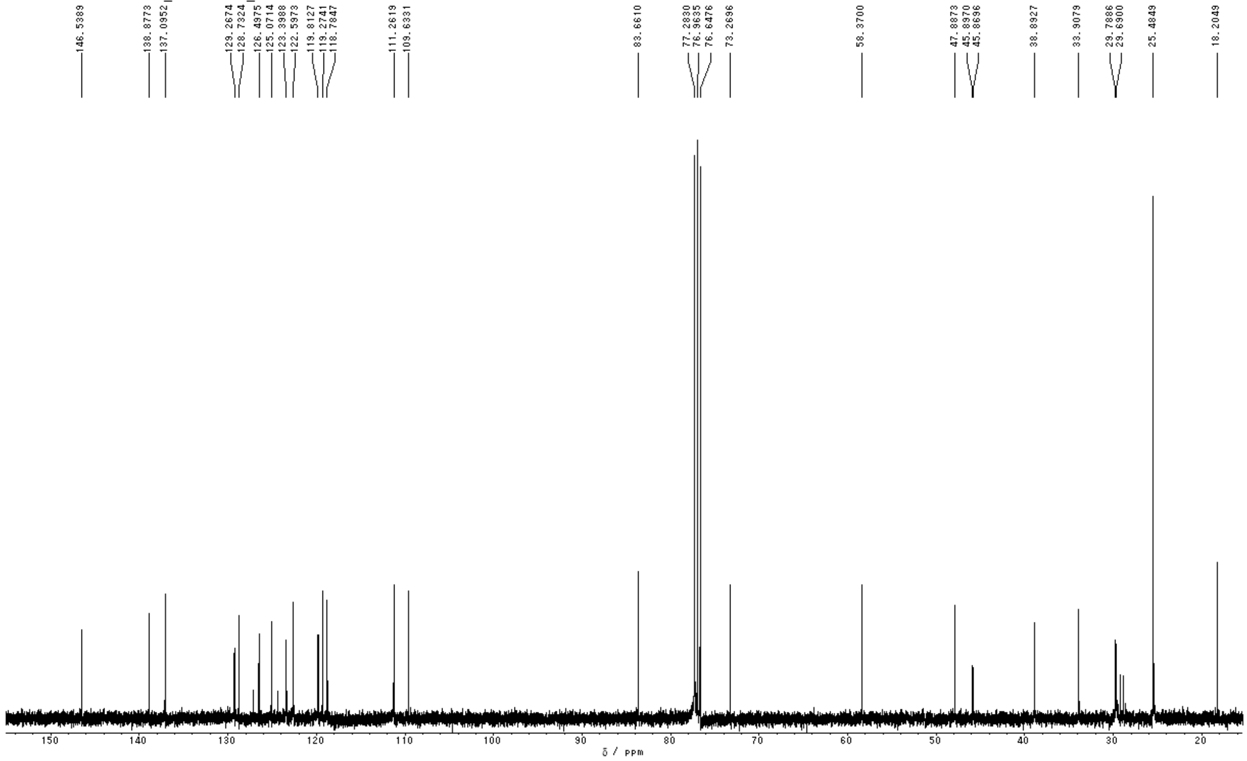

CI-2-47_20171101_01

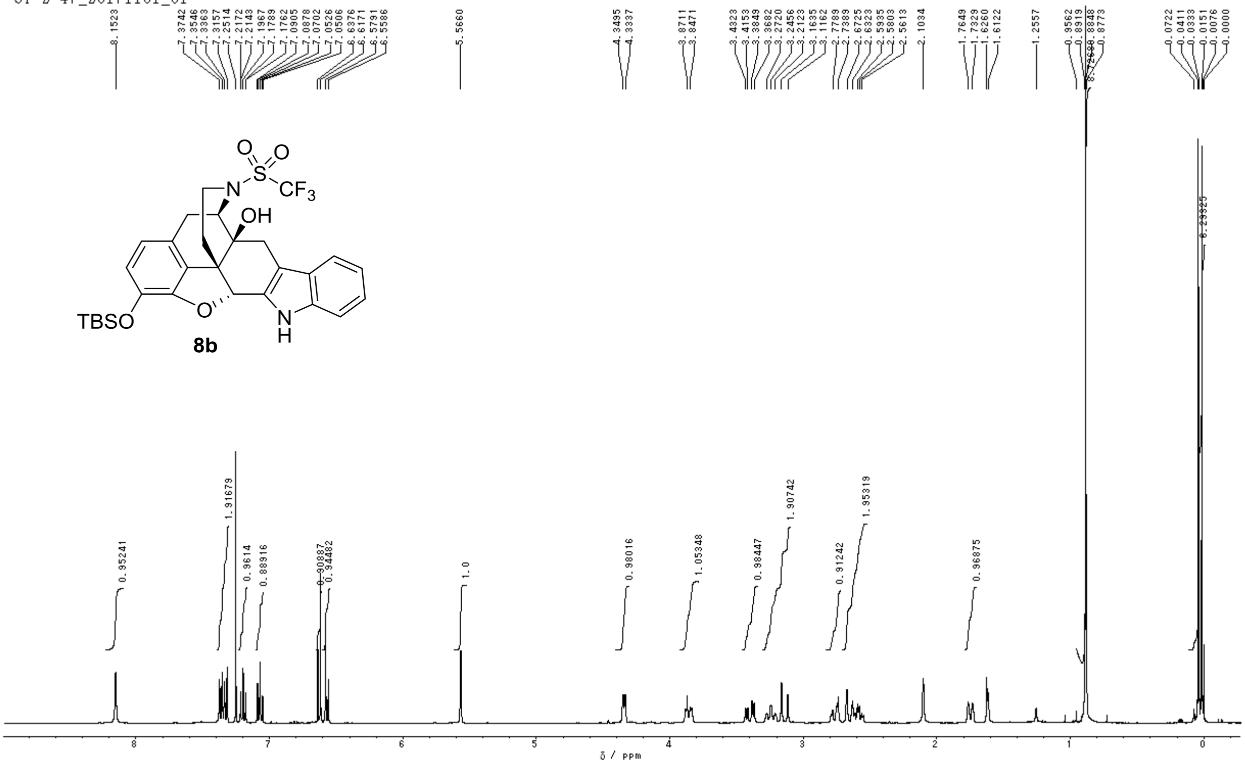

CI-2-47_20171101_01

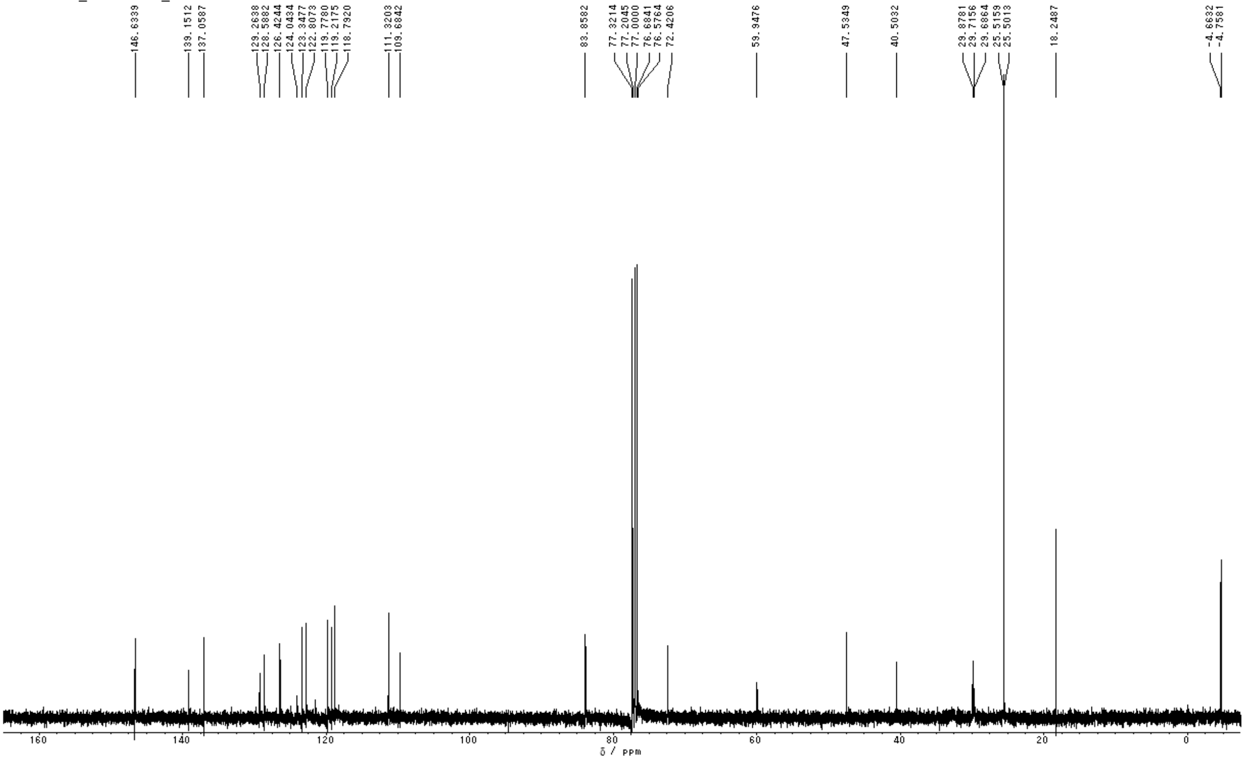

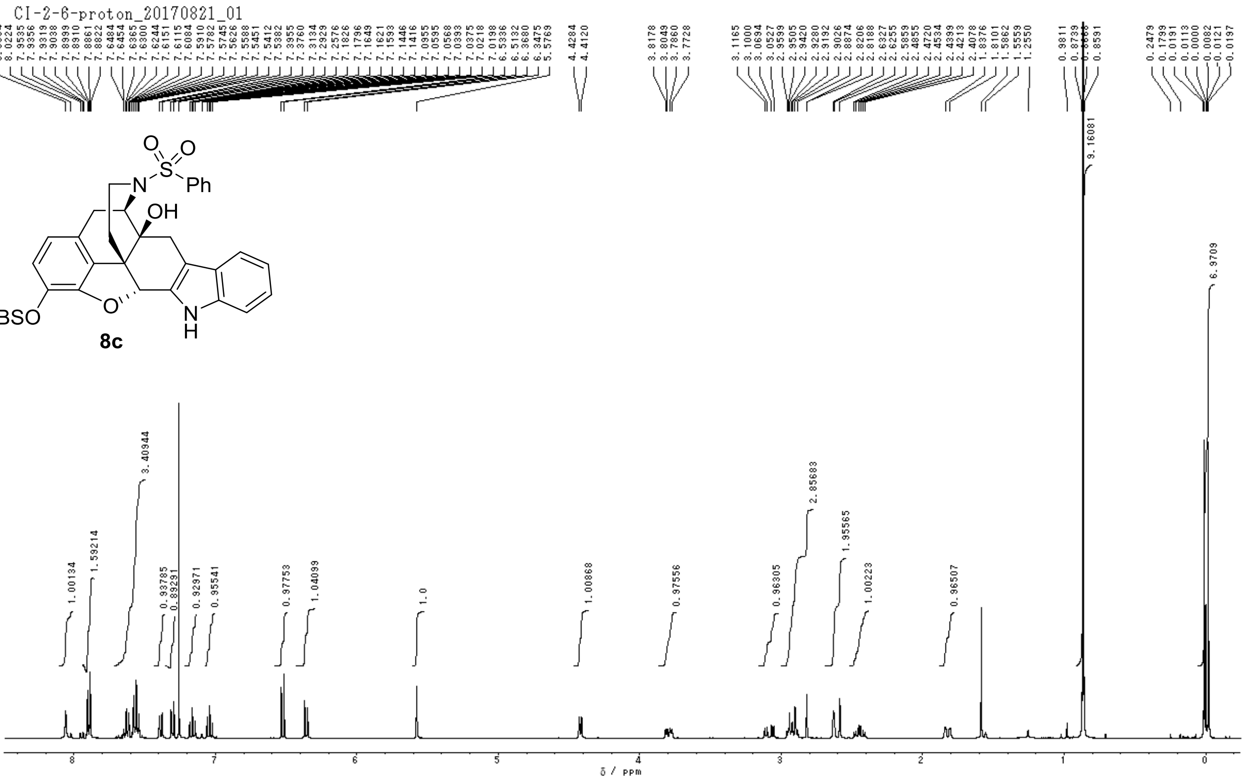
CI-2-6-proton_20170821_01
O
O
S
N
Ph
OH
TBSO
O
N
H
8c
δ / ppm

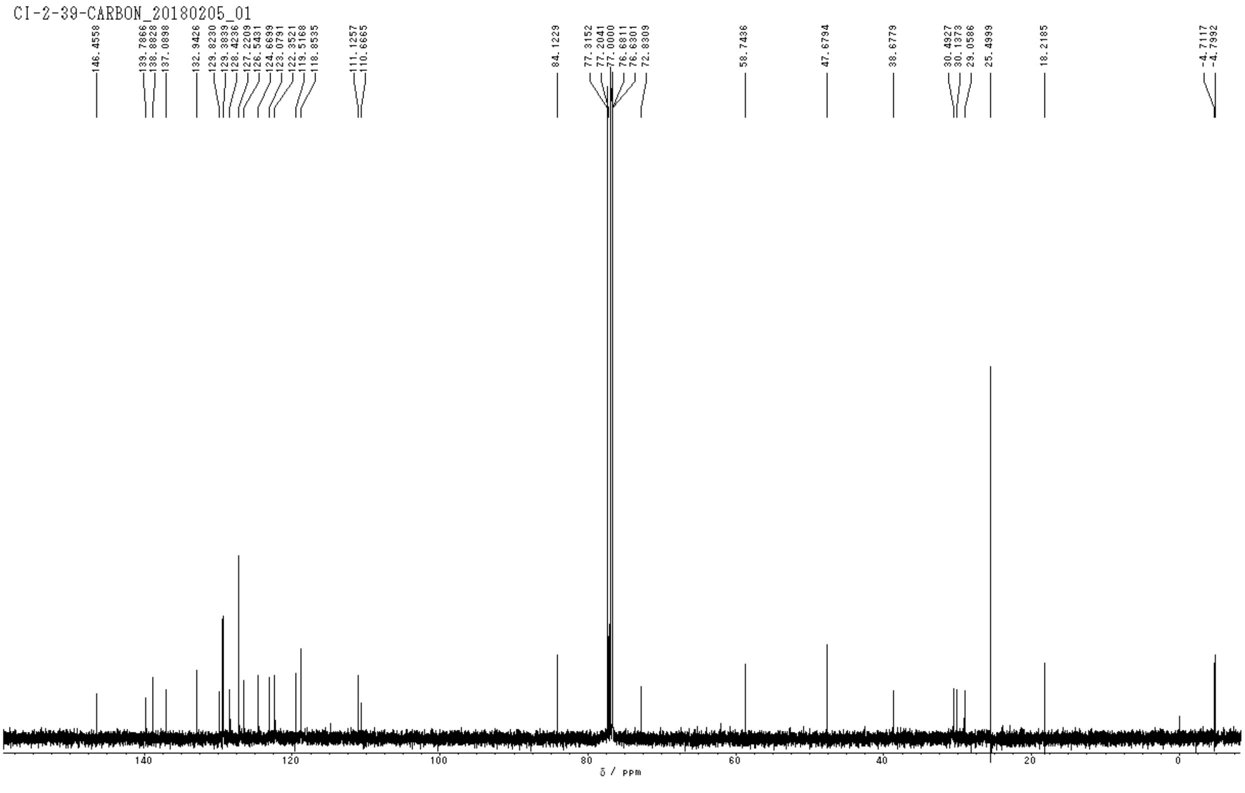
CI-2-39-CARBON_20180205_01
δ / ppm

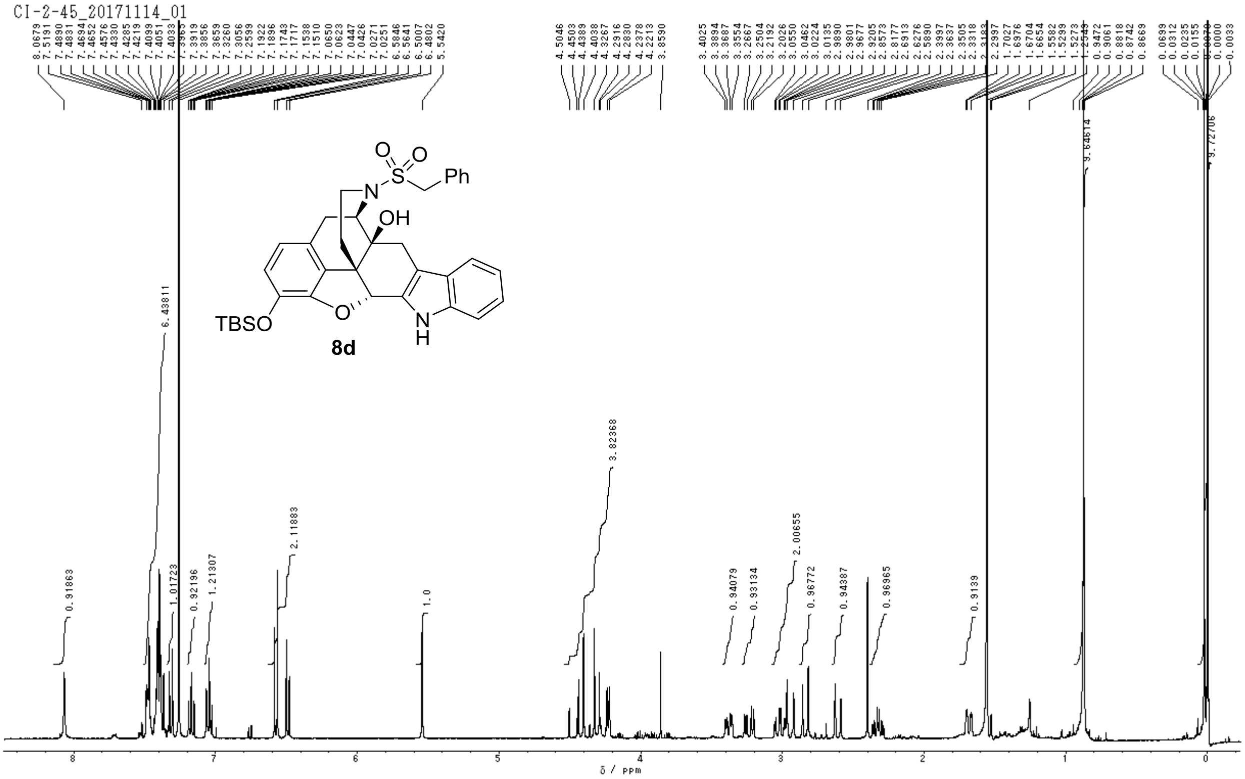
CI-2-45_20171114_01
TBSO
OH
Ph
8d
δ / ppm

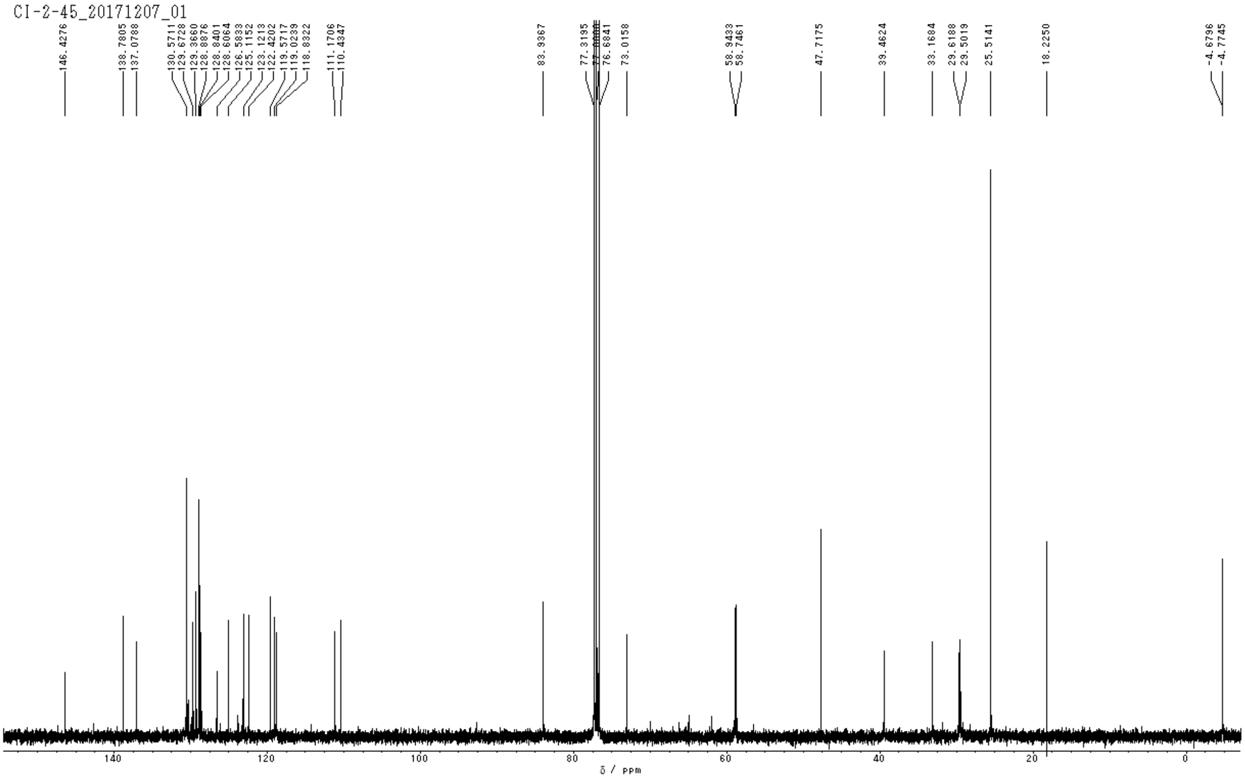
CI-2-45_20171207_01
δ / ppm

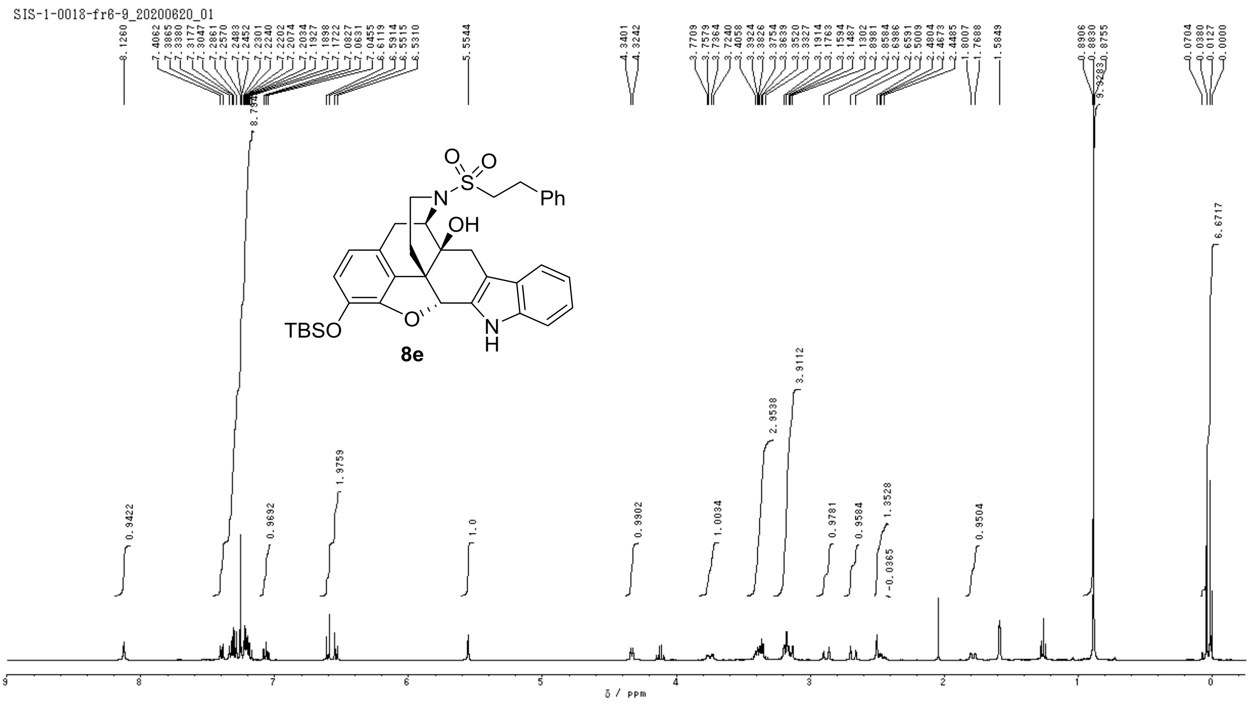
SIS-1-0018-fr6-9_20200620_01
TBSO
OH
Ph
8e
δ / ppm

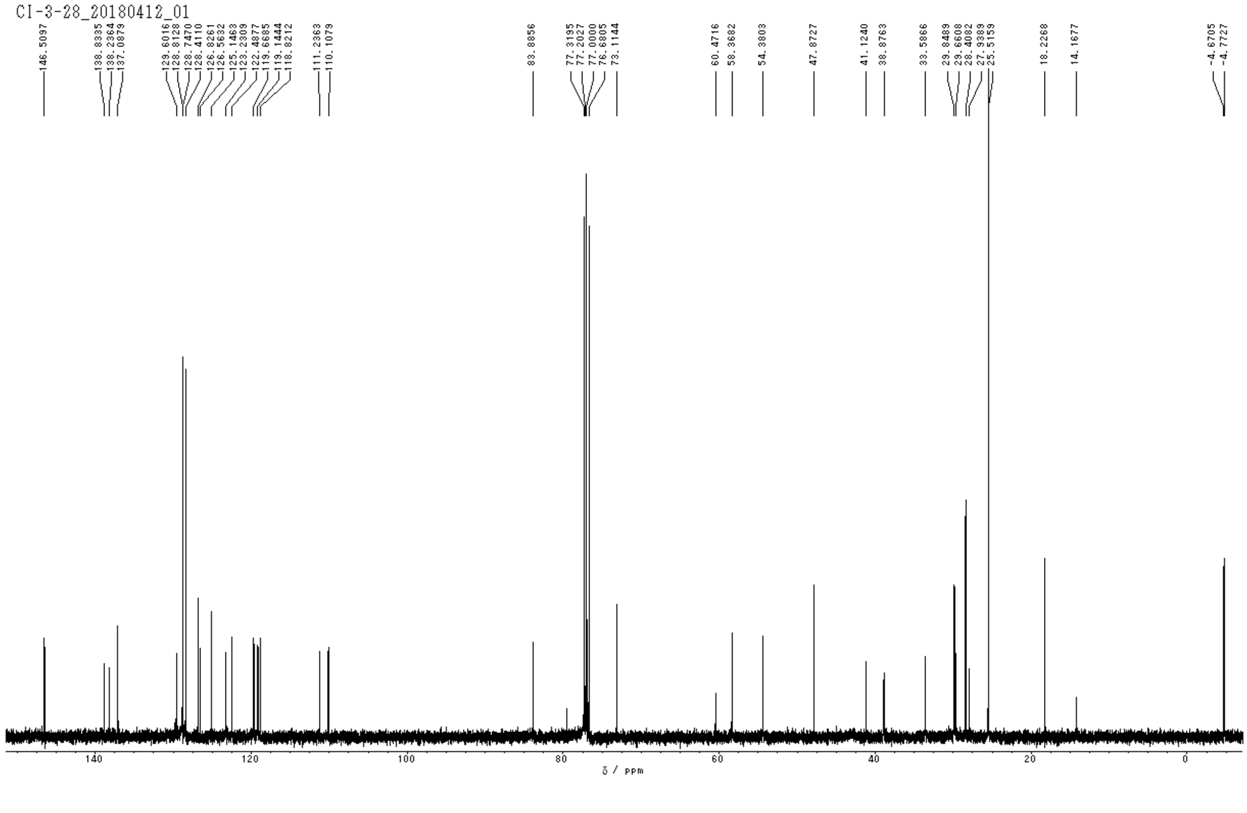
CI-3-28_20180412_01
δ / ppm

CI-2-56-proton_20171117_01

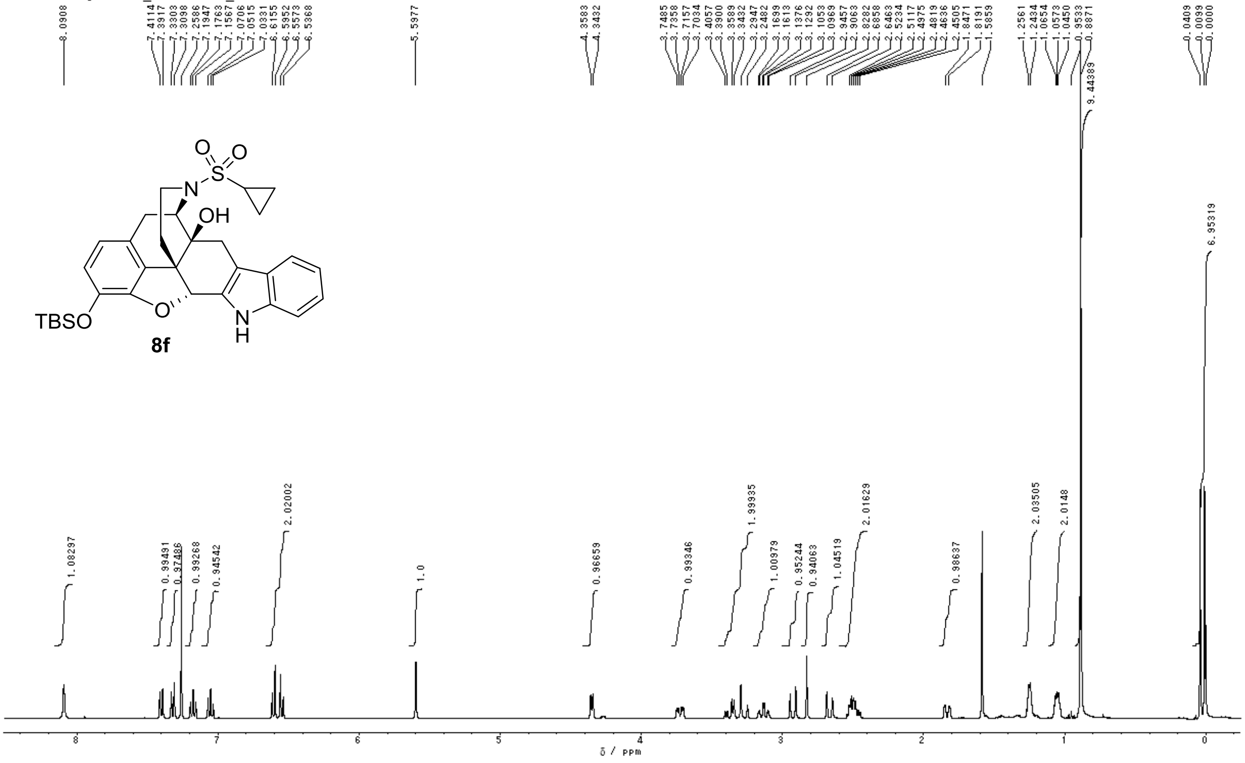

CI-2-56-Carbon_20171117_01

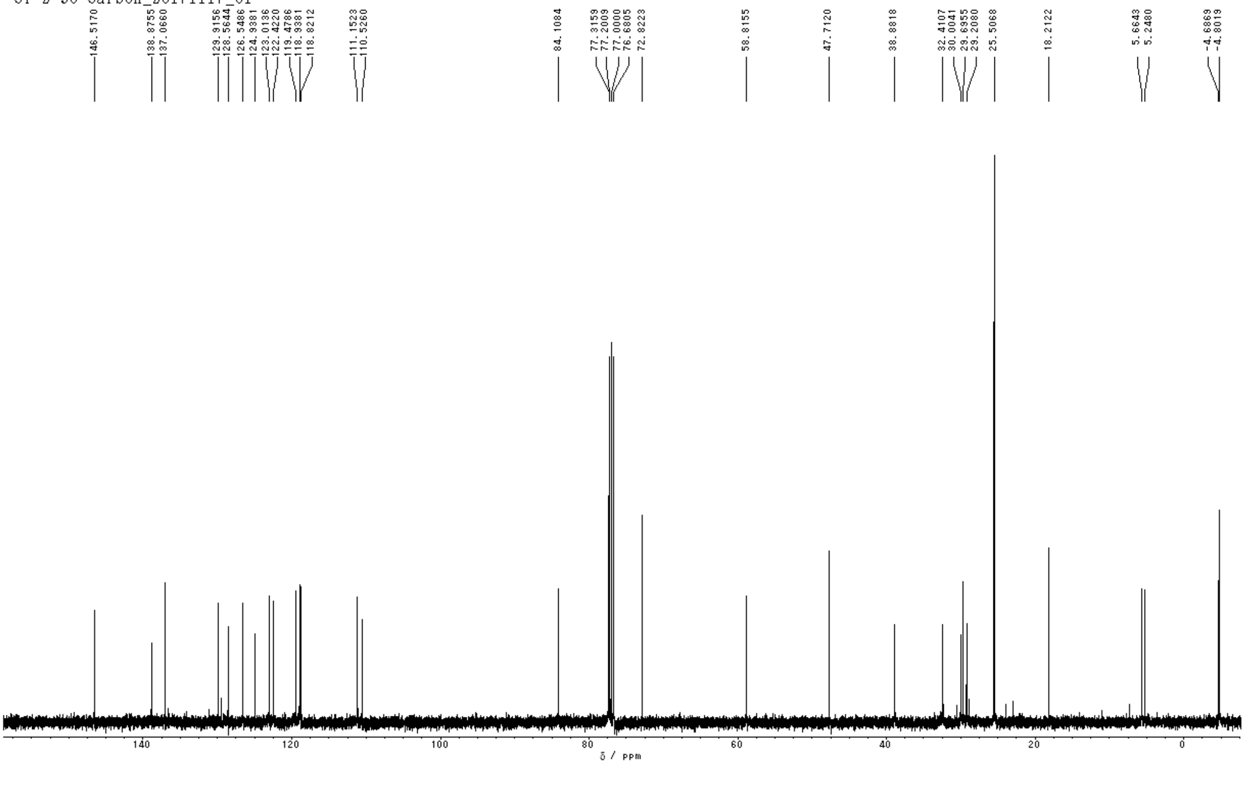

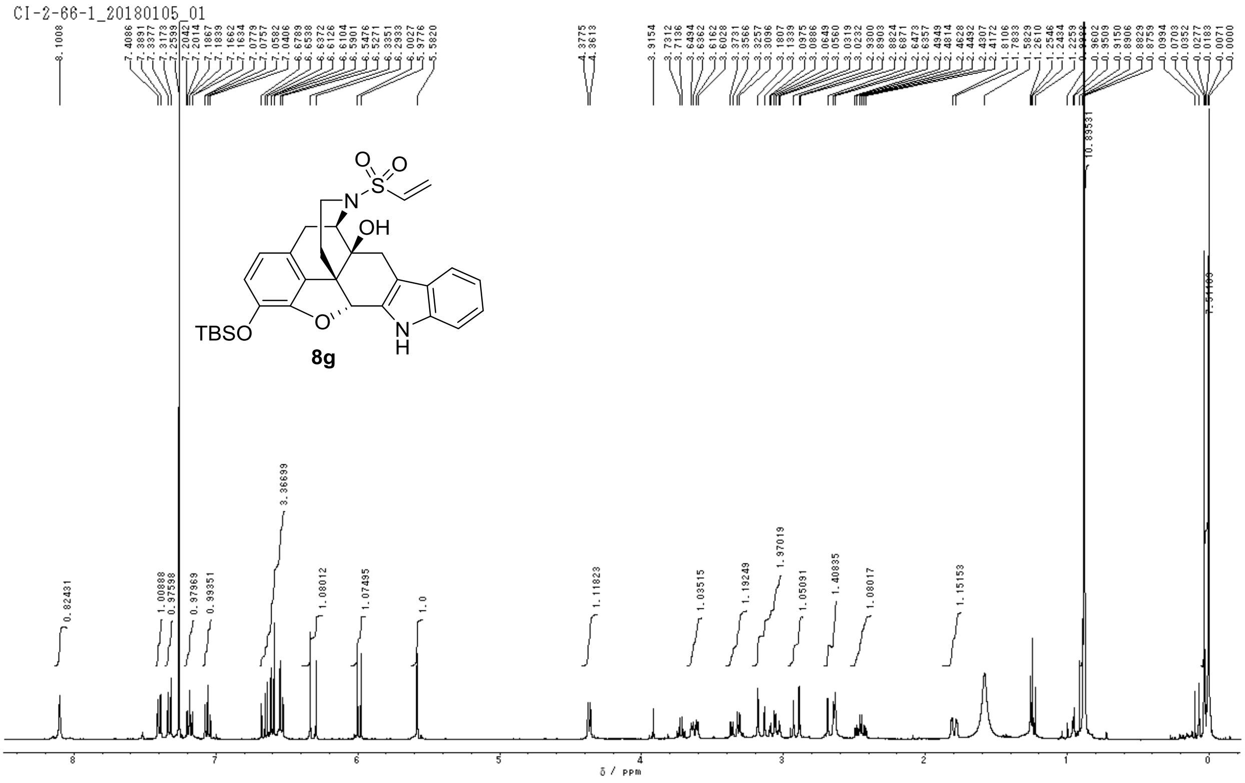
CI-2-66-1_20180105_01
8g
TBSO
OH
N
S
O
NH
0.82431
1.00888
0.97598
0.97969
0.99351
3.36699
1.08012
1.07495
1.0
1.11823
1.03515
1.19249
1.97019
1.05091
1.40835
1.08017
1.15153
10.89531
δ / ppm

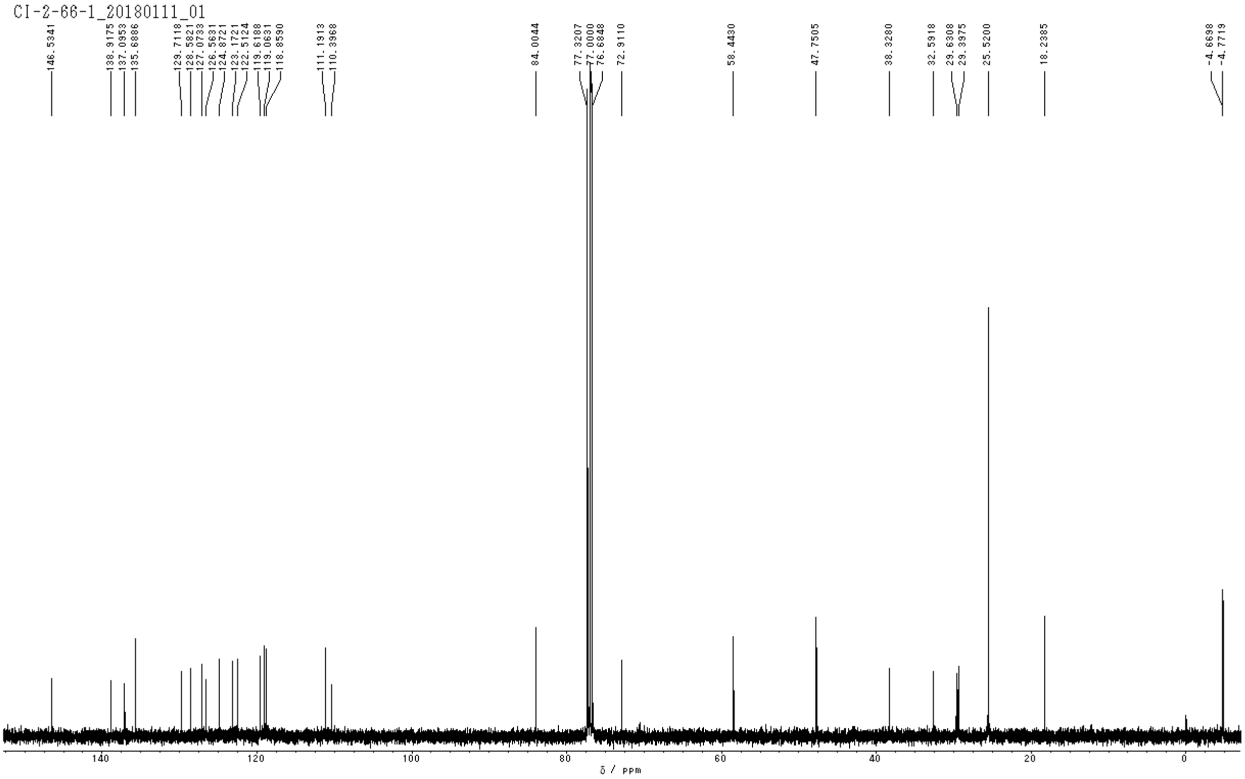
CI-2-66-1_20180111_01
146.5341
138.9175
137.0953
135.6886
129.7118
128.5821
127.0733
126.5631
124.8721
123.1721
122.5124
119.6188
119.0631
118.8590
111.1913
110.3968
84.0044
77.3207
77.0000
76.6848
72.9110
58.4430
47.7505
38.3280
32.5918
29.6308
29.3975
25.5200
18.2385
-4.6698
-4.7719
δ / ppm

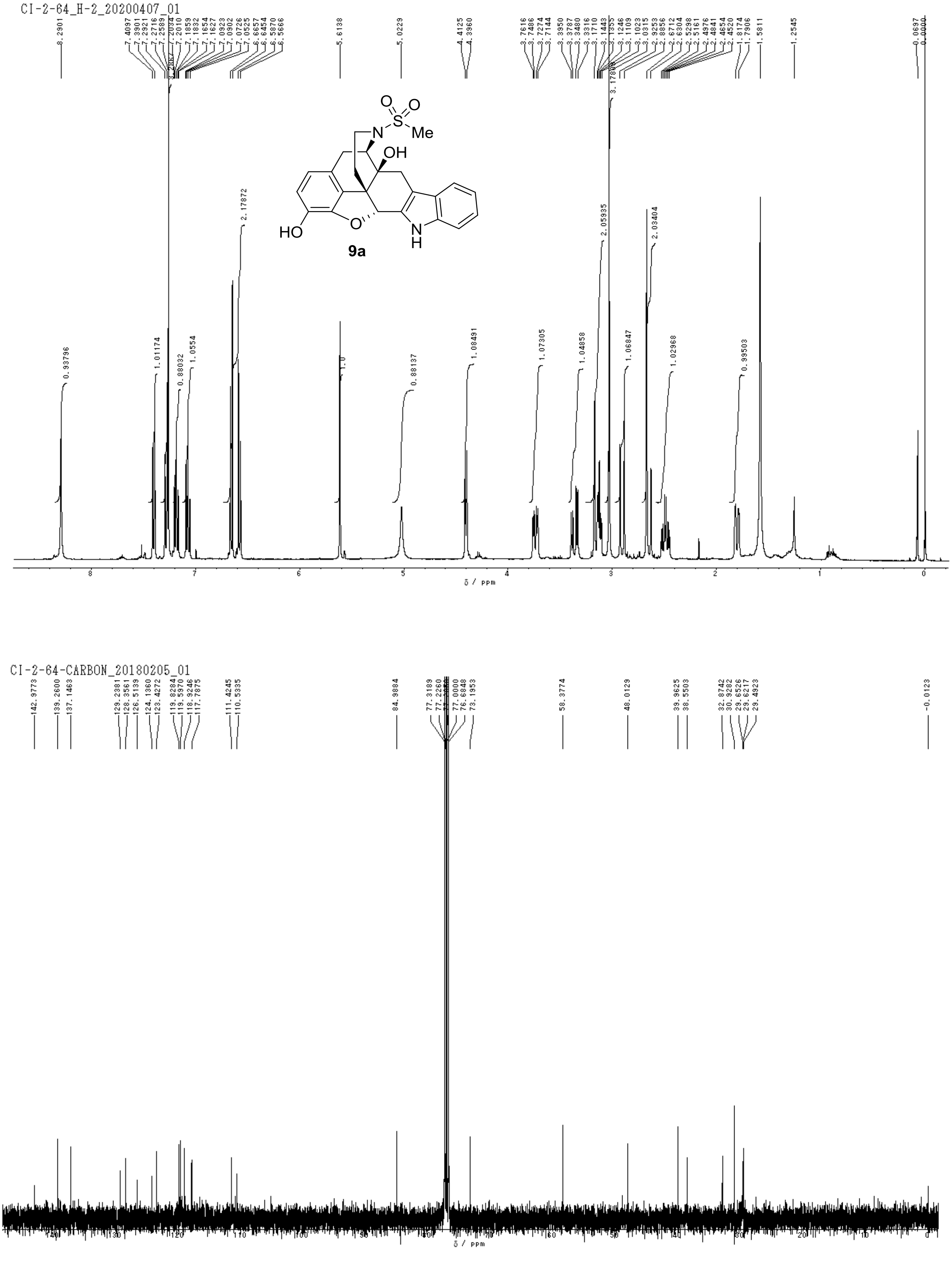
CI-2-64_H-2_20200407_01
9a
δ / ppm
CI-2-64-CARBON_20180205_01
δ / ppm

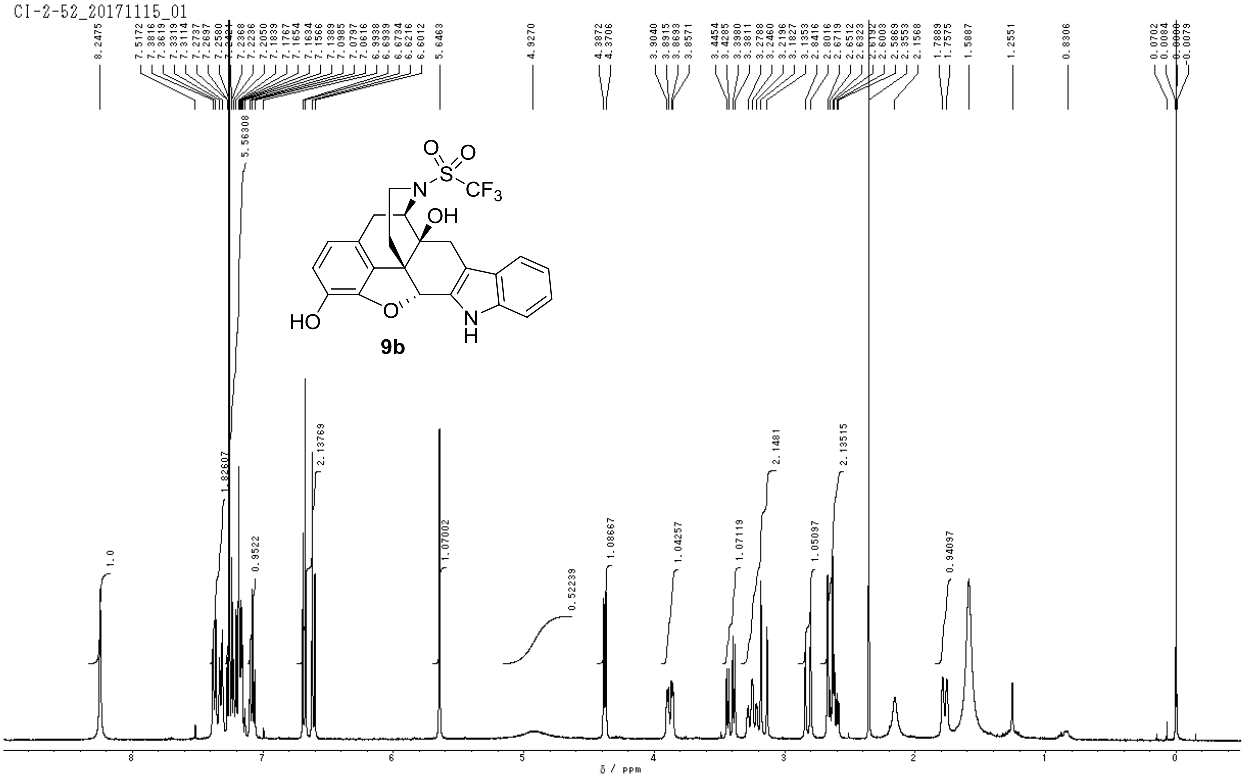
CI-2-52_20171115_01
8.2475
7.5172 7.3816 7.3619 7.3313 7.3114 7.2737 7.2697 7.2580 7.2368 7.2286 7.2050 7.1839 7.1767 7.1654 7.1634 7.1566 7.1389 7.0985 7.0797 7.0616 6.9938 6.6939 6.6734 6.6216 6.6012
5.6463
4.9270
4.3872 4.3706
3.9040 3.8915 3.8693 3.8571
3.4454 3.4285 3.3980 3.3811 3.2788 3.2460 3.2196 3.1827 3.1353 2.8416 2.8016 2.6719 2.6512 2.6323 2.6192 2.6003 2.5889 2.3553 2.1568
1.7889 1.7575 1.5887
1.2551
0.8306
0.0702 0.0084 0.0000 -0.0079
OH
CF3
HO
9b
1.0
1.82607
5.56308
0.9522
2.13769
1.07002
0.52239
1.08667
1.04257
1.07119
2.1481
1.05097
2.13515
0.94097
δ / ppm

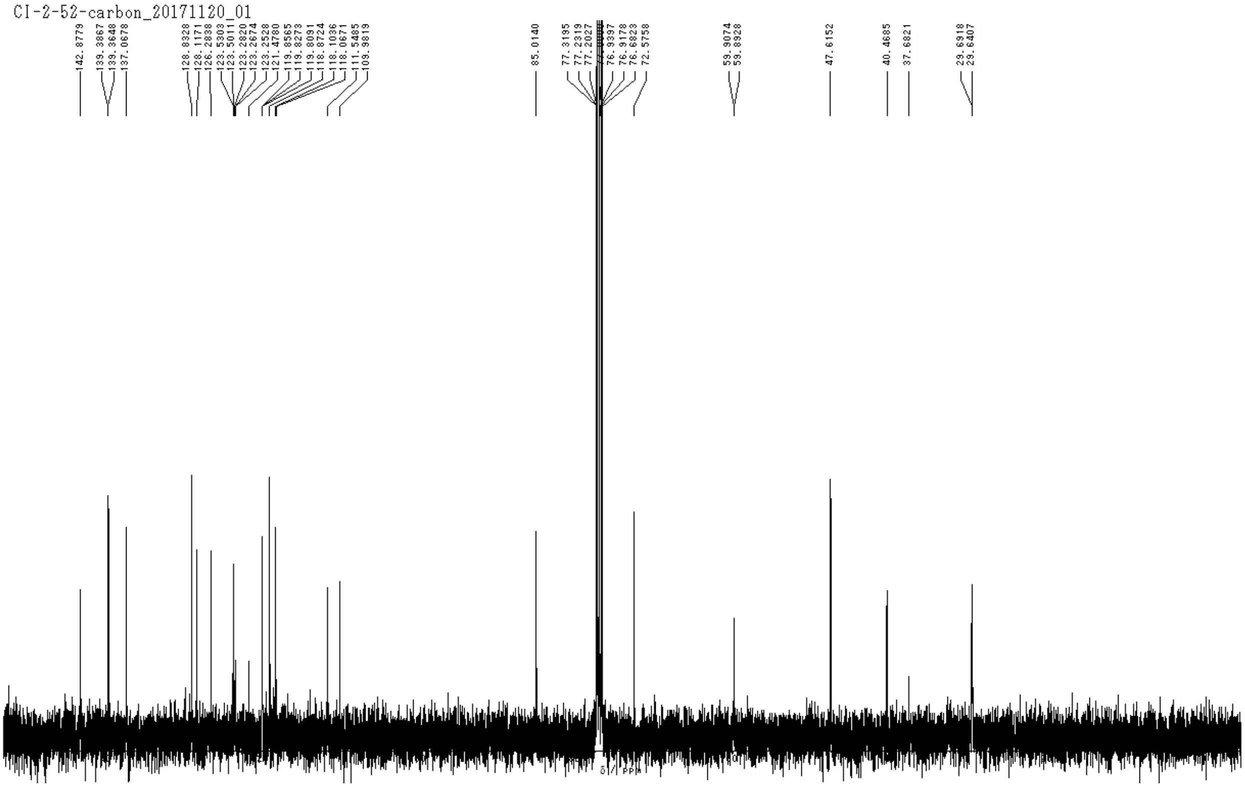
CI-2-52-carbon_20171120_01
142.8779 139.3867 139.3648 137.0678
128.8328 128.1171 126.2838 123.5303 123.5011 123.2820 123.2674 123.2528 121.4780 119.8565 119.8273 119.8091 118.8724 118.1036 118.0671 111.5485 109.9819
85.0140
77.3195 77.2319 77.2027 76.3397 76.9178 76.6823 72.5758
59.9074 59.8928
47.6152
40.4685 37.6821
29.6918 29.6407

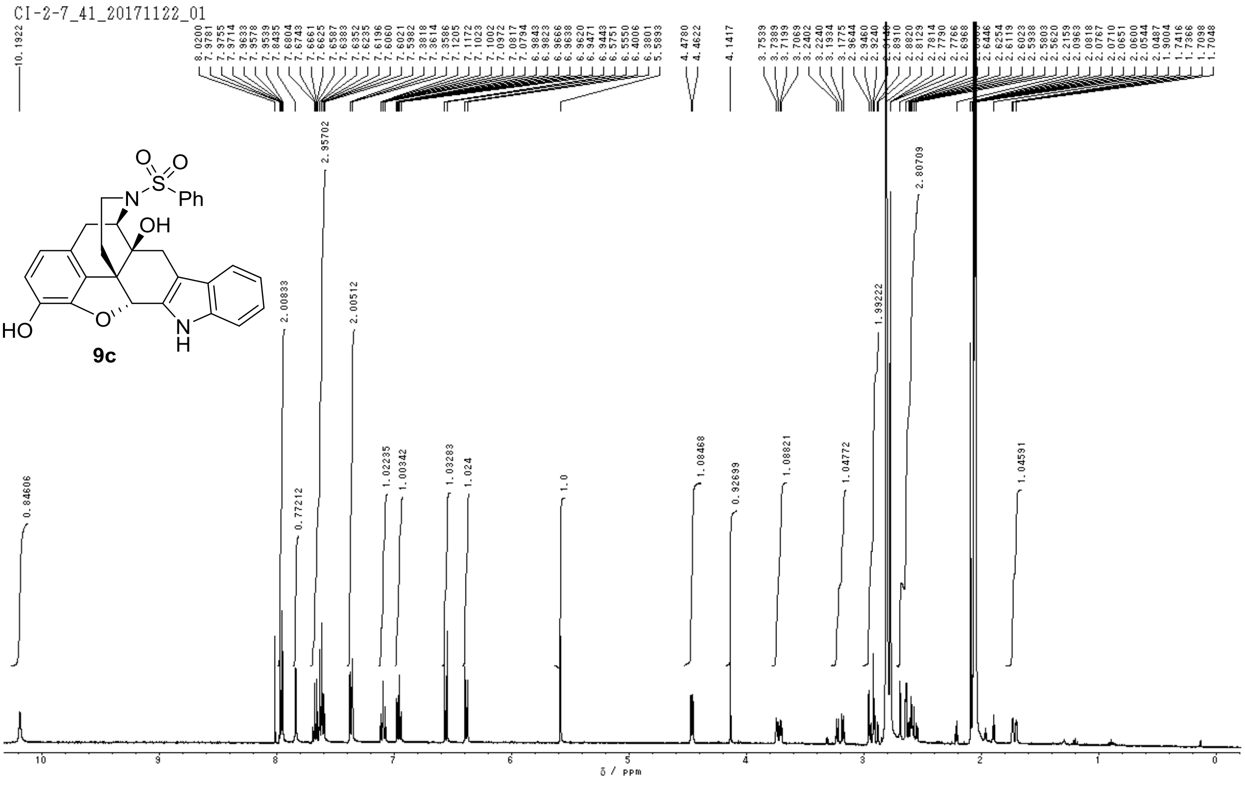
CI-2-7_41_20171122_01
10.1922
8.0200 7.9781 7.9755 7.9714 7.9633 7.9578 7.9539 7.8435 7.6804 7.6743 7.6661 7.6625 7.6597 7.6383 7.6352 7.6235 7.6196 7.6060 7.6021 7.5982 7.3818 7.3614 7.3586 7.1205 7.1172 7.1023 7.1002 7.0972 7.0817 7.0794 6.9843 6.9823 6.9666 6.9638 6.9620 6.9471 6.9443 6.5751 6.5550 6.4006 6.3801 5.5893
4.4780 4.4622
4.1417
3.7539 3.7389 3.7199 3.7069 3.2402 3.2240 3.1934 3.1775 2.9644 2.9460 2.9240 2.8910 2.8820 2.8129 2.7814 2.7790 2.7766 2.6968 2.6446 2.6254 2.6119 2.6023 2.5938 2.5803 2.5620 2.2159 2.0963 2.0818 2.0767 2.0710 2.0651 2.0600 2.0544 2.0487 1.9004 1.7416 1.7366 1.7098 1.7048
0.84606
2.00833
0.77212
2.95702
2.00512
1.02235
1.00342
1.03283
1.024
1.0
1.08468
0.92699
1.08821
1.04772
1.99222
2.80709
1.04591
O
O
S
N
Ph
OH
HO
O
N
H
9c
10
9
8
7
6
5
4
3
2
1
0
δ / ppm

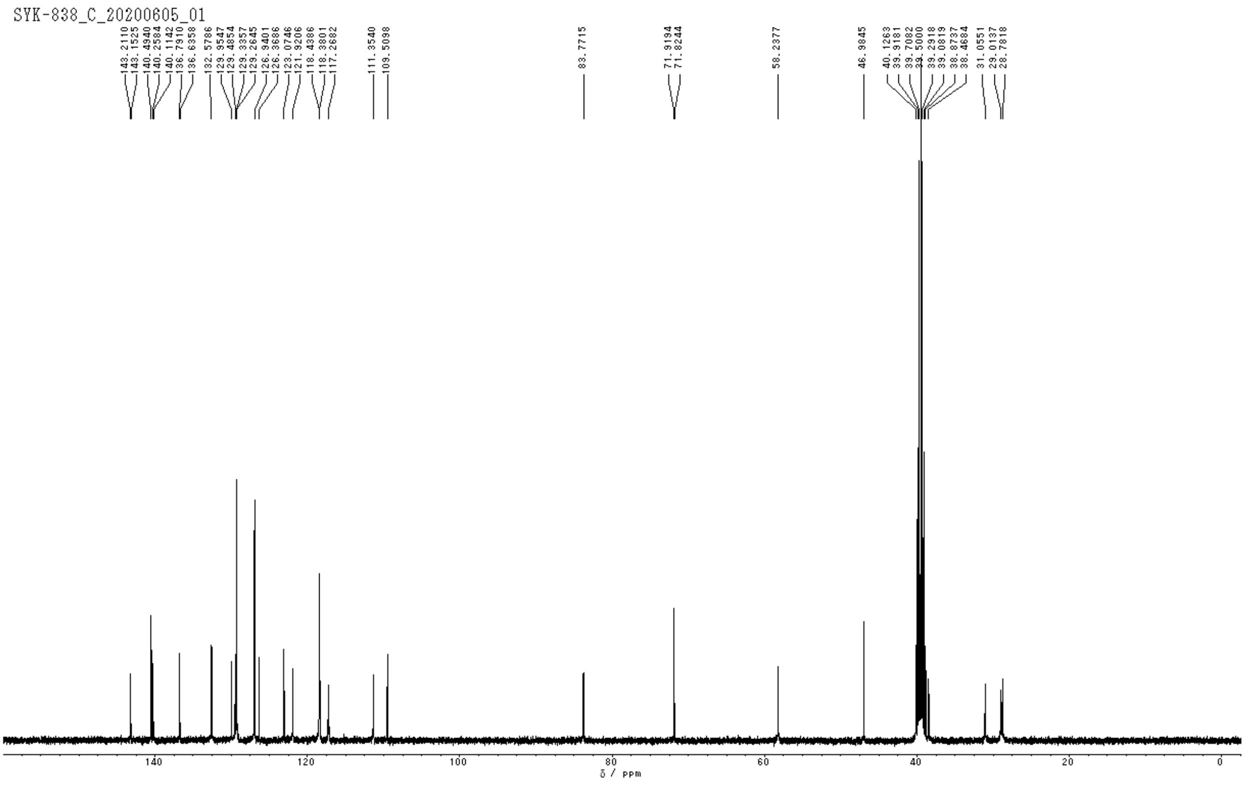
SYK-838_C_20200605_01
143.2110 143.1525 140.4940 140.2584 140.1142 136.7910 136.6358 132.5786 129.9547 129.4854 129.3357 129.2645 126.9401 126.3686 123.0746 121.9206 118.4386 118.3801 117.2682
111.3540 109.5098
83.7715
71.9194 71.8244
58.2377
46.9845
40.1263 39.9181 39.7082 39.5000 39.2918 39.0819 38.8737 38.4684
31.0551 29.0137 28.7818
140
120
100
80
60
40
20
0
δ / ppm

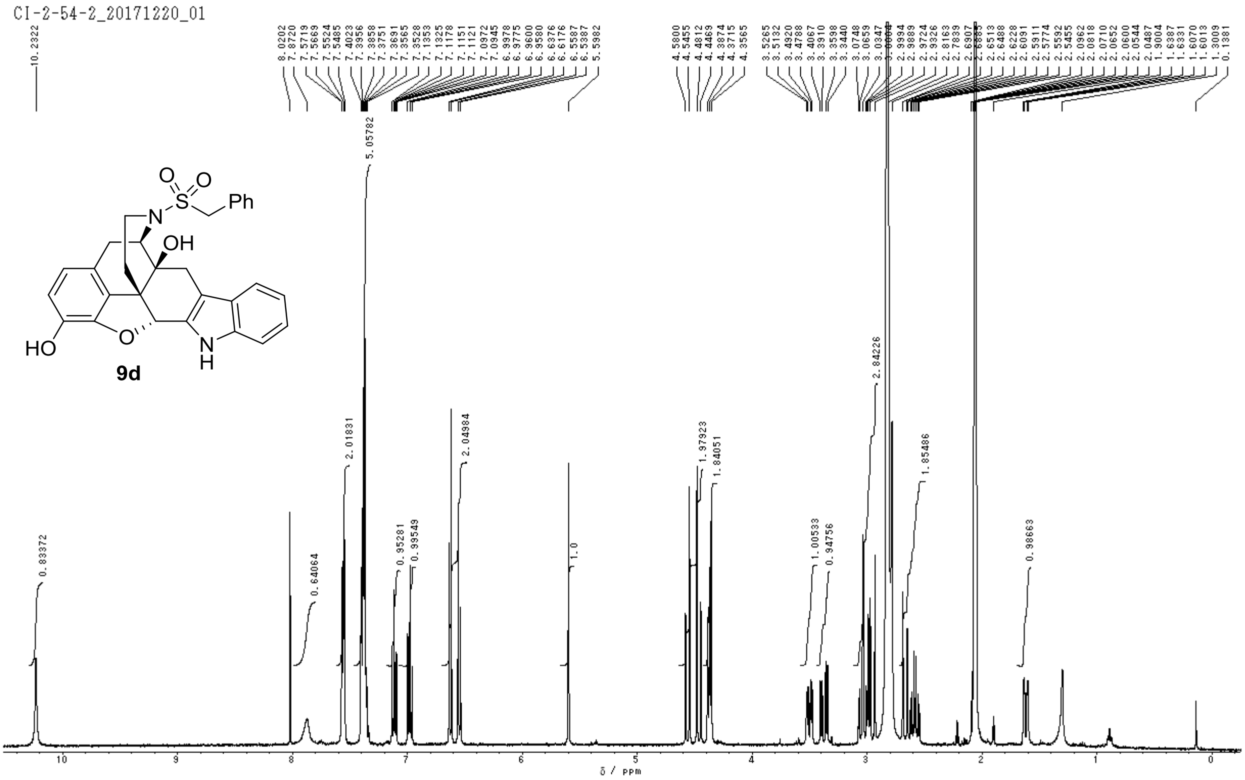
CI-2-54-2_20171220_01
9d
δ / ppm

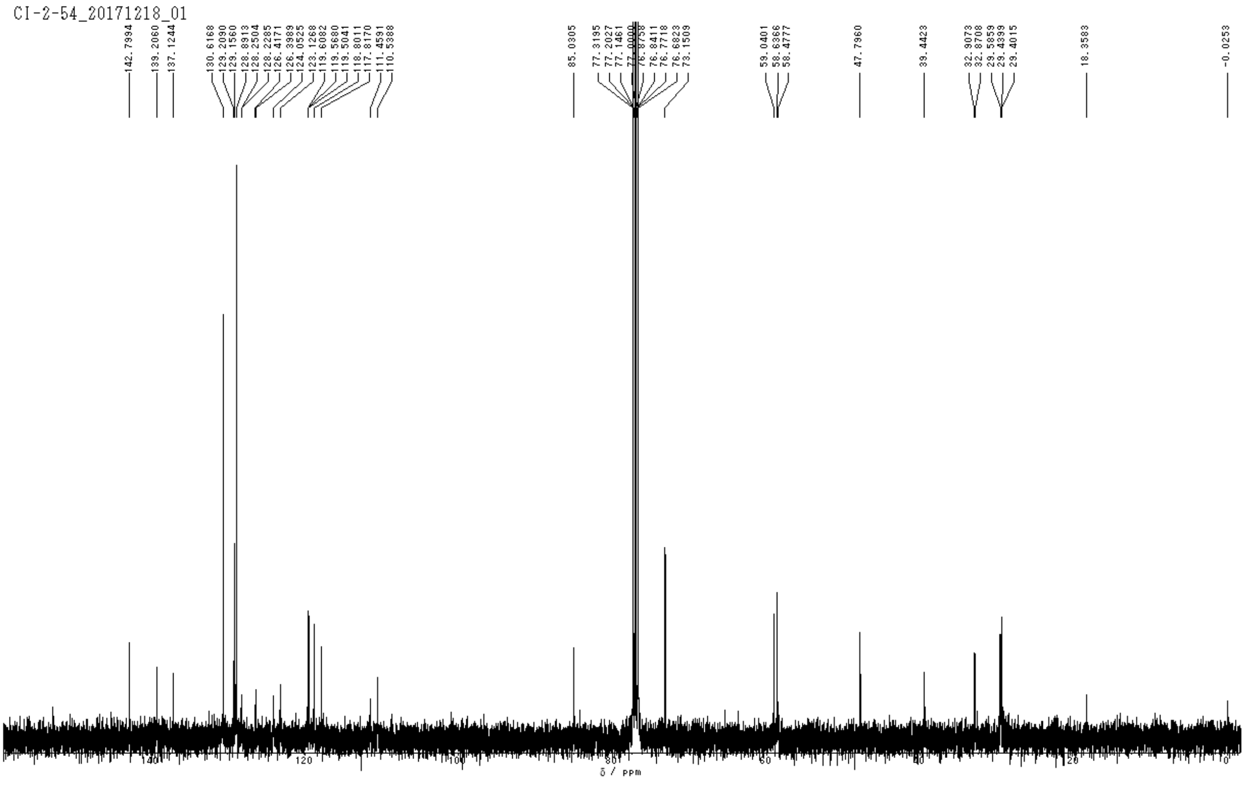
CI-2-54_20171218_01
δ / ppm

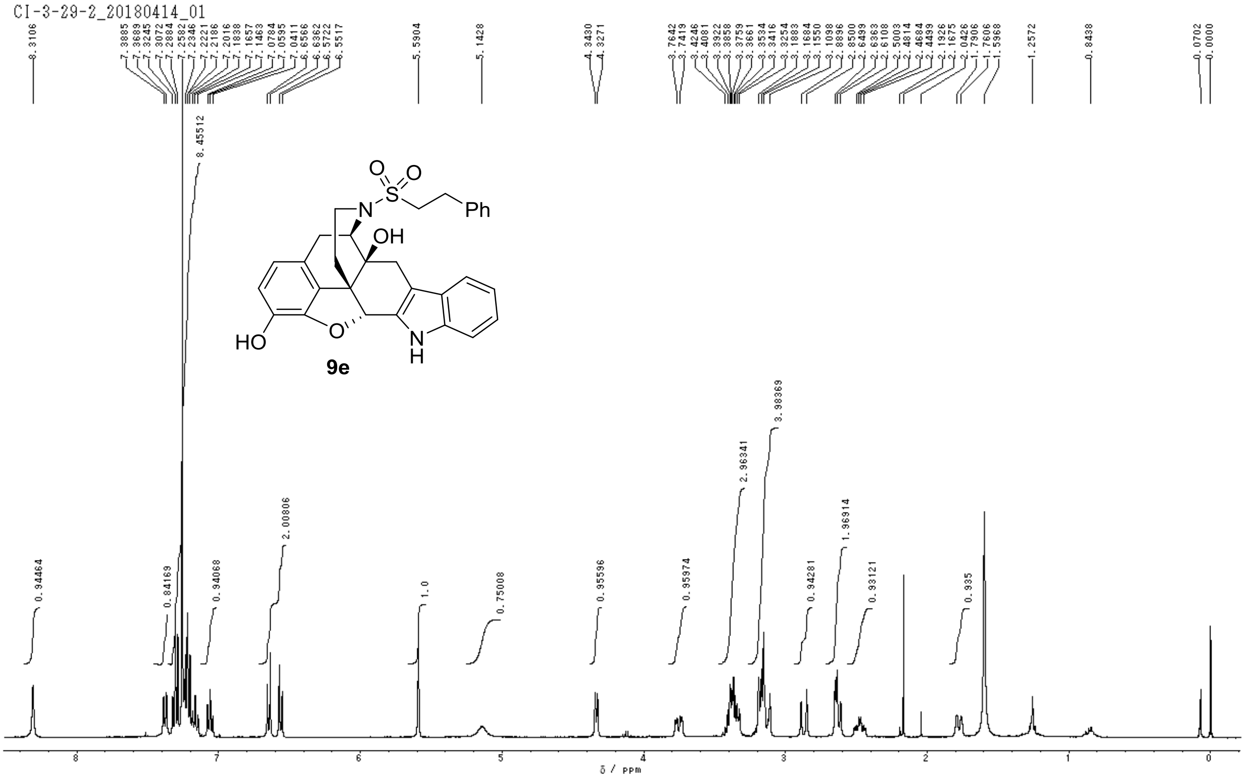
CI-3-29-2_20180414_01
8.3106
7.3885
7.3689
7.3245
7.3072
7.2884
7.2582
7.2346
7.2221
7.2186
7.2016
7.1888
7.1657
7.1463
7.0784
7.0595
7.0411
6.6556
6.6362
6.5722
6.5517
5.5904
5.1428
4.3430
4.3271
3.7642
3.7419
3.4246
3.4081
3.3922
3.3858
3.3759
3.3661
3.3534
3.3416
3.3254
3.1883
3.1684
3.1550
3.1098
2.8898
2.8500
2.6499
2.6363
2.6108
2.5003
2.4814
2.4684
2.4499
2.1926
2.1675
2.0426
1.7906
1.7606
1.5968
1.2572
0.8438
0.0702
0.0000
0.94464
0.84169
8.45512
0.94068
2.00806
1.0
0.75008
0.95596
0.95974
2.96341
3.98369
0.94281
1.96914
0.93121
0.935
O
S
N
Ph
OH
HO
O
N
H
9e
δ / ppm

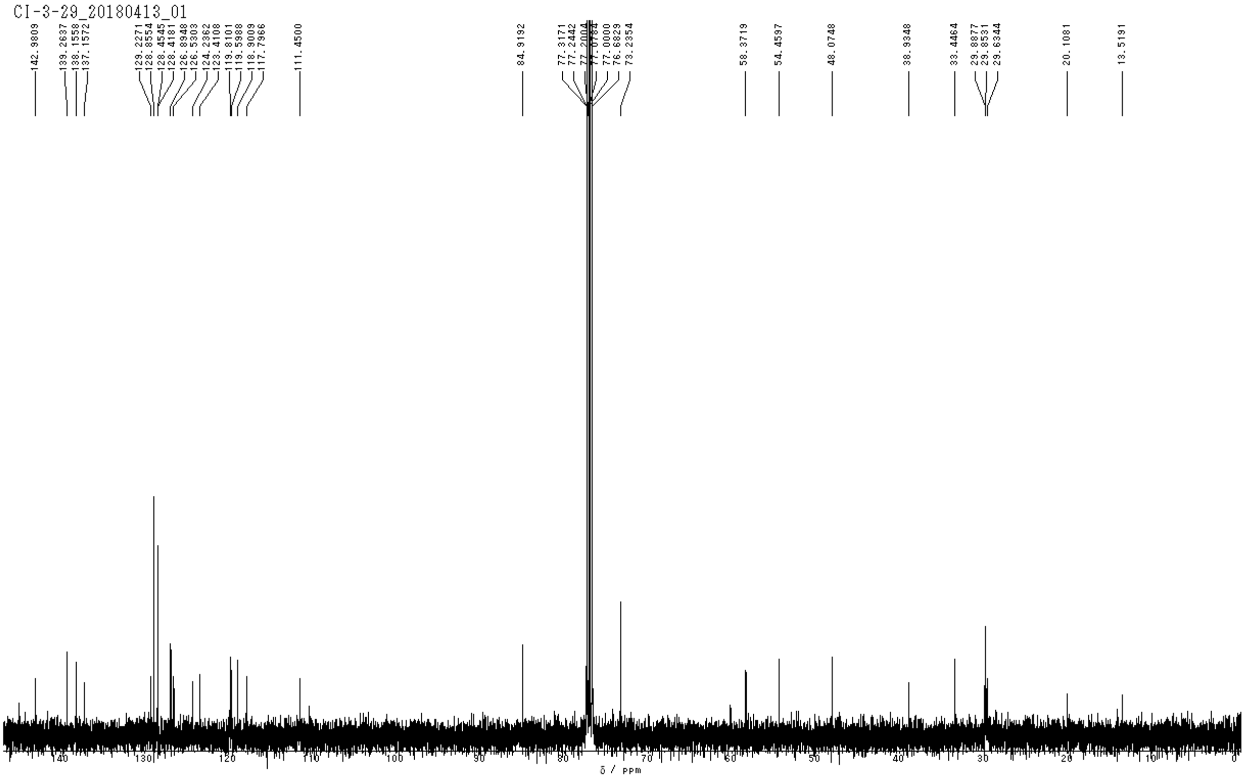
CI-3-29_20180413_01
142.9809
139.2637
138.1558
137.1572
129.2271
128.8554
128.4545
128.4181
126.8948
126.5303
124.2362
123.4108
119.8101
119.5988
118.9009
117.7966
111.4500
84.9192
77.3171
77.2442
77.2004
77.0784
77.0000
76.6829
73.2354
58.3719
54.4597
48.0748
38.9348
33.4464
29.8877
29.8531
29.6344
20.1081
13.5191
δ / ppm

CI-2-58_20171120_01

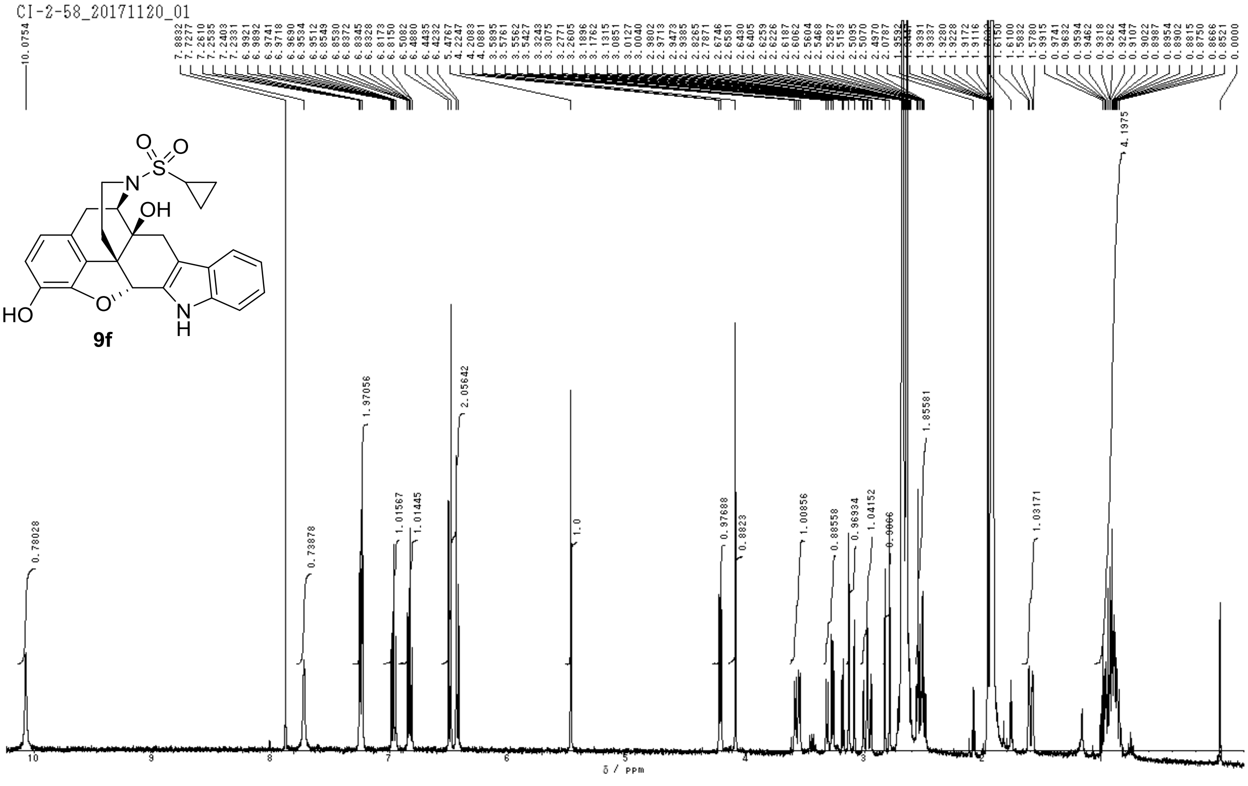

CI-2-40-CARBON_20180205_01

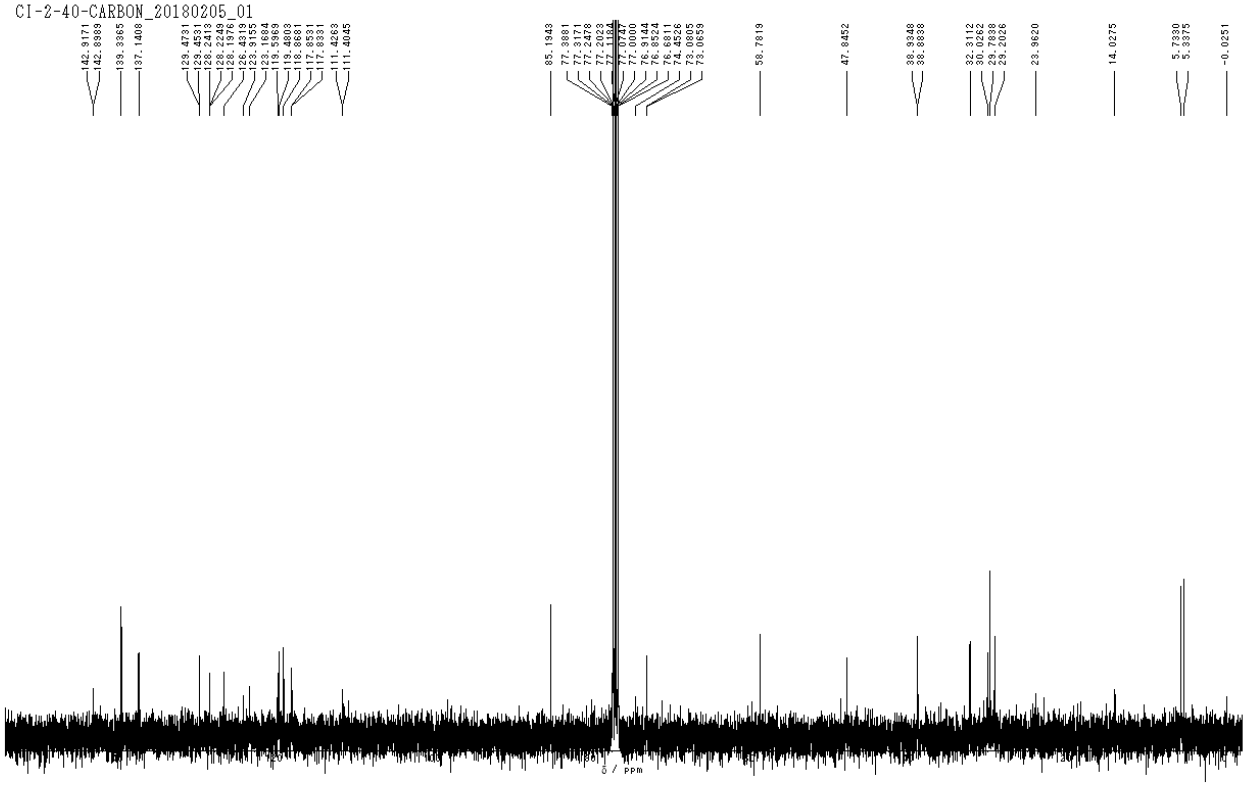

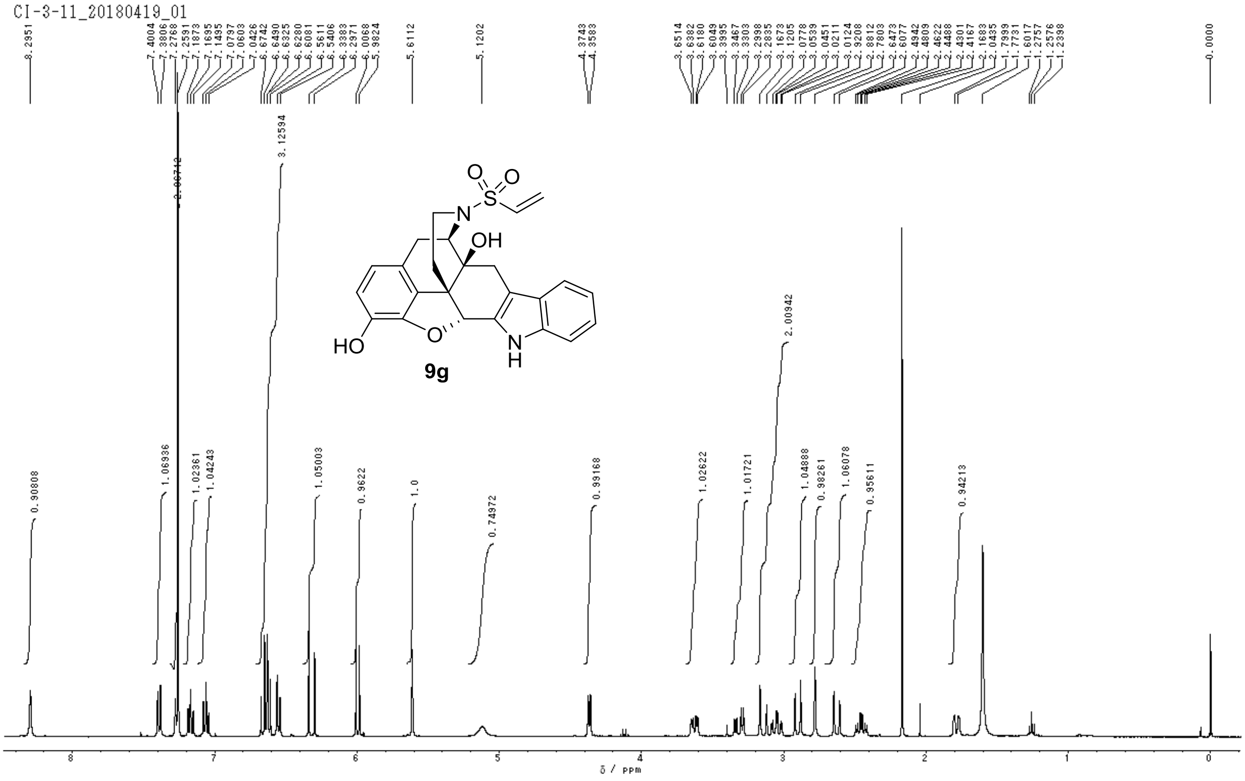
CI-3-11_20180419_01
9g
δ / ppm

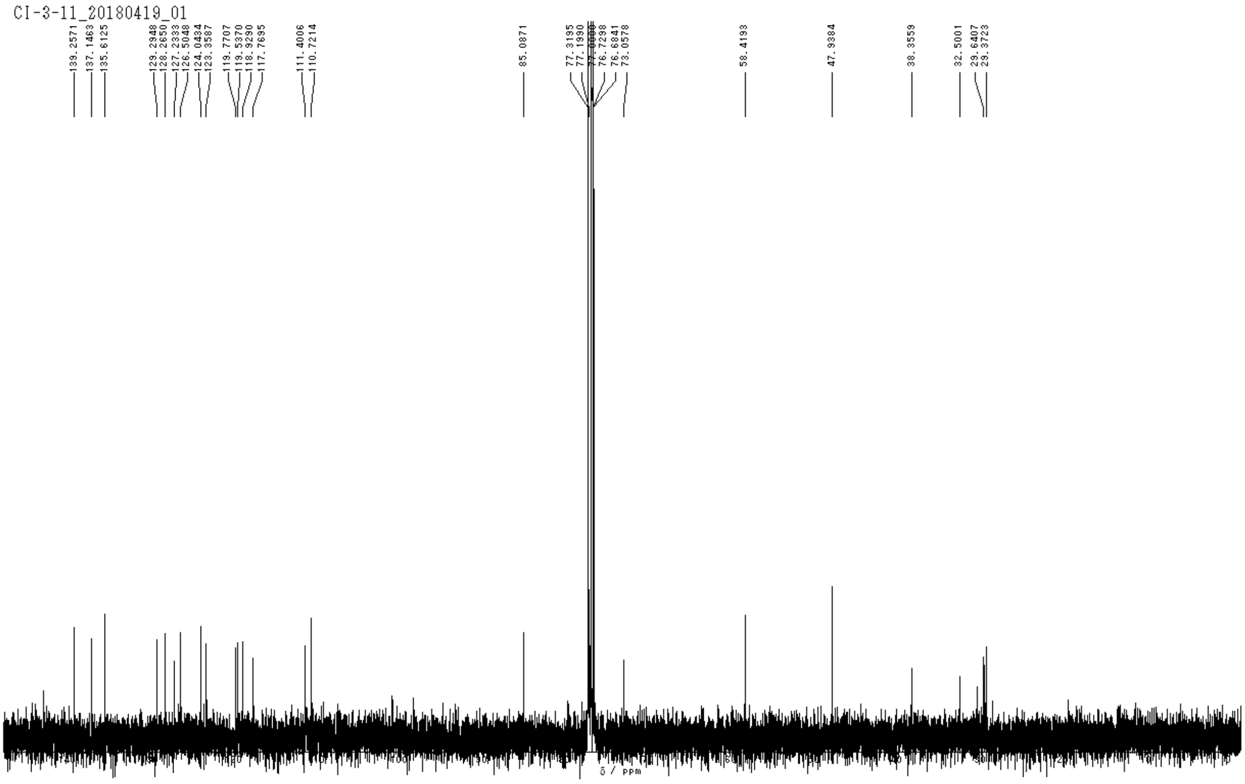
CI-3-11_20180419_01
δ / ppm

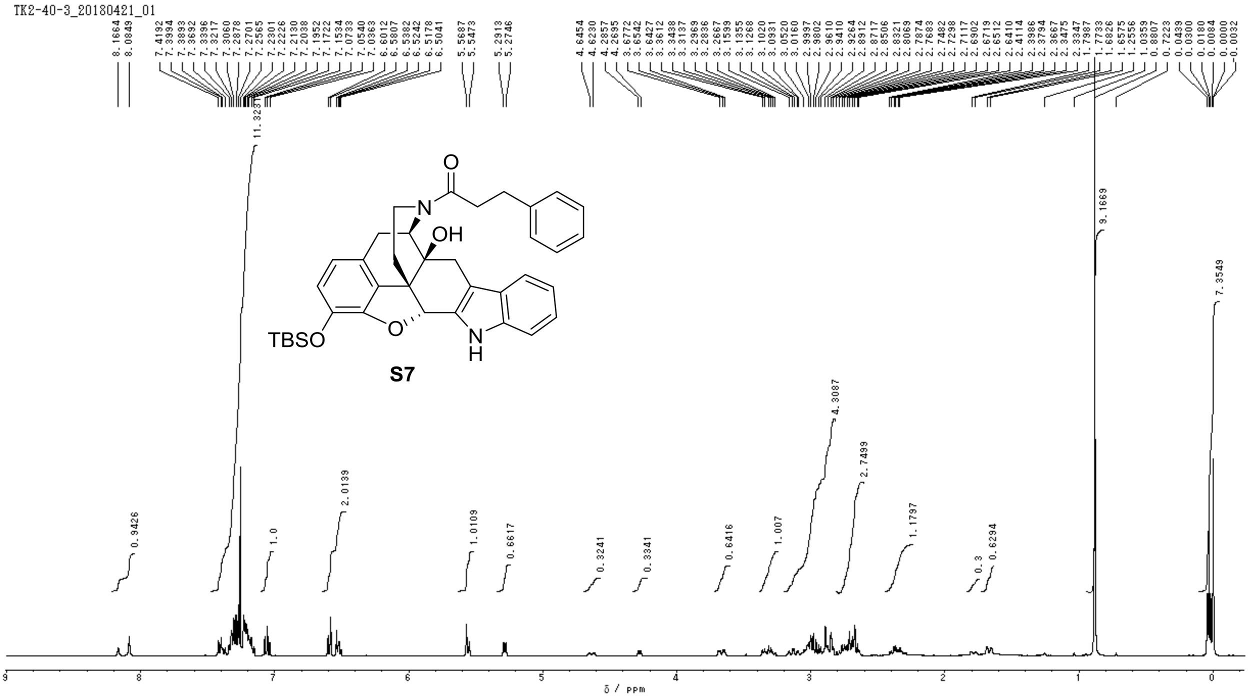
TK2-40-3_20180421_01
TBSO
OH
N
O
NH
S7
δ / ppm

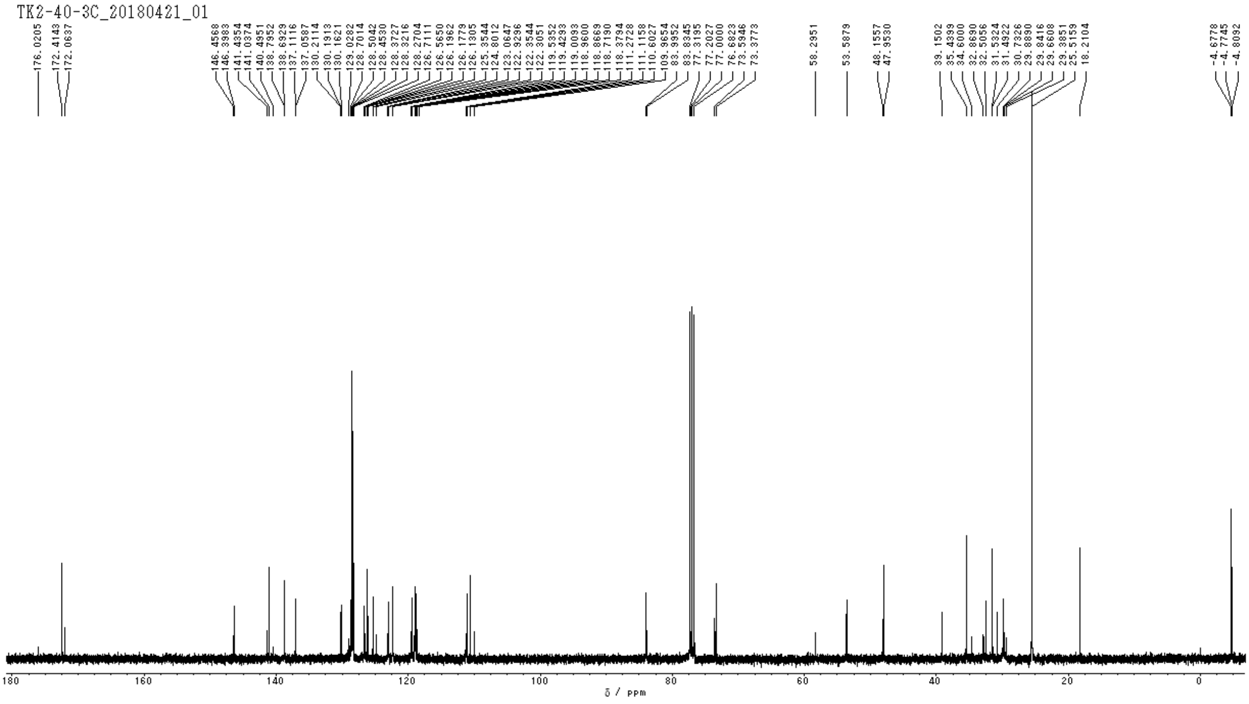
TK2-40-3C_20180421_01
δ / ppm

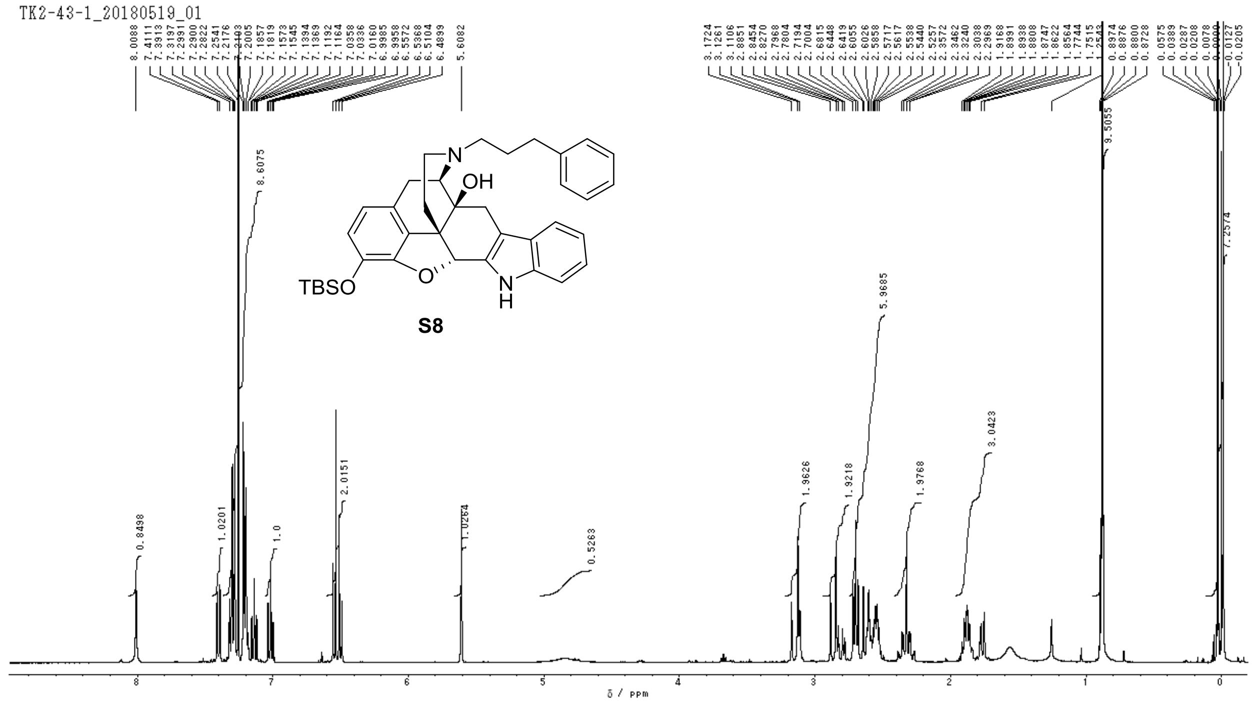
TK2-43-1_20180519_01
N
OH
TBSO
O
N
H
S8
δ / ppm

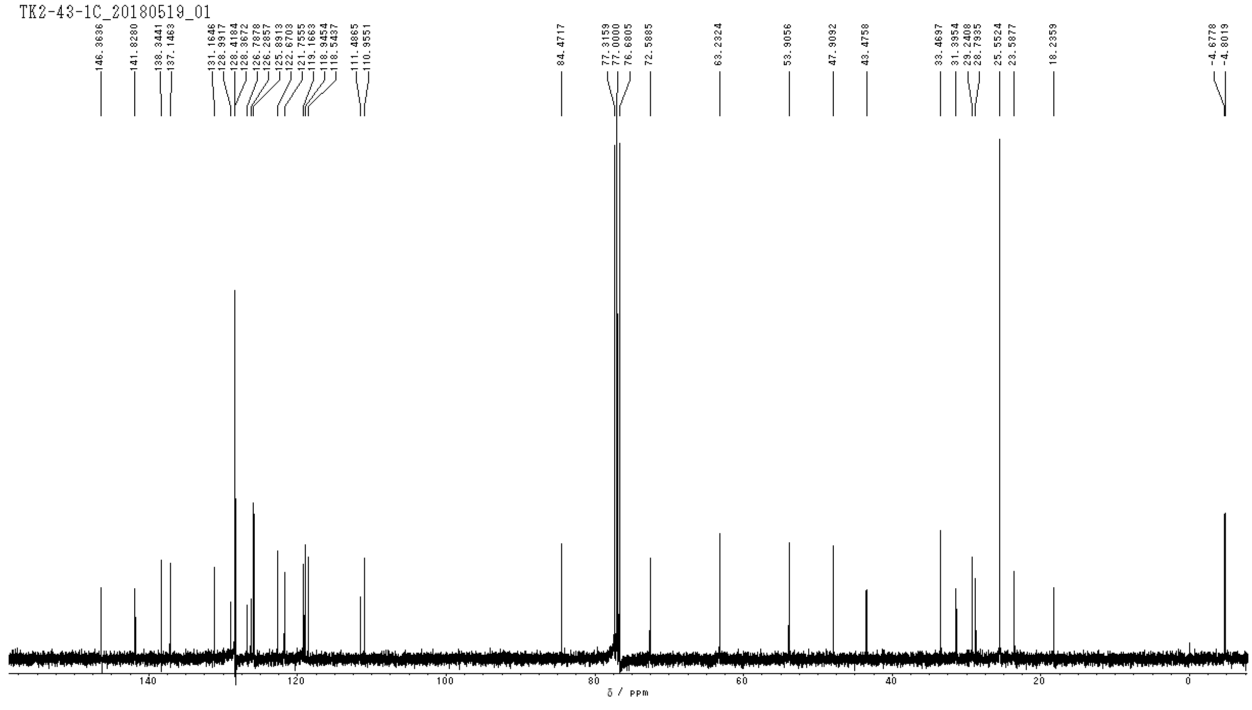
TK2-43-1C_20180519_01
δ / ppm

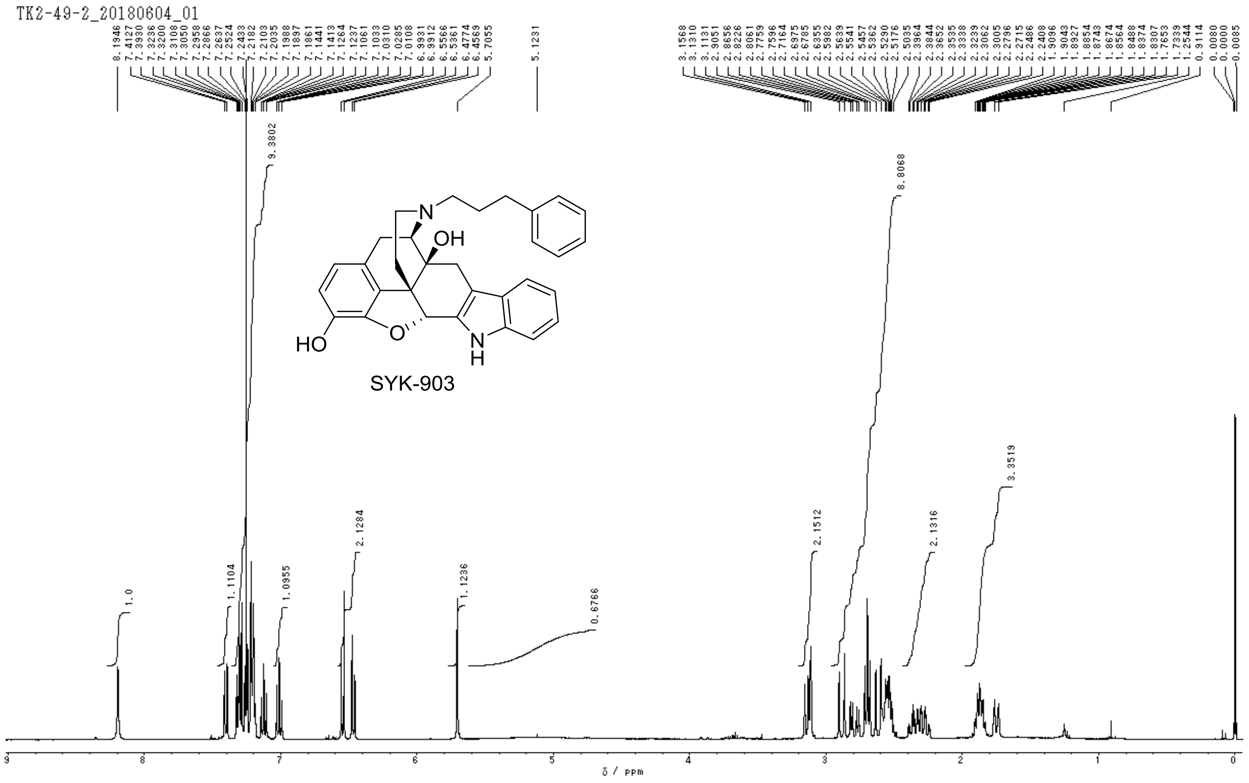
TK2-49-2_20180604_01
SYK-903
δ / ppm

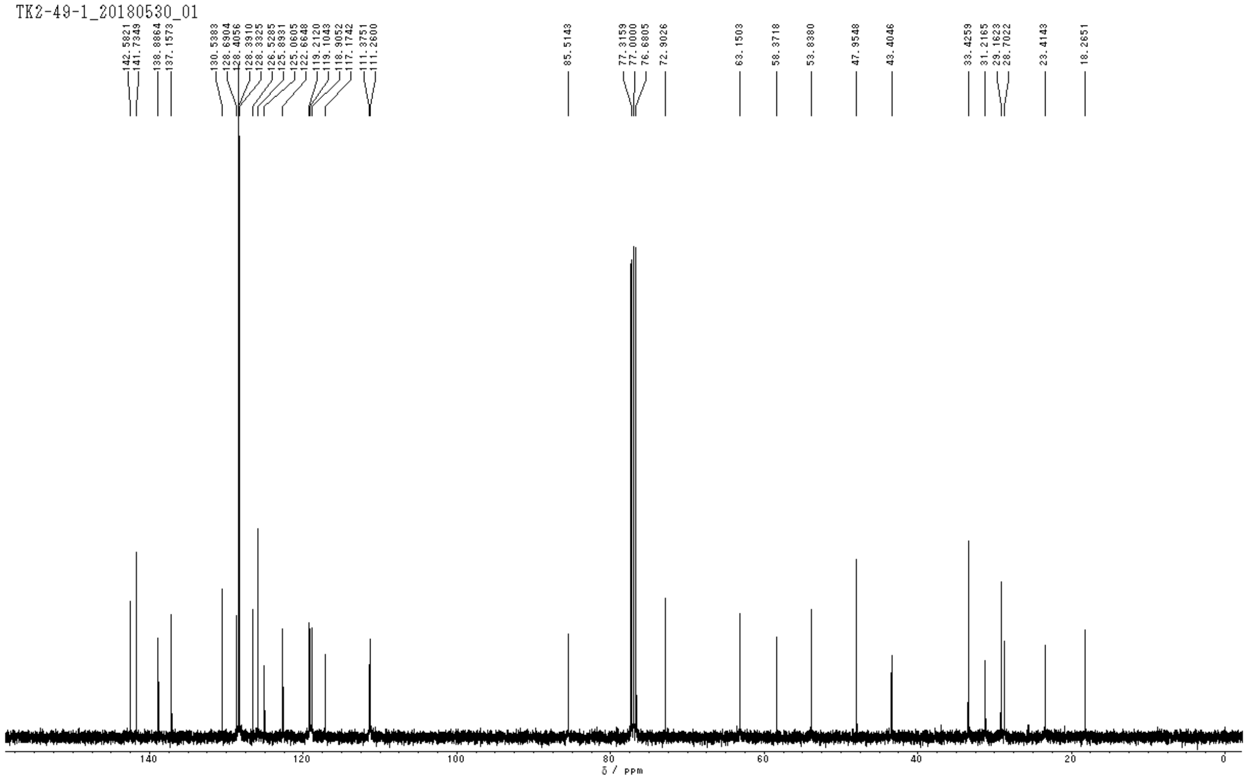
TK2-49-1_20180530_01
δ / ppm

6. References

1. Portoghese, P. S.; Larson, D. L.; Sultana, M.; Takemori, A. E. Opioid Agonist and Antagonist Activities of Morphindoles Related to Naltrindole. *J. Med. Chem.* **1992**, *35*, 4325−4329.

2. McLamore, S.; Ullrich, T.; Rothman, R. B.; Xu, H.; Dersch, C.; Coop, A.; Davis, P.; Porreca, F.; Jacobson, A. E.; Rice, K. C. Effect of *N*-Alkyl and *N*-Alkenyl Substituents in Noroxymorphindole, 17-Substituted-6,7-dehydro-4,5α-epoxy-3,14-dihydroxy-6,7:2′,3′-indolomorphinans, on Opioid Receptor Affinity, Selectivity, and Efficacy. *J. Med. Chem.* **2001**, *44*, 1471−1474.

3. Hirayama, S.; Iwai, T.; Higashi, E.; Nakamura, M.; Iwamatsu, C.; Itoh, K.; Nemoto, T.; Tanabe, M.; Fujii, H. Discovery of δ opioid receptor full inverse agonists and their effects on restraint stress induced cognitive impairment in mice. *ACS Chem. Neurosci.* **2019**, *10*, 2237−2242.

4. Cheng, C.-Y.; Hsin, L.-W.; Lin, Y.-P.; Tao, P.-L.; Jong, T.-T. *N*-Cubylmethyl Substituted Morphinoids as Novel Narcotic Antagonists. *Bioorg. Med. Chem.* **1996**, *4*, 73−80.

5. Nagase, H.; Imaide, S.; Tomatsu, M.; Nemoto, T.; Nakajima, M.; Nakao, K.; Mochizuki, H.; Fujii, H. Investigation of Beckett-Casy model 2: Synthesis of novel 15−16 nornaltrexone derivatives and their pharmacology. *Bioorg. Med. Chem. Lett.* **2010**, *20*, 3726−3729.

6. Nagase, H.; Yamamoto, N.; Yata, M.; Ohrui, S.; Okada, T.; Saitoh, T.; Kutsumura, N.; Nagumo, Y.; Irukayama-Tomobe, Y.; Ishikawa, Y.; et al. Design and Synthesis of Potent and Highly Selective Orexin 1 Receptor Antagonists with a Morphinan Skeleton and Their Pharmacologies. *J. Med. Chem.* **2017**, *60*, 1018−1040.
